# Reciprocal adaptation of rice and *Xanthomonas oryzae pv. oryzae:* cross-species 2D GWAS reveals the underlying genetics

**DOI:** 10.1093/plcell/koab146

**Published:** 2021-06-02

**Authors:** Fan Zhang, Zhiqiang Hu, Zhichao Wu, Jialing Lu, Yingyao Shi, Jianlong Xu, Xiyin Wang, Jinpeng Wang, Fan Zhang, Mingming Wang, Xiaorong Shi, Yanru Cui, Casiana Vera Cruz, Dalong Zhuo, Dandan Hu, Min Li, Wensheng Wang, Xiuqin Zhao, Tianqing Zheng, Binying Fu, Jauhar Ali, Yongli Zhou, Zhikang Li

**Affiliations:** 1 Institute of Crop Sciences/National Key Facility for Crop Gene Resources and Genetic Improvement, Chinese Academy of Agricultural Sciences, 12 South Zhong-Guan-Cun Street, Haidian District, Beijing 100081, China; 2 College of Agronomy, Anhui Agricultural University, 130 West Chang-Jiang Street, Hefei 230036, China; 3 Department of Plant and Microbial Biology, University of California, Berkeley, California 94720, USA; 4 Shenzhen Branch, Guangdong Laboratory for Lingnan Modern Agriculture, Genome Analysis Laboratory of the Ministry of Agriculture, Agricultural Genomics Institute at Shenzhen, Chinese Academy of Agricultural Sciences, Shenzhen, Guangdong 518120, China; 5 School of Life Sciences, North China University of Science and Technology, Tangshan, Hebei 063009, China; 6 International Rice Research Institute, DAPO Box 7777, Metro Manila, The Philippines

## Abstract

A 1D/2D genome-wide association study strategy was adopted to investigate the genetic systems underlying the reciprocal adaptation of rice (*Oryza sativa*) and its bacterial pathogen, *Xanthomonas oryzae* pv. *oryzae* (*Xoo*) using the whole-genome sequencing and large-scale phenotyping data of 701 rice accessions and 23 diverse *Xoo* strains. Forty-seven *Xoo* virulence-related genes and 318 rice quantitative resistance genes (QR-genes) mainly located in 41 genomic regions, and genome-wide interactions between the detected virulence-related genes and QR genes were identified, including well-known resistance genes/virulence genes plus many previously uncharacterized ones. The relationship between rice and *Xoo* was characterized by strong differentiation among *Xoo* races corresponding to the subspecific differentiation of rice, by strong shifts toward increased resistance/virulence of rice/*Xoo* populations and by rich genetic diversity at the detected rice QR-genes and *Xoo* virulence genes, and by genome-wide interactions between many rice QR-genes and *Xoo* virulence genes in a multiple-to-multiple manner, presumably resulting either from direct protein–protein interactions or from genetic epistasis. The observed complex genetic interaction system between rice and *Xoo* likely exists in other crop–pathogen systems that would maintain high levels of diversity at their QR-loci/virulence-loci, resulting in dynamic coevolutionary consequences during their reciprocal adaptation.

## Teaser

A complex system of genetic interactions leads to reciprocal adaptation between rice and its bacterial pathogen, *Xanthomonas oryzae pv. oryzae.*

## Introduction

The coevolution between host plants and their pathogens can be best defined as a dynamic process involving reciprocal and adaptive genetic changes in the interacting species (Woolhouse et al., 2002). In the modern agriculture system, the relationships between crop plants and many of their pathogens are often represented by more extreme cases of arms-race, which are genetically governed by the classical gene-for-gene theory (Flor, 1971) and well explained by the benchmarked “zigzag model” of plant immune systems (Jones and Dangl, 2006). This model has since guided both basic and applied research on plant disease resistance. Considerable advances have been made in understanding the dynamics of direct or indirect interactions between plant resistance genes (R-genes) and pathogen virulence effector proteins (Hammond-Kosack and Jones, 1997; De Wit et al., 2009; Kapos et al., 2019). However, recent findings in diverse pathosystems are becoming increasingly difficult to fit into the simplified binary view of plant–pathogen interactions (Wu et al., 2017, 2018). An alternative and more inclusive “invasion model” (Cook et al., 2015) and a further refined “spatial invasion model” (Kanyuka and Rudd, 2019) of plant immunity have been proposed to be more general in defining the host–pathogen systems.

Over the past several years, rapid development of sequencing technologies and computational methods, and genome-wide association studies (GWAS) have been demonstrated to be a powerful and feasible strategy to detect natural variation underlying complex quantitative traits in crop plants (Huang and Han, 2014). Since then, GWAS has been applied to gene/QTL discovery in both host plants and pathogens (Genissel et al., 2017). The application of GWAS in identifying genomic regions associated with pathogenicity has been reported in bacterial and fungal pathogens (Bartoli and Roux, 2017; Sánchez-Vallet et al., 2018), including *Pseudomonas syringae* (Monteil et al., 2016), *Parastagonospora nodorum* (Gao et al., 2016), and *Zymoseptoria tritici* (Hartmann et al., 2017). Characterizing the molecular landscape of plant–pathogen intergenomic interactions can considerably enrich our knowledge on the coevolutionary processes and adaptive dynamics of microbial species in plant communities (Bartoli and Roux, 2017). Wang et al. (2018a) reported the first case of applying a joint GWAS to quantifying the interaction between *Arabidopsis thaliana* and its bacterial pathogen, *Xanthomonas arboricola*. However, the genetic architecture underlying complex genome-wide interactions between crop plants and their pathogens have rarely been characterized at a population level.

Rice bacterial blight (BB), caused by *Xanthomonas oryzae* pv. *oryzae* (*Xoo*), is the most devastating rice bacterial disease worldwide (Niño-Liu et al., 2006). As an excellent model for understanding host–pathogen interactions, the rice-*Xoo* pathosystem has been intensively investigated in past decades and is well-known for the high degree of differential interactions between rice varieties and *Xoo* races (Ji et al., 2018). *Xoo* attacks rice mainly by suppressing host immunity with its type-III secretion system (T3SS), which contains transcription activator-like (TAL) effectors and non-TAL effectors. During the *Xoo* invasion, TAL effectors are injected into rice cells through the T3SS, trigger specific defense reactions, and transcriptionally activate corresponding host genes by recognizing and binding to specific sequences in their promoters (Boch and Bonas, 2010). Non-TAL effectors such as *Xanthomonas* outer proteins (Xops) also play important roles in the modulation of signaling in rice defense responses through peptidoglycan-triggered mitogen-activated protein kinase (MAPK) activation (Mudgett, 2005). The same Xops may contribute differently to virulence in different genetic backgrounds of *Xoo* strains or rice varieties primarily in a quantitative manner (Ji et al., 2018).

The past decades also witnessed tremendous progress in developing and deploying resistant varieties for effective BB management in rice production. Major efforts have been taken to develop BB-resistant rice varieties in breeding programs worldwide (Zhai et al., 2002; Zhou et al., 2011), but rapid losses of resistance of rice varieties carrying single R-genes have been frequently reported (Vera Cruz et al., 2000). Thus, there are well-documented cases of the coadaptation and arms-race between rice and *Xoo*, particularly since the Green Revolution (Mew et al., 1992). Continuous searching in rice gene pools have identified 46 major *Xa*/*xa* genes and many quantitative resistance (QR) loci, each conferring high-level resistance or partial resistance to a specific set of *Xoo* races (Liu et al., 2014; Hutin et al., 2015; Kim et al., 2015; Zhang et al., 2015; Busungu et al., 2016; Kim, 2018; Kim and Reinke, 2019; Neelam et al., 2020; Chen et al., 2020). Genetically, different rice *Xa*-genes and QR-loci tend to interact strongly with one another against specific *Xoo* races (Li et al., 2001, 2006). At least 11 *Xa*/*xa* genes of diverse functions have been cloned, including eight *Xa*/*xa* genes (*Xa1*, *xa5*, *Xa10*, *xa13*, *Xa23*, *xa25*, *Xa27*, and *xa41*) each of which mediates resistance through their interactions with corresponding TAL effectors of *Xoo*. Three dominant *Xa*-genes (*Xa3*/*Xa26*, *Xa4*, and *Xa21*) each encoding a kinase protein are known to mediate high-level resistance to a specific set of *Xoo* races (Ji et al., 2018). The relationship between rice and *Xoo* appears to be largely governed by the gene-for-gene system, as well demonstrated by several studied interactions between *Xoo* TAL effectors and rice *Xa*-genes, including *Xa10*/*avrXa10* (Tian et al., 2014), *Xa27*/*avrXa27* (Gu et al., 2005), *Xa23*/*avrXa23* (Wang et al., 2015a), *Xa21/RaxX* (Pruitt et al., 2015; Luu et al., 2019), and *PthXo2*/*xa25* or *OsSWEET13* (Zhou et al., 2015). While the presence of complex epistasis among rice *Xa*-genes and QR-loci provided insights into complex genetic networks underlying the rice defensive system to *Xoo* (Li et al., 2006), many important questions regarding the interactions between rice and *Xoo* remain unanswered. In particular, how rice defensive system (*Xa*/*xa* genes and QR-loci) interact with *Xoo* virulence genes at the whole genomic and population levels remains poorly understood. Answers to this question are important for a deep understanding of the coadaptation of crop plants and their bacterial pathogens and for efficient deployment of the host defensive system for effective disease control.

Here, we performed whole-genome sequencing of 23 diverse *Xoo* strains and two large sets of rice accessions and evaluated the reactions of these rice accessions to the *Xoo* strains. Comprehensive analyses of this big data led us to reveal the complex genome-wide interactions that underlie the reciprocal adaptation between rice and *Xoo*, which shed important light on the coadaptation patterns and mechanistic relationships between crop plants and their bacterial pathogens.

## Results

### Phenotypic and genomic diversity of the *Xoo* strains

For the first set rice materials, analysis of variance (ANOVA) using the mean lesion lengths indicated that resistance differences among the 73 rice accessions, virulence differences among the 23 *Xoo* strains, and the interactions between rice accessions and *Xoo* strains were all highly significant, and explained 52.2%, 18.1%, and 27.5% of the variance, respectively (Supplemental Data Set S1). The difference between the two experimental replications was insignificant (*P *=* *0.855). The broad-sense heritability estimates were very high for all *Xoo* races, ranging from 0.871 for P6 to 0.979 for P9a (Supplemental Data Set S1).

**Figure 1:**
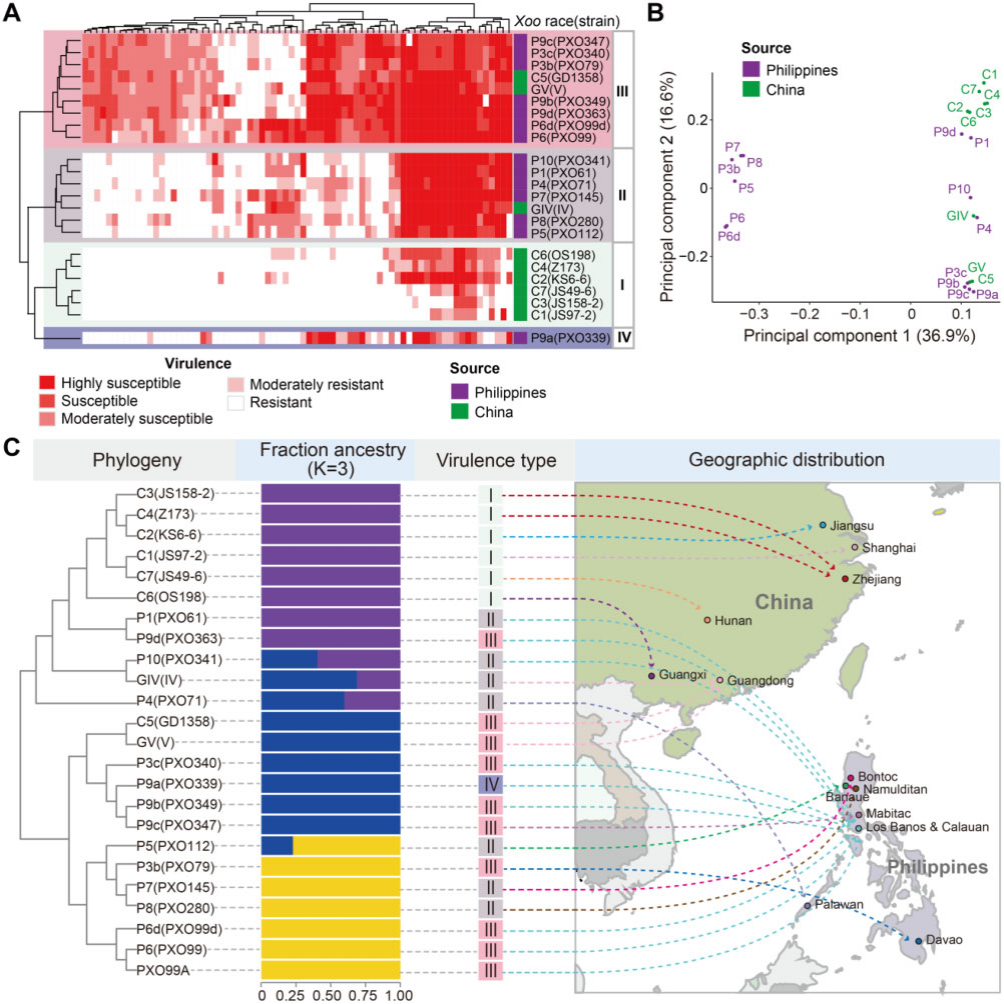
The origin of 23 diverse *Xanthomonas oryzae* pv. *oryzae* (*Xoo*) strains and their virulence. A, Reaction type of 73 rice accessions caused by 23 *Xoo* strains from China and the Philippines and classification of the 23 *Xoo* strains into four major groups based on their virulence levels (mean lesion length [LL]): resistant (LL <3 cm), moderately resistant (3 cm ≤ LL <5 cm), moderately susceptible (5 cm ≤ LL < 10 cm), susceptible (10 cm ≤ LL < 15 cm), and highly susceptible (LL ≥ 15 cm). B, Principal component analysis plots for the first two principal components of the 23 *Xoo* genomes. C, The population structure and geographic distribution of the 23 *Xoo* strains. The neighbor-joining tree was constructed from LD pruned SNPs. Fraction ancestry was calculated with STRUCTURE software using an ancestry number of 3, which is shown in different colors

Based on the lesion lengths of 73 diverse rice accessions (Supplemental Data Sets S1 and S2), the 23 *Xoo* strains were classified into four virulence groups (Figure 1A). Group I included six weak virulence (WV) races (C1, C2, C3, C4, C6, and C7) from China. Group II contained seven moderate virulence (MV) races (GIV from China, and P1, P4, P5, P7, P8, P10 from Philippines). Group III included nine strong virulence (SV) races (C5, GV from China, and P3b, P3c, P6, P6d, P9b, P9c, P9d from Philippines). Group IV contained a single race, P9a from Philippines, which showed a unique pathogenicity pattern in the tested rice accessions.

Whole-genome sequencing of the 23 *Xoo* strains using next-generation sequencing technology produced a total of 19.5 GB high-quality bases with an average sequencing depth of 172.9 ± 4.3x for each strain (Supplemental Data Set S3). Comparing sequenced genomes with the PXO99^A^ reference genome (Salzberg et al., 2008) identified 33,006 single-nucleotide polymorphisms (SNPs), including 13,053 nonsynonymous SNPs, 11,113 synonymous SNPs, and 8,840 SNPs in intergenic regions. Remarkably, 50% of genic SNPs occurred in approximately 300 highly variable genes. Principal component analysis (Figure 1B) and population structure analysis (Figure 1C) resolved all 23 *Xoo* strains into four major clusters. Six Chinese races (C1–C4, C6, and C7) and two Philippine races (P1 and P9d) formed a single cluster. Four Philippine races (P3c, P9a, P9b, and P9c) plus two Chinese races (GV and C5) formed the second one. Six Philippine races (P3b, P5, P7, P8, P6, and P6d) constituted the third cluster. The remaining three races (Philippine races P4 and P10, and Chinese race GIV) were admixtures (Figure 1C). We noted that the *Xoo* race structure (Figure 1, B and C) depicted by the SNP data was very similar to the grouping by the phenotypic data (Figure 1A) except for the three tropical *Xoo* race groups each containing several highly related MV (II) and SV (III) races. A relatively high number (22) of multi-locus genotypes (MLGs) were found out of the 23 strains, indicating a low clonality of the sampled *Xoo* population.

We found that recombination along the *Xoo* genomes occurred in a nonuniform manner with a recombination hotspot in the region of 1.57–1.86 Mb of the *Xoo* genome where genes related to chemotaxis and two-component systems are highly enriched (Supplemental Figure S1A). Notably, recombination occurred more frequently in genomes of SV or MV races P6, P6d, P5, P7, P8, and P3b with approximately 900 recombination events. In contrast, recombination occurred much less frequently in genomes of WV races C3, C4, C2, C1, C7, and C6. Thus, elevated recombination in the SV and MV races may have directly contributed to virulence shifts of SV and MV *Xoo* races in the tropics.

To examine the effect of the recombination hotspot on the genome-wide linkage disequilibrium (LD) decay distance, we compared the LD decay curves across the *Xoo* genomes with and without the recombination hotspot (Supplemental Figure S1B). Clearly, similar LD decay curves were observed, but the observed average LD block (*r*^2^ decays to 0.25) with the recombination hotspot (1.57–1.86 Mb) was ∼2.1 kb, smaller than the average LD (∼2.4 kb) without the recombination hotspot. Because higher recombination rates cause faster LD decay which ultimately results in higher mapping resolution, the high level of genomic diversity and small LD decay values indicated that the *Xoo* population was suitable for further identification of virulence genes by GWAS.

### Identification of genes related to *Xoo* virulence

We first examined SNPs in 48 known virulence-related genes (Supplemental Table S1) and found that ten TAL effectors and two T3SS genes (*hrpF* and *hpaF*) showed significantly higher SNP densities than the genome-wide background of 0.0058, indicating that these genes had undergone fast functional diversification. Three TAL effectors (*talC8b*, *talC8a*, and *talC7A*) and two T3SS genes (*hrpA* and *hrcC*) showed significantly lower SNP densities, implying that these genes are essential for the fitness of *Xoo* and thus had gone through strong purifying selection.

To identify additional virulence-related genes, we associated genomic variants of the 23 *Xoo* strains with the mean lesion lengths caused by inoculation with these strains on 73 diverse rice accessions. To overcome the small sample size and take advantage of multiple phenotypes, we utilized a combined association score (CAS) that combined independent association results (see “Methods” section for details). As a result, 86 significant SNPs in 47 *Xoo* genes were detected (Supplemental Table S2), 5 of which (*PXO_00274*, *PXO_00272*, *pthXo1*, *PXO_00124*, and *PXO_00502*) are located within the 290-kb recombination hotspot. We noted that the small difference in LD block sizes within and outside the recombination hotspot had little impact on the number and resolution of the detected virulence-related genes (Fisher’s exact test, *P *=* *0.119), though we indeed observed a higher power (more significant SNPs in the recombination hotspot, Fisher’s exact test *P *=* *0.009). Permutation analysis suggested that the probability of more than 86 significant SNPs in a random GWAS is less than 0.0001. This suggested a low false discovery rate of 0.17, or on average, 71 out of the 86 SNPs were true signals.

The 47 genes contained 5 of the 48 known virulence-related genes previously reported in *Xoo* (a T3SS gene *hrpF* and four TALs, *pthXo1*, *pthXo7*, *talC5a*, and *talC9b*), and 18 highly likely virulence-related genes based on the literature on other related bacterial species, including four type-VI secretion system (T6SS) genes, two TonB-dependent receptors (TBDRs), and a chorismate mutase (Supplemental Table S3). This report thus implicates four T6SS genes (*PXO*_*03644*, *PXO_04700*, *PXO*_*04712*, and *PXO*_*00502*) and TBDRs (*PXO_01644* and *PXO*_03467) in the virulence of *Xoo* to rice. T6SS functions as a contractile nanomachine to puncture target cells and deliver effectors highly associated with the virulence of plant and animal pathogens (Alteri and Mobley, 2016). TBDRs were reported to play important roles in *Xoo* infection by obtaining the required iron from host iron-carrying proteins (Grinter et al., 2016). The chorismate mutase was experimentally validated to affect the pathogenicity of *Xoo* XKK.12 (Degrassi et al., 2010). The remaining 11 significantly associated genes are linked to known virulence-related genes (Supplemental Table S3).

### Selective signatures on virulence-related genes in *Xoo*

To determine which of these *Xoo* virulence-related genes were the targets of selection from rice, we computed nucleotide diversity (*π*) across the *Xoo* genome (Supplemental Figure S1C). Divergent patterns of nucleotide diversity across the *Xoo* genome differentiated the WV races, MV races, and SV races, indicating that the evolution of these *Xoo* races from WV to MV and SV resulted from genome-wide positive selections for functional diversification of many virulence-related genes. Twenty-three of the 47 detected virulence-related genes had significantly higher SNP densities than the genome-wide background genes in *Xoo* (Supplemental Table S2). Of these, six important ones were noted. The first was *hrpF* (*PXO_03417*), a T3SS gene with a high SNP density of 0.0203 (23 nonsynonymous SNPs). *hrpF* was under strong positive selection for functional diversification in all *Xoo* races except for races P1 and P9d (Supplemental Figure S2A). There are two major haplotypes at *hrpF* among the *Xoo* races (Supplemental Figure S2B). Hap1 was strongly associated with increased virulence and mainly present in one branch of the SV *Xoo* races (Supplemental Figure S2C). The second gene was a TBDR (*PXO_01644*), which showed an extremely high SNP density of 0.116 (96 nonsynonymous SNPs) and exhibited distinct haplotypes among the four *Xoo* virulence groups (Supplemental Figure S3, B and C). Interestingly, one of the two nonsynonymous SNPs causing a substitution from Glu to Lys in the domain loop of this protein showed the strongest associations with strong virulence (Supplemental Figure S3, D–F). In WV races, this particular SNP was very likely to cause reduced ability of the TBDR in binding specific iron-carrying rice proteins required for *Xoo* infection (Grinter et al., 2016; Supplemental Figure S3G). This *TBDR* gene (*PXO_01644*) was under very strong positive selection for functional diversification in all WV races and some of the SV and MV races (Supplemental Figure S3A). The remaining four were T6SS genes showing high SNP densities (Supplemental Table S2), implying that they were the key virulence genes responding to selection from the host.

### Differentiated responses of 701 rice accessions to four representative *Xoo* races

**Figure 2:**
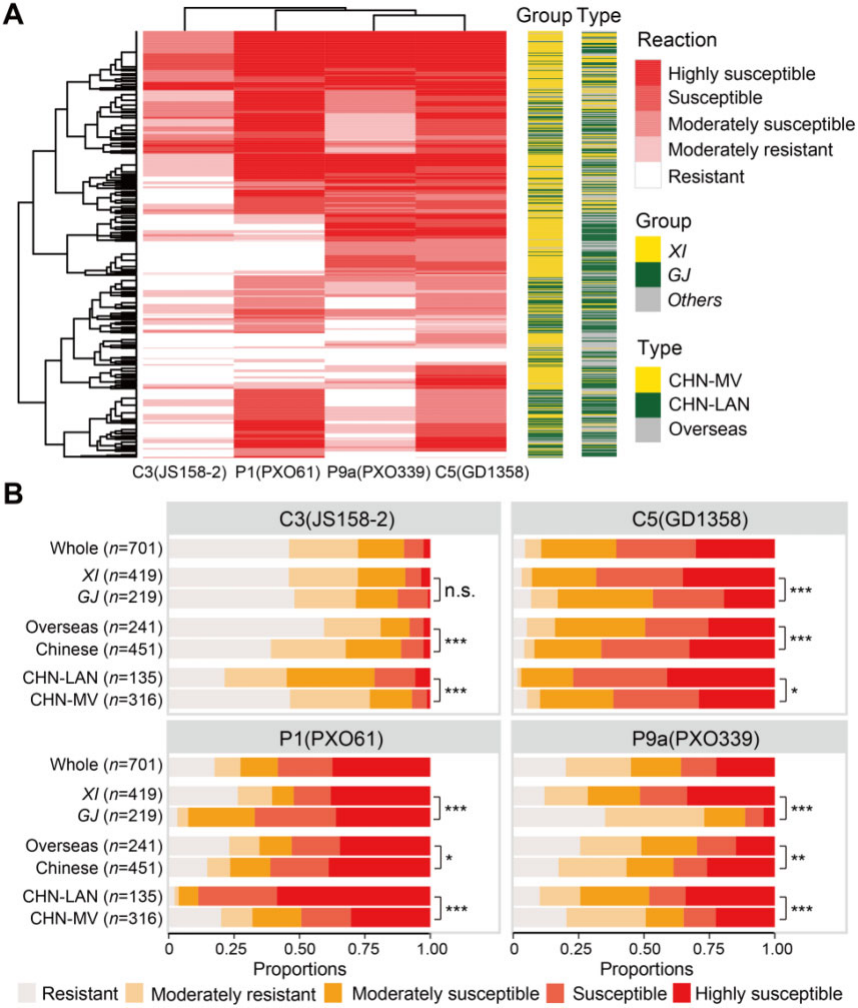
Summary of the resistance reactions of 701 rice accessions to four representative *Xoo* races (C3, C5, P1, and P9a). A, The heatmap of reactions of 701 rice accessions to four representative *Xoo* races (C3, C5, P1, and P9a). *XI*, *Xian*/*indica*; *GJ*, *Geng*/*japonica* B, Proportions of different reactions in rice subpopulations to the four *Xoo* races, in which Pearson’s Chi-squared tests were performed with “***” representing *P* < 0.001 and “NS” representing no significant difference

To better characterize the interaction between rice and *Xoo*, we selected four races representing the four major *Xoo* virulence groups, based on the results shown in Figure 1: C3 (WV or I), P1 (MV or II), C5 (SV or III), and P9a (IV), and used these to inoculate 701 second-set rice accessions of diverse origins (Figure 2A; Supplemental Data Set S4). ANOVA indicated that resistance differences among the 701 rice accessions, virulence differences among the four *Xoo* races, the interactions between rice accessions and *Xoo* races were all highly significant and explained 46.1%, 17.4%, and 30.2% of the total phenotypic variation in mean lesion lengths of the second set rice materials, respectively (Supplemental Data Set S4). The difference between the two experimental replications was insignificant (*P *=* *0.236). The broad-sense heritability estimates were 0.797, 0.811, 0.857, and 0.880 for *Xoo* races C5, C3, P1, and P9a, respectively. Population structure analyses from LD pruned SNPs classified the 701 rice accessions into two main well-known subspecies, *Xian* (*indica*) and *Geng* (*japonica*; Supplemental Figure S4, A, B, and D). The LD (*r*^2^) dropped to half of its maximum value at ∼300 kb in the whole population (Supplemental Figure S4C).

To evaluate the specific impact of population structure, geographical origins, and variety types of rice accessions, the 701 accessions were divided into six panel subpopulations, including *Xian* (419 accessions), *Geng* (219 accessions), Chinese (451 accessions), overseas (241 accessions), and two subpopulations of Chinese accessions, including 135 Chinese landraces (CHN-LAN) and 316 Chinese modern varieties (CHN-MV), with 158 accessions shared between subpopulations Chinese and *Geng*, 266 accessions shared between subpopulations Chinese and *Xian*, 60 accessions shared between subpopulations Overseas and *Geng*, 145 accessions shared between subpopulations Overseas and *Xian* (Supplemental Data Set S4). The proportion of the 701 accessions showing high level (lesion length <3 cm) resistance was highest (42.9%) against WV race C3, followed by P9a (19.5%), P1 (17.1%), and C5 (4.3%; Figure 2). Rice accessions of the whole and specific panel populations showed considerable variation and race-specificities in their resistances to the four *Xoo* races (Figure 2B). In general, more *Geng* accessions were resistant to C5 and P9a than *Xian* accessions, and the opposite was true when against P1, indicating a major difference between the two rice subspecies in their resistance to *Xoo* races. The proportion of resistant accessions in CHN-LAN was 19.3%, 2.2%, 9.6%, and 1.5% against *Xoo* races C3 (WV or I), P1 (MV or II), P9a (IV), and C5 (SV or III), which was much lower than those (43.7%, 19.6%, 19.9%, and 5.1%) of CHN-MV (Figure 2B). Clearly, CHN-MV had much stronger resistance against all four *Xoo* races, as a result of modern breeding for improving BB resistance in China.

### Identification of rice QR genes and their race specificities

**Figure 3:**
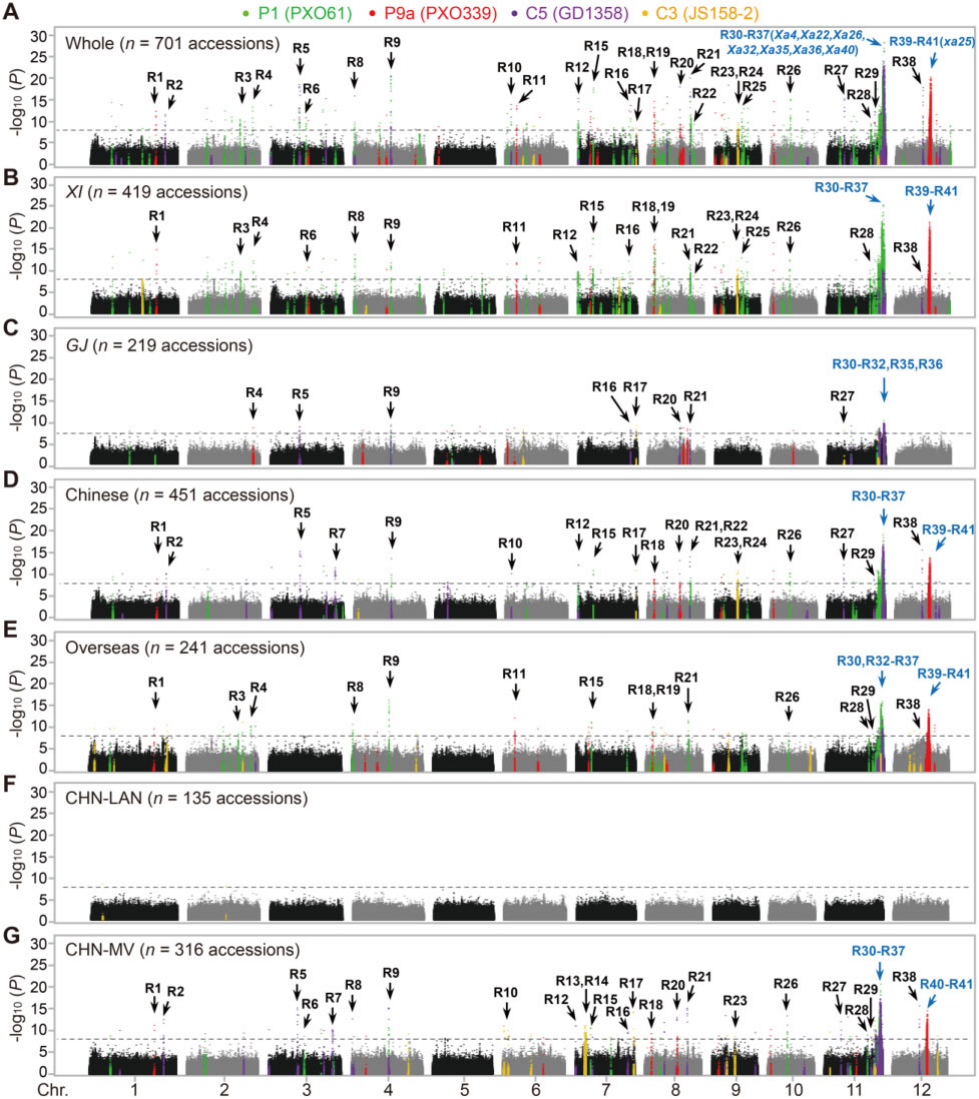
The Manhattan plots of genome-wide associations with bacterial blight resistance to four representative *Xoo* races in seven panels. A, Whole panel. B, *XI* panel. C, *GJ* panel. D, Chinese panel. E, Overseas panel. F, CHN-LAN panel. G, CHN-MV panel. Gold, violet, green and red points indicate significant SNPs associated with resistance to *Xoo* races C3, C5, P1, and P9a, respectively. Horizontal dashed lines in the Manhattan plots indicate their respective genome-wide significance thresholds (*P*-values adjusted by Bonferroni correction): of 3.02 × 10^−8^, 2.55 × 10^−8^, 6.81 × 10^−8^, 3.48 × 10^−8^, 2.63 × 10^−8^, 3.17 × 10^−8^, and 4.23 × 10^−8^. Arrows point to the genomic regions listed in Supplemental Table S4. *XI*, *Xian*/*indica*; *GJ*, *Geng*/*japonica*

GWAS using data of the whole population and six panel subpopulations (*Xian*, *Geng*, Chinese, overseas, CHN-LAN and CHN-MV) detected 5,432 significant SNPs associated with QR to one or two *Xoo* races (Supplemental Data Set S5), which are mainly clustered in 41 genomic regions (R1–R41) < 300 kb (Figure 3; Supplemental Figure S5 and Supplemental Table S4). Of the 5,432 SNPs, 2,703 were computationally predicted in 318 QR-genes and the remaining ones were located in 372 intergenic regions based on the Nipponbare reference genome annotation (MSU v7; Supplemental Data Set S5). The detected QR-genes mainly encode proteins with nucleotide-binding adaptor shared by APAF-1, R proteins, and CED-4 (NB-ARC) domains and leucine-rich repeats (NLR; Supplemental Figure S6E) and were enriched in biological processes related to plant responses to pathogens, including apoptosis, programmed cell death, defense response, and protein amino acid phosphorylation (Supplemental Figure S6D). Interestingly, 827 (15.2%) of the significant SNPs are located in retrotransposon or transposon genes (Supplemental Data Set S5).

The identified SNPs (genes) for resistance showed strong race-specificity with 4,847 (89.2%) of the detected SNPs and 272 (85.5%) of the detected genes associated with resistance to a single *Xoo* race and almost all the remaining SNPs and genes associated with resistance to both C5 and P1 (Supplemental Figure S6, A–C). The strong race specificity of the detected SNPs was primarily reflected in the *Xian-Geng* differentiation as a result of the adaptation of *Xoo* to rice. Specifically, of the 46 SNPs (10 genes) for resistance to WV race C3 detected in *Xian* or *Geng* subpopulations, 16 SNPs (3 genes) and 30 (7 genes) were detectable only in *Geng* and *Xian* accessions, respectively (Supplemental Data Set S5). Similarly, of the 1,471 SNPs (44 genes) for resistance to P9a in *Xian* or *Geng* subpopulations, 1,454 were detectable only in *Xian* accessions and the remaining 17 only in *Geng* accessions. Of the 1,983 SNPs (167 genes) for resistance to MV race P1 in *Xian* or *Geng* subpopulations, 1,878 were detected only in *Xian* accessions and 105 only in *Geng* accessions. Of the 259 SNPs (24 genes) for resistance to SV race C5 in *Xian* or *Geng* subpopulations, 49 were detected only in *Xian* accessions, 159 only in *Geng* accessions, and the remaining 51 in both *Xian* and *Geng*.

We also performed two-way ANOVA to examine the interaction effects between SNP and subspecific populations using 3,656 subpopulation-specific SNPs. Of these, 414 (11.3%) SNPs were fixed in one of the subpopulations. For the remaining 3,242 SNPs, 2,482 (76.6%) SNPs were detected with significant interaction effects between SNPs and subpopulations, indicating that these SNPs have different effects between the two subpopulations (Supplemental Data Set S5). Taking these results together, of the 228 detected rice genes for resistance to the four *Xoo* races in *Xian* or *Geng* subpopulations, 213 (93.4%) were associated either *Xian* or *Geng* with only 15 (6.6%) shared with both. We noted that 5, 257, 965, and 572 SNPs for resistance to C3, P9a, P1, and C5 detectable only in the whole population but neither in *Xian* nor in *Geng* also resulted from the *Xian*-*Geng* differentiation (Supplemental Data Set S5).

The high resolution of our GWAS resolved several of the detected QR-genes into a few or single candidate genes with clear resistance haplotypes. The first was *xa25* (*LOC_Os12g29220*) on chromosome 12, which harbors a group of SNPs with the strongest signals for resistance to P9a. As a cloned recessive R-gene encoding a sucrose transporter (*OsSWEET13*; Liu et al., 2011), three major haplotypes were detected at *xa25* (Supplemental Figure S7A). Hap3 was associated with susceptibility to P9a and had a frequency of 13.8% in *Xian* but absent in *Geng*, while Hap1 and Hap2 were associated with resistance had significantly higher frequencies in *Geng* (58.4% and 0.5%) than in *Xian* (7.4% and 11.9%). This explained the different responses of *Xian* and *Geng* accessions to P9a (Supplemental Figure S7, B and C). The second candidate was *Xa26* (*LOC_Os11g47210*), which encodes a receptor kinase-like protein (Sun et al., 2004). In the intragenic region of *Xa26*, we detected five significant signals for resistance to C5 and four significant signals for resistance to P1. However, we did not observe any resistance-related haplotypes. Alternatively, much stronger signals with 38 highly significant SNPs were detected for resistance to P1 in its neighboring locus within the *Xa26* gene family cluster (Li et al., 2012a), which (*LOC_Os11g47240*) encodes a leucine-rich-repeat receptor kinase EXS precursor (Supplemental Data Set S5). Sixteen significant nonsynonymous SNPs together with a SNP that produced a stop codon revealed three major haplotypes at *LOC_Os11g47240* (Supplemental Figure S7D). Hap1 and Hap2 were associated with susceptibility to P1 and had significantly lower frequencies (23.2% and 19.1%) in *Xian* than in *Geng* (50.2% and 25.1%), while Hap3 was associated with resistance with a frequency of 15.5% in *Xian* but absent in *Geng* (Supplemental Figure S7, D and E). Meanwhile, Hap3 was more abundant in CHN-MV than in CHN-LAN, showing strong *Xian*-*Geng* differentiation and strong selective signature during breeding (Li et al., 2012a). Specifically, the kinase domains in exons 2 and 3 of *LOC_Os11g47240* appeared to be more important for its resistance function since two nonsynonymous SNPs and the SNP that produced a stop codon in its kinase domains showed the strongest associations with resistance. These results indicated that *LOC_Os11g47240* is an important member of the *Xa26* gene family cluster conferring BB resistance.

**Figure 4:**
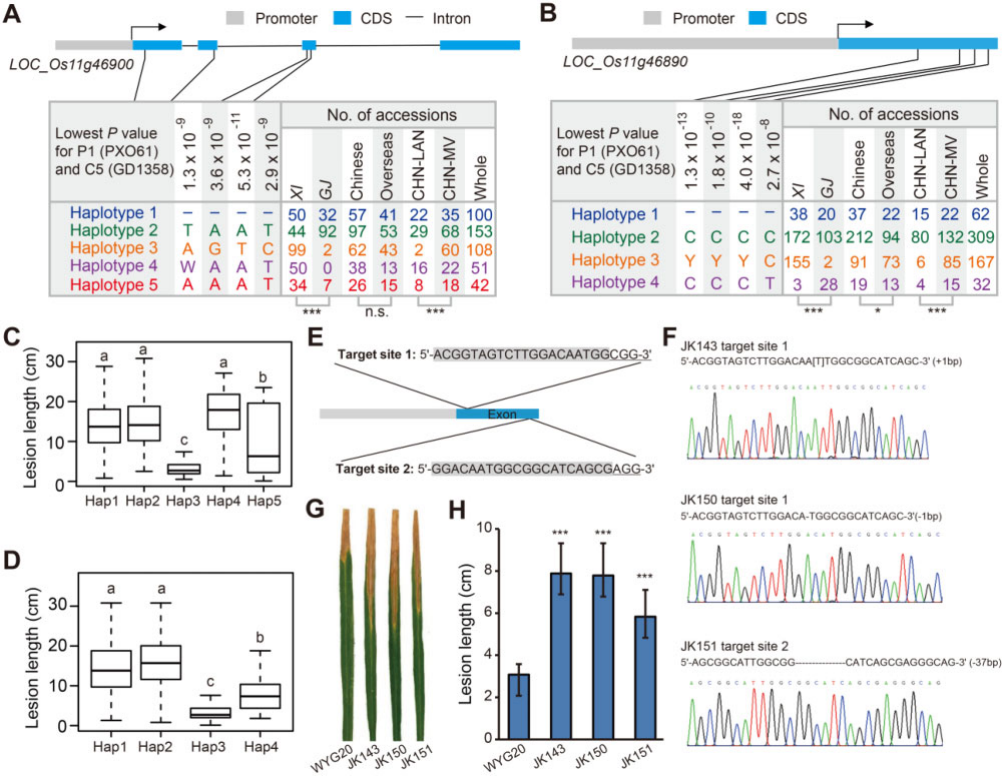
Analysis of the candidate gene of *Xa40*. A, B, Haplotype analyses of *LOC_Os11g46900* (A) and *LOC_Os11g46890* (B). Both used four significant nonsynonymous SNPs in the coding regions based on the MSU v7 annotation of Nipponbare reference genome. Haplotypes with less than five accessions were not shown. Chi-square tests were used to compare haplotype frequencies among subpopulations, with “***” representing *P* < 0.001 and “NS” representing no significant difference. “–“ mean missing reads. The characters “W” and “Y” represent A/T and C/T heterozygous alleles. *XI*, *Xian*/*indica*; *GJ*, *Geng*/*japonica* C, D, Comparisons of the mean lesion lengths (caused by *Xoo* race P1 [PXO61]) vs. haplotypes of *LOC_Os11g46900* (C) and *LOC_Os11g46890* (D) in subpopulations. One-way ANOVA followed by Tukey’s honest significant difference (HSD) post hoc tests were used to detect the major effects. Different letters on the boxplots indicate statistically significant differences at *P* < 0.001 by Tukey’s HSD post hoc tests. Boxplots represent the interquartile range, the thick line in the middle of each box represents the median, and the whiskers represent 1.5 times the interquartile range. The data are based on two replications. The box in each column in (C) and (D) corresponds to the same haplotype (Hap) in (A) and (B), respectively. E, The CRISPR/Cas9 target sites of *LOC_Os11g46890* in Wuyugeng20 (WYG20). The target sequence is marked in grey background and PAM motif (NGG) is marked by underline. F, Sequence at the target site of three T_1_ mutants (JK143, JK150, and JK151) of *LOC_Os11g46890*. G–H, Reaction (G) and the lesion lengths (H) of wild-type WYG20 and three knockout T_1_ plants inoculated with *Xoo* race C5 (GD1358). Data are presented as mean ± sd (*n* = 9). Asterisks indicate a significant difference compared with WYG20, with ***P < 0.001 by Student’s two-sided *t* test

Another example is *Xa40*, a reported dominant R-gene conferring high-level resistance to all Korean *Xoo* races, which was fine-mapped within a ∼80-kb region on chromosome 11 (Kim et al., 2015). A total of eight candidate genes were functionally predicted in the target region, including a WAK3 gene (*LOC_Os11g46900*) and a protein-coding gene of unknown function (*LOC_Os11g46890*) with gradually increased levels of expression over time after *Xoo* inoculation. We detected five haplotypes for *LOC_Os11g46900* (*P *=* *5.3 × 10^−11^ of the lead SNP; Figure 4A). Hap3 and Hap5 were associated with resistance to P1 and had significantly higher frequencies (23.6% and 8.1%) in *Xian* than in *Geng* (0.9% and 3.2%), while Hap1, Hap2, and Hap4 were associated with susceptibility with differentiated frequencies of in *Xian* (11.9%, 10.5%, and 11.9%), and *Geng* (14.6%, 42.0%, and 0%; Figure 4, A and C). Interestingly, its neighboring gene, *LOC_Os11g46890* contains more significant SNPs (*P *=* *4.0 × 10^−18^ of the lead SNP) for resistance to C5 and P1 with four haplotypes (Figure 4B). Hap3 and Hap4 were associated with resistance to P1 and had significantly higher frequencies (37.0% and 0.7%) in *Xian* than in *Geng* (0.9% and 12.8%), while Hap1 and Hap2 were associated with susceptibility with high frequencies in both *Xian* (9.1% and 41.1%) and *Geng* (9.1% and 47.0%; Figure 4, B and D).

We further validated the function of *LOC_Os11g46890* on BB resistance by knockout in a *Geng* variety Wuyugeng20, which carries the major resistance haplotype Hap4. The mutants of Wuyugeng20 were generated from two CRISPR/Cas9 targeting sites of *LOC_Os11g46890* using the pYLCRISPR/Cas9Pubi-H system (Figure 4, E and F). Three independent homozygous T_1_ mutants with different types of mutation in the coding region were obtained. Among them, line JK143 (1-bp insertion in target site 1) caused a stop codon and produced a truncated protein, while the other two lines (JK150 and JK151) caused frame shifts and encoded putative new proteins (Supplemental Figure S8). Analysis of these T_1_ plants containing homozygous mutations within *LOC_Os11g46890* indicated that resistance of the mutants to C5 was significantly lower compared to the wild-type (Figures 4, G and H), suggesting that *LOC_Os11g46890* is more likely to be the true candidate for *Xa40*, though the possibility that *LOC_Os11g46900* might also contribute to resistance jointly with *LOC_Os11g46890* could not be excluded.

We identified a protein-coding gene with unknown function (*LOC_Os11g46250*) as the candidate for *Xa22* (Wang et al., 2003), within which 152 significant SNPs cluster in its promoter and genic region showing the strongest associations with resistance to races P1 (*P *=* *2.0 × 10^−14^) and C5 (*P *=* *6.7 × 10^−23^). Interestingly, we observed differentiated resistance of different alleles at *LOC_Os11g46250* against different *Xoo* races. We discovered three major haplotypes at this locus based on 58 significant SNPs in its 1-kb upstream promoter region, 5′- untranslated region (UTR), and nonsynonymous SNPs in the coding region (Supplemental Figure S9A). Hap2 was associated with resistance against MV race P1 (Supplemental Figure S9B), but with susceptibility to SV race C5 (Supplemental Figure S9C). Hap2 had frequencies of 18.4% and 0% in populations *Xian* and *Geng*, respectively (Supplemental Figure S9A). In contrast, Hap3 was associated with moderate susceptibility to P1 but with moderate resistance against C5, and had low frequencies of 2.4% and 5.0% in *Xian* and *Geng*, respectively. These results suggest that *LOC_Os11g46250* encoding an expressed protein of unknown function is the most likely candidate for *Xa22*.

To identify which loci contribute to high-level resistance to the SV race (C5), we examined the graphical genotypes of the 30 accessions resistant to C5 for all loci associated with resistance to C5 (Supplemental Figure S10). Interestingly, we could not attribute the high level of resistance of the 30 accessions to C5 to any single locus. However, the coexistence of the resistance alleles at eight loci appeared to be responsible for the resistance of 19 accessions to C5, suggesting the eight loci jointly determined the resistance to C5, while the resistance of the remaining 11 accessions to C5 appeared to under different genetic control.

### Selective signatures at the detected QR-loci in rice from modern breeding

**Figure 5:**
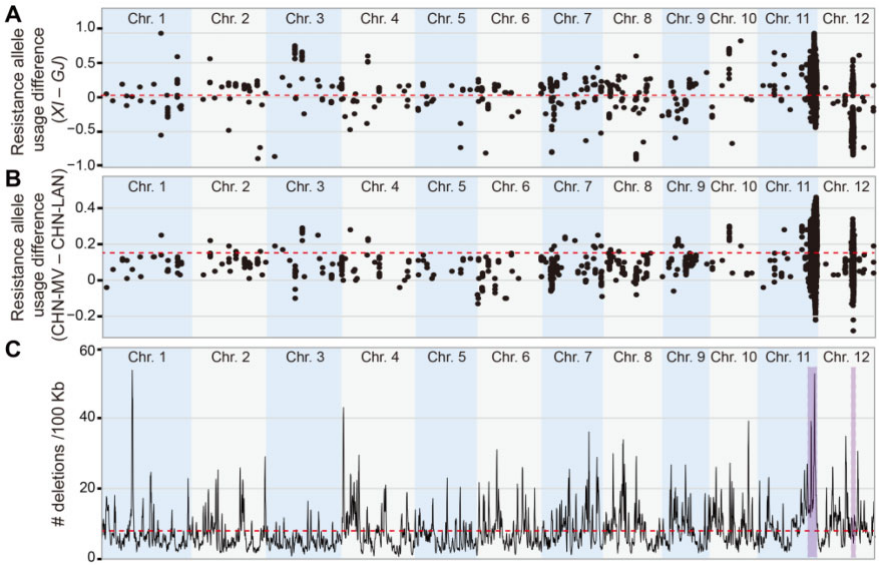
Selection and evolution of the rice QR-genes. A, Comparison of resistance allele usage between *Xian* accessions (*n* = 419) and *Geng* accessions (*n* = 219) for each SNP associated with resistance to *Xoo*. *XI*, *Xian*/*indica*; *GJ*, *Geng*/*japonica*. B, Comparison of resistance allele usage between CHN-MV (*n* = 316) and CHN-LAN (*n* = 135) for each SNP associated with resistance to *Xoo*. The dotted red lines in (A) and (B) indicate the mean frequency difference of all significant SNPs detected in rice GWAS panel. The resistance allele was determined by its corresponding phenotypes in the whole population. C, Density of large-sized deletions (>100 bp) calculated by a sliding window of 500 kb with each moving step of 100 kb. The density was further adjusted to deletion numbers per 100 kb. The two important regions (R30–R37 and R40–R41) were highlighted. The dotted red line indicates the genome-wide mean value

To determine the overall impact of modern breeding on QR-loci across the rice genome, we examined the genome-wide frequency shifts of resistance alleles (defined as the alleles at detected SNPs with the shortest mean lesion length compared with the other alleles) at all significant SNPs for resistance to the four *Xoo* races both in the whole and in the six rice panel populations. *Xian* and *Geng* apparently achieved resistance by using different alleles at most detected QR-loci, though no global resistance allele frequency shifts were observed between *Xian* and *Geng* (Figure 5A; Supplemental Figure S11A). When comparing CHN-MV with CHN-LAN, we observed an apparent genome-wide frequency increase in resistance alleles by 0.15 (Figure 5B; Supplemental Figure S11B). Among them, 3,735 (68.8%) of the 5,432 significant SNPs showed significant frequency shifts for resistance alleles, resulting presumably from the artificial selection for improving resistance via modern breeding. Notably, 55% of the significant SNPs and 44% of nontransposon genes locate in the ∼3.3 Mb region of R30–R37 on chromosome 11, where seven fine-mapped (*Xa22*, *Xa32*, *Xa35*, *Xa36*, and *Xa40*)/cloned (*Xa4* and *Xa26*) *Xa*-genes reportedly reside. R40–R41 (∼0.5 Mb) on chromosome 12 contains an additional 30.8% (11%) of significant SNPs (genes) detected.

**Figure 6:**
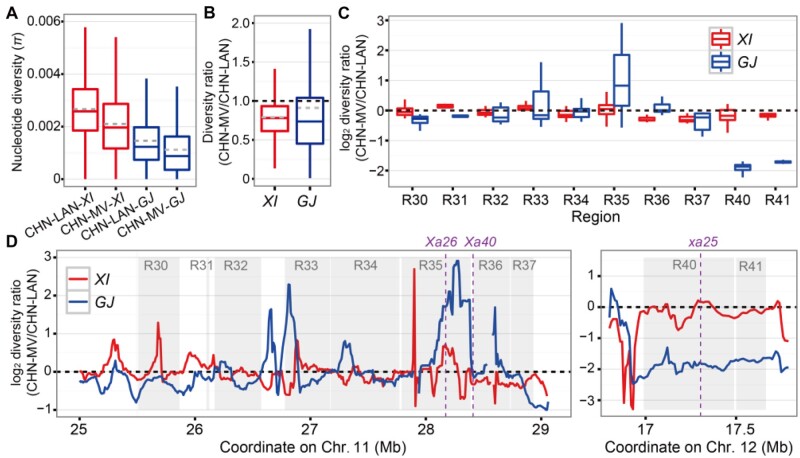
Impact of artificial selection on the nucleotide diversity of QR-genes. A, Boxplot of the global nucleotide diversity of landraces and modern varieties. CHN-LAN-*XI*, Chinese *Xian*/*indica* landraces (*n* = 78 accessions); CHN-MV-*XI*, Chinese *Xian*/*indica* modern varieties (*n* = 188 accessions); CHN-LAN-*GJ*, Chinese *Geng*/*japonica* landraces (*n* = 51 accessions); CHN-MV-*GJ*, Chinese *Geng*/*japonica* modern varieties (*n* = 107 accessions). B, Boxplot of the nucleotide diversity ratio, defined as diversity of CHN-MV divided by that of CHN-LAN. *XI*, *Xian*/*indica*; *GJ*, *Geng*/*japonica*. C, Boxplot of the nucleotide diversity ratio (log2-transformed) of the important QR-regions. The red boxplot shows the diversity ratio of CHN-MV-*XI* and CHN-LAN-*XI* and the blue boxplot shows the diversity ratio of CHN-MV-*GJ* and CHN-LAN-*GJ* in (B) and (C). D, Nucleotide diversity ratio across R30–R37 and R40–R41. The red line shows the log2-transformed diversity ratio of CHN-MV-*XI* and CHN-LAN-*XI* and the blue line shows the log2-transformed diversity ratio of CHN-MV-*GJ* and CHN-LAN-*GJ*. Coordinates of known *Xa*/*xa* genes are displayed with purple dashed vertical lines. Boxplots represent the interquartile range, the thick line in the middle of each box represents the median, the whiskers represent 1.5 times the interquartile range, and the dashed grey lines show the mean value

Compared with CHN-LAN, CHN-MV displayed greatly decreased genome-wide nucleotide diversity (Figure 6, A and B; Supplemental Figure S11C). However, R40–R41 for resistance to P9a were apparently under strong directional selection in the *Geng* population, while regions R30–R37 were characterized with high-level diversity (Figure 6, C and D). Using the RPAN database (Sun et al., 2017), we found that 67.5% of the genes detected in regions R30–R37 were distributed/dispensable. These identified genes showed 2.2-fold enrichment in comparison to the genome background with a distributed gene rate of 0.30 (the hypergeometric test, *P *=* *1.7 × 10^−20^). In particular, genes in R30–R37 tended to have significantly higher gene copy number variations (CNVs) when the *Xian* Minghui63/Zhenshan97 genomes (Zhang et al., 2016) were compared with the *Geng* Nippobare genome (Supplemental Figure S11D). This is consistent with the reported presence of many tandemly arrayed R-genes in this region (Rizzon et al., 2006). An additional peak of large deletions (>100 bp) was detected in R30–R37 among the 701 genomes (Figure 5C), suggesting high frequencies of homologous recombination in this region. All these results indicate that R-genes in this region evolved quickly, resulting primarily from the unique nature of this region harboring high numbers of SNPs and CNVs.

To gain insights into the question of whether deployment of single major *Xa*/*xa* genes were the driving force shaping the *Xoo* race structure, we examined the frequency distribution of major functional haplotypes at four large-effect QR-genes located in the two genomic regions of chromosomes 11 and 12 where the highest frequency shifts from breeding were observed. At *xa25*, differential shifts were observed for the resistance haplotypes Hap1 and Hap2. When CHN-MV was compared with CHN-LAN, Hap1 decreased by 1.9% and Hap2 (predominant in *Xian* but rare in *Geng*) decreased by 2.3% (Supplemental Figure S7A). At *Xa26* homolog (*LOC_Os11g47240*), the resistance Hap3 had a significantly higher frequency in CHN-MV (7.0%) than in CHN-LAN (1.5%; Supplemental Figure S7D). For the *Xa40* candidate (*LOC_Os11g46890*), the resistance Hap3 and Hap4 had significantly higher frequencies (26.9% and 4.7%) in CHN-MV than in CHN-LAN (4.4% and 3.0%; Figure 4B). For the *Xa22* candidate (*LOC_Os11g46250*), the resistance Hap2 against MV race P1 had a significantly higher frequency (16.1%) in CHN-MV than in CHN-LAN (1.5%; Supplemental Figure S9, A and B). When against SV race C5, resistance Hap3 at *Xa22* was associated with moderate resistance with a significantly higher frequency (3.2%) in CHN-MV than in CHN-LAN (1.5%; Supplemental Figure S9, A and C). These results plus those in the previous section led us to the conclusion that artificial selection of modern breeding acting on the major *Xa* genes locate in the ∼3.3 Mb region of R30–R37 were the primary driving force shaping the observed *Xoo* race structure (Figure 1), consistent with the extensive deployment of *Xa4* in breeding programs during 1970s–1990s at IRRI and China (Mew et al., 1992; Zhang, 2009).

### Cross-species genome-genome interactions between *Xoo* and rice

**Figure 7:**
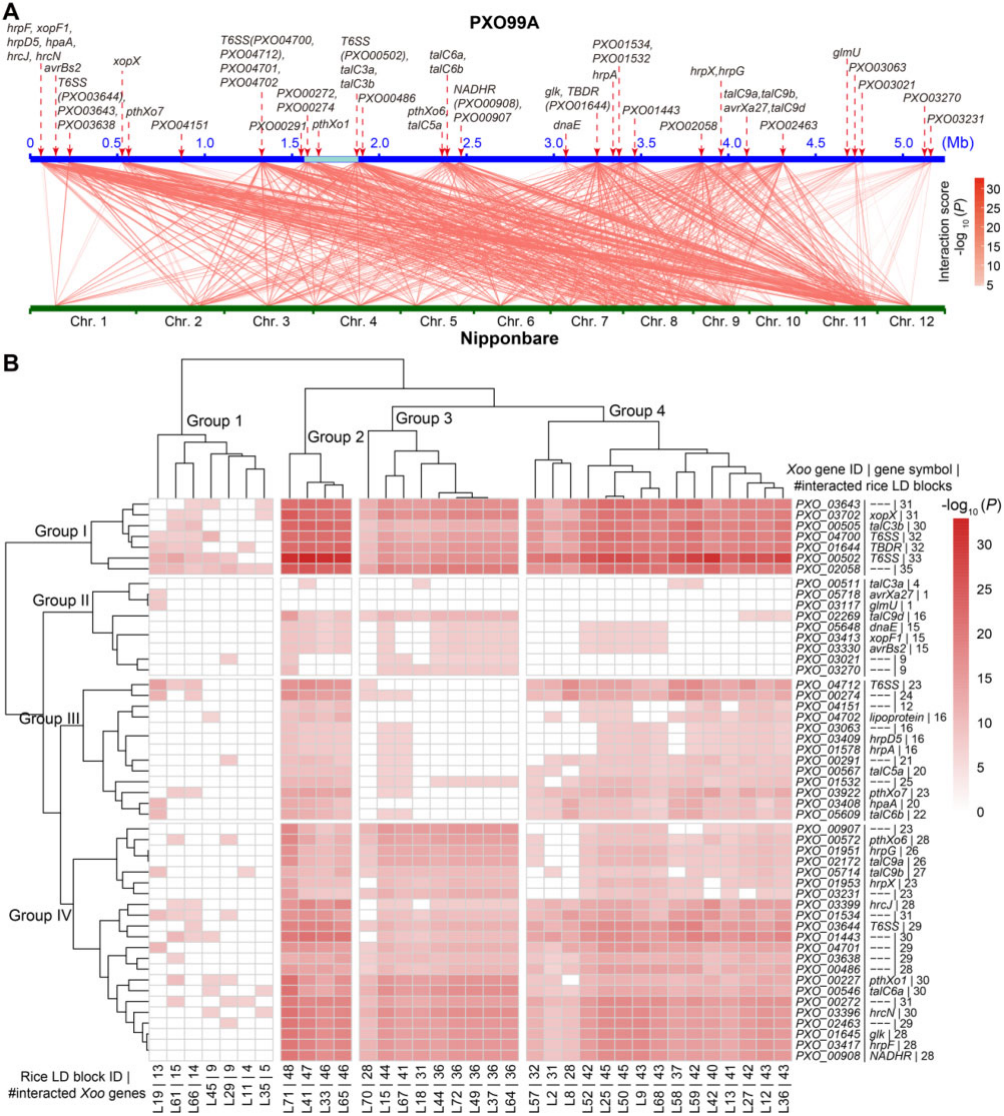
Genome-wide interactions between *Xoo* and rice detected by a cross-species 2D GWAS. A, 18,142 significant genome-wide SNP–SNP interactions between 625 SNPs within 51 *Xoo* genes and 59 SNPs within 35 rice LD blocks associated with resistance to *Xoo* detected by 2D GWAS. The *P*-value threshold for Bonferroni-corrected significance with the number of tests is set at 1.52 × 10^−7^ [0.05/(1,909 *Xoo* variants in virulence-related genes × 172 tag SNPs)]. *Xoo* genomic region highlighted with a cyan background is the recombination hotspot. Coordinates of the detected *Xoo* genes are displayed with red dotted arrows. B, A heatmap with two-way hierarchical clustering of genome-wide interactions between *Xoo* and rice shown in (A), in which the left side included all involved 51 *Xoo* virulence-related genes and the top side included all involved 35 rice LD blocks. The heatmap colors indicate the interaction intensity using the negative logarithmic values of the minimum *P*-values of the interaction signals if multiple significantly interacted SNPs were located in the same *Xoo* gene and the same rice LD block, respectively. The color scale for interaction intensity is shown on the right

We adopted a cross-species 2D GWAS strategy to identify the genetic interactions between *Xoo* and rice using the whole-genome sequencing data of 49 rice accessions and the 23 *Xoo* strains plus the lesion length data (*n *=* *49 × 23 = 1,127) of the 49 rice accessions inoculated by all 23 *Xoo* strains (see “Methods” for details). Practically, to limit the number of SNP pairs in the same LD blocks in the rice–*Xoo* interaction analysis, 75 rice LD blocks containing all significant SNPs detected in the rice GWAS panels were constructed and then their 172 tag SNPs were selected for rice–*Xoo* interaction analyses (Supplemental Data Set S6). Then, we examined interactions between 1,909 SNPs in *Xoo* virulence-related genes (known virulence genes and novel candidate genes detected from GWAS) and the 172 rice tag SNPs (Figure 7A; Supplemental Data Set S7). Based on a Bonferroni corrected threshold of *P *≤* *1.52 × 10^−7^ [0.05/(1,909 × 172)], we detected 18,142 significant SNP–SNP interactions between 625 SNPs within 51 *Xoo* genes and 59 SNPs within 35 rice LD blocks (Figure 7; Supplemental Data Set S8). The 51 virulence-related *Xoo* genes included 13 TAL effectors (*avrBs2*, *pthXo1*, *pthXo6*, *pthXo7*, *talC3a*, *talC3b*, *talC5a*, *talC6a*, *talC6b*, *talC9a*, *talC9b*, *talC9d*, and *avrXa27*), 2 non-TAL effectors (*xopF1* and *xopX*), 4 T6SS genes (*PXO*_*03644*, *PXO*_*04712*, *PXO_04700*, and *PXO*_*00502*), 7 T3SS genes (*hrpA*, *hrpD5*, *hrpF*, *hrpG*, *hrpX*, *hrcJ*, and *hrcN*), 1 *hrp*-associated gene *hpaA*, plus 24 other genes detected in the 1D GWAS. Most of these interactions showed a general “multiple-to-multiple” pattern, where a group of *Xoo* genes each interacted with a group of rice LD blocks distributed throughout the genome, and vice versa (Figure 7; Supplemental Data Set S8).

Based on the strengths (levels of statistical significance) and number of interactions, the 51 involved *Xoo* virulence genes and the 35 involved rice LD blocks could be roughly classified into four major interacting groups, i.e. *Xoo* gene groups I–IV versus rice QR-gene groups 1–4 (Figure 7B). *Xoo* gene group I was the most important group containing seven genes that interacted very strongly with rice QR-gene groups 2 and 4, strongly with rice QR-gene group 3 and weakly with rice QR-gene group 1. This group contains two T6SS genes (*PXO*_*00502* and *PXO*_*04700*), a non-TAL effector (*xopX*), a TAL effector (*talC3b*), a TBDR gene (*PXO*_*01644*), and two others (*PXO_02058* and *PXO_03643*). Also important was *Xoo* gene group IV containing 22 genes that interacted strongly with rice QR-gene groups 2–4. This group included five TAL effectors (*pthXo1*, *talC6a*, *pthXo6*, *talC9a*, and *talC9b*), five T3SS genes (*hrpF*, *hrpG*, *hrcJ*, *hrpX*, and *hrcN*), a T6SS gene (*PXO_03644*), a *NADHR* (*PXO_00908*), a *glk* (*PXO_01645*), and nine others (Figure 7B). *Xoo* gene group III has 13 genes, which interacted relatively weakly with rice QR-gene group 2 and most of the QR-gene group 4. This group includes a T6SS gene (*PXO_04712*), three TAL effectors (*talC5a*, *talC6b* and *pthXo7*), two T3SS genes (*hrpA* and *hrpD5*), an *hpaA* (*PXO_03408*), a *lipoprotein* (*PXO_04702*), plus five others. *Xoo* gene group II includes only nine genes, six of which (*talC9d*, *xopF1*, *avrBs2*, *dnaE*, *PXO_03021*, and *PXO_03270*) interacted weakly with most blocks in rice QR-gene groups 2 and 3. The remaining three genes (*talC3a*, *avrXa27*, and *glmU*) each interacted with 1–4 rice QR-gene blocks. One T6SS gene (*PXO*_*00502*) was the most important one that showed the strongest interactions with 33 rice LD blocks containing 174 rice genes. Other important *Xoo* genes included another T6SS gene (*PXO*_*04700*), a non-TAL effector (*xopX*), a TAL effector (*talC3b*), a TBDR gene (*PXO*_*01644*), and three other genes (*PXO_01443*, *PXO_02058*, and *PXO_03643*), which interacted more strongly and with more rice genes (Supplemental Data Set S8).

Similarly, rice QR-gene groups 2 and 4 were most important and interacted very strongly with *Xoo* gene group I and strongly with *Xoo* gene group IV (Figure 7B). The strongest interactions occurred between *Xoo* gene group I and QR-gene block L71 of 1.948 Mb on rice chromosome 11 containing seven NBS-LRR genes and 17 receptor-like protein genes. Moreover, most of the interacting rice genes within rice LD block L71 are enriched in pathways related to programmed cell death, protein amino acid phosphorylation, ATP binding, protein serine/threonine kinase activity, etc. (Supplemental Table S5). Furthermore, most rice genes that interacted with more *Xoo* genes are typical plant R-genes encoding signal-peptides, LRR, and tyrosine–protein kinase domains (Liu et al., 2014). Within rice LD block L71, *Xa3*/*Xa26* (*LOC_Os11g47210*) was the most important one that interacted strongly with four T6SS genes (*PXO_00502*, *PXO_04700*, *PXO_03644*, and *PXO_04712*), six TAL effectors (*talC3b*, *talC6a*, *pthXo1*, *pthXo6*, *talC9a*, and *talC9b*), five T3SS genes (*hrcN*, *hrpF*, *hrcJ*, *hrpG*, and *hrpX*), a non-TAL effector *xopX*, and 17 other *Xoo* genes. Rice QR-gene group 4 was also important and contains 15 LD blocks on nine rice chromosomes (1–4, 6–9, and 11) that interacted strongly with *Xoo* gene groups I and IV.

## Discussion

Most rice landraces in tropic Asia and China were known to lack resistance to *Xoo* (Ou and Jennings, 1969; Zhang, 2009) and no major epidemics of rice BB were documented until the early 1970s in Philippines and slightly later in China when the semi-dwarf varieties from the Green Revolution were spreading (Zhang, 1991; Mew et al., 1992). The *Xoo* strains used in this study represented 14 well-characterized races that emerged sequentially in the Philippines since late 1960s, while the Chinese strains represented nine major *Xoo* races collected from six provinces of China (Vera Cruz et al., 2000; Zhou et al., 2011; Quibod et al., 2016). Consistent with the previously reported Philippine race structure (Quibod et al., 2016), we found four Philippine races (P3c, P9a, P9b, and P9c) plus two Chinese races (GV and C5) belonged to the previous lineage PX-A and four Philippine races (P3b, P5, P7, and P8) constituted a second cluster as the previous lineage PX-B, while P6 and P6d formed the previous lineage PX-C (Figure 1C). However, the major difference was that six WV Chinese races (C1–C4, C6, and C7) plus two Philippine ones (P1 and P9d) formed a separate cluster plus three admixtures (P10, GIV, and P4). The close relationships of three MV and SV Chinese races (GIV, GV, and C5) from South China with those tropical *Xoo* races suggest they were more likely to have been introduced from the Philippines. The *Xoo* race structure depicted by the SNP data (Figure 1, B and C) was very similar to the grouping by the phenotypic data (Figure 1A), suggesting that the selection pressure from the host was the driving force shaping the *Xoo* race structure, which is in agreement with the previous study on Philippine races (Quibod et al., 2016).

Thus, we observed two consequences of the coadaptation and arms-race between rice and *Xoo* populations in tropic Asia and China. First, there is the strong differentiation among *Xoo* races corresponding to the well-known subspecific differentiation of rice. *Xoo* races from *Geng* accessions in the temperate areas of China all had weak virulence, while those on *Xian* accessions in the subtropic and tropic areas had moderate and strong virulence. Correspondingly, most QR-genes detected in *Xian* accessions are different from those in *Geng* accessions, suggesting a long history of reciprocal adaptation of rice and *Xoo* that may have started long before domestication. Second, the CHN-LAN population showed low levels of resistance to *Xoo* and had few detectable genes for BB resistance, which was accompanied by the weak virulence of the Chinese *Xoo* races. In contrast, modern *Xian* rice varieties had significantly improved resistance to *Xoo*. At the genomic level, we observed a high level of diversity at 3,735 (68.8%) of 5,432 significant SNPs across the rice genome at which significant frequency shifts for resistance alleles occurred during modern breeding, which was accompanied by a very high level of diversity at many virulence-loci (23 of 47 virulence-related genes with significantly higher SNP densities than other genome-wide background genes) in the *Xoo* populations. This correspondence in gene number and diversity between QR-loci in rice and virulence loci in *Xoo* has been the foundation of their reciprocal adaptation. Thus, our results revealed several important genomic features and genetic mechanisms of rice and *Xoo* that have been responsible for maintaining the observed genetic diversities at the resistance/virulence loci in the host plant and pathogen populations and for continuously generating “novel/new” resistance/virulence during the reciprocal adaptation between crops and their pathogens.

On the host side, as well demonstrated in lettuce (*Lactuca sativa*; Meyers et al., 1998) and in the case of *Xa21* of rice (Song et al., 1997), the most important mechanism for generating new and novel resistance genes/alleles is recombination in R-gene clusters present in plant genomes. For example, the *Xa21* locus is a receptor kinase gene cluster at ∼5 Mb upstream of regions R30–R37 on rice chromosome 11, in which recombination events in the highly conserved regions of the *Xa21* family members could result in precise swapping of promoter regions and generating new genes/alleles for novel resistance to *Xoo*. In this study, approximately half of the large-effect QR-genes were detected in the ∼3.3 Mb region of R30–R37 on rice chromosome 11 that contains many receptor-like kinases and NB-ARC gene clusters where most previously reported dominant *Xa*-genes (*Xa4, Xa22, Xa26, Xa32, Xa35, Xa36*, and *Xa40*) reside (Figure 3). We also observed a peak of large deletions in the R30–R37 region among the 701 rice genomes, implicating that recombination occurred frequently within this region (Figure 5C). Moreover, this region is characterized by high levels of SNPs, CNVs, and showed the most dramatic frequency shifts toward resistance alleles in modern varieties. Clearly, this region has been the primary targets of selection for high-level resistance to *Xoo* during modern breeding and recombination events in the R30–R37 region during breeding could easily generate “new” R-genes and/or R-gene combinations for novel resistance to new virulent races of *Xoo* (Zhang et al., 2015). One interesting observation was that most of the detected QR-genes against the SV race (C5) were of “small effect”, and the high-level resistance of 30 rice accessions could not be attributed to any single QR-genes but to associations of a set of “small-effect” genes (Supplemental Figure S10). This suggested novel high-level resistance could result from accumulation of many “small-effect” QR-genes from the CHN-LAN population. Experimental determination of the genetic relationships among these “small-effect” QR-genes leading jointly to high-level resistance to SV *Xoo* races are of great interest for future investigation.

On the pathogen side, mutations of large effect on traits associated with virulence and host adaptation are expected to evolve more rapidly and under strong positive selection in the modern crop–pathogen interaction systems. Also, rice responses to *Xoo* measured by lesion lengths using the leaf-clipping artificial inoculation method are known to be a highly heritable trait that, unlike most quantitative traits, shows minimum G × E (years and locations) interactions (Nayak et al., 1987; Li et al., 2001). Thus, these large-effect causal mutations in a pathogen population can be relatively easily detected even with small bacterial samples (Farhat et al., 2014). In this study, the problem of *Xoo* population stratification was controlled by using the first two principal components as covariates in GWAS using a linear mixed model (LMM). Thus, with the actual population size of 23 × 73 = 1,679 phenotypic observations for each of the 33,006 SNPs and the observed LD decay of 2.4 kb (∼3 genes) in the *Xoo* genome, we expected a reasonably high overall mapping resolution of the virulence-related loci identified in the *Xoo* population, similar to a previously reported case of phytopathogenic fungi (Gao et al., 2016). The false discovery rate of 0.17 of our GWAS estimated by permutation analysis was reasonable. Thus, most of the 47 detected virulence-related genes in *Xoo* should be real. We noted that 23 (∼49%) of these virulence loci showed significantly higher SNP diversity than the genome-wide background genes and more dramatic functional diversification. Of these, four TAL effectors, one T3SS gene (*hrpF*) and four T6SS genes showed extremely high levels of diversity and functional diversification among different *Xoo* races, indicating they had gone through a rapid evolution during their adaptation to rice host.

Most importantly, we discovered three groups of virulence-related genes. The first group included several T6SS genes involved in virulence of *Xoo*. T6SS effectors were reported to be important in fitness, virulence, and competence of pathogenic bacteria of several plant species (Ryu, 2015), including rice bacterial pathogen *Acidovorax avenae* subsp. *Avenae*, which causes rice bacterial brown stripe through the T6SS-mediated protein (toxic effectors) translocation (Basler and Mekalanos, 2012; Masum et al., 2017). The second group was the TBDRs. TBDRs in phytopathogenic bacteria play important roles in bacterial infection by obtaining iron through specifically targeting host iron-containing proteins (Parker Siburt et al., 2009; Grinter et al., 2016), by transporting specific carbohydrates across the host outer membrane (Blanvillain et al., 2007; Xu et al., 2013), or by mediating antibiotic resistance (Zhao et al., 1998). We found that many nonsynonymous SNPs in several TBDRs could clearly differentiate the 23 *Xoo* strains (Supplemental Figure S3, B and C). These results strongly suggested that TBDRs in *Xoo* had similar functions to acquire essential iron(s) and/or to transport carbohydrate(s) as required for *Xoo* infection and growth in rice (Supplemental Figure S3, D to G). The third group included three genes, NADH dehydrogenase (*PXO_00908*), ATP-dependent RNA helicase DbpA (*PXO_00394*), and ATP-dependent RNA helicase (*PXO_03270*), involved in energy metabolism. The SV *Xoo* races are differentiated strongly from the WV/MV races at the three loci, suggesting the adaptation of *Geng* and *Xian* types races to the rice host might have involved in energy use differences. Taking these findings together, recombination and mutation are expected to have played an important role in generating new and novel virulence to rice resistance and maintaining high levels of genetic diversity at many virulence-related genes in the tropical *Xoo* races even under the strong selection from the host populations during the coadaptation between rice and *Xoo*. This was because *Xoo* had been evolving at a much greater rate than rice, particularly in the tropic areas (Midha et al., 2017), and because various *Xoo* races of diverse virulence, including incompatible ones, are expected to be present in very different frequencies in the field populations of single modern rice varieties with high-level resistance.

Given the complex genetic systems underlying resistance/virulence in each case of plant–pathogen interactions regarding the numbers of loci involved, it has been a great challenge to characterize experimentally genome-wide interactions between defensive genes of a plant species and virulence genes of its pathogen because of the technical difficulty in phenotyping large numbers of plant accessions with many pathogen strains of diverse origins/virulence, particularly for crop plants. Here, we demonstrated that even with relatively small numbers of well-sampled rice accessions and *Xoo* races, it was possible to identify and shortlist causal rice QR-genes and *Xoo* virulence-genes involved in interactions underlying the reciprocal adaptation process between rice and *Xoo*. Statistically, the sample size of our data set (49 × 23 = 1,127) was expected to have sufficient power in detecting significant interactions between rice QR-gene blocks and *Xoo* virulence genes, though the rice sample size was too small to resolve causal QR-genes involved in specific interactions in most cases. While we adopted the tag SNPs of LD blocks each covering multiple significant SNPs in a single LD block as the input rice genotypic data to control the number of significant interactions detected in our 2D GWAS, the high mapping resolution of most QR-genes detected in our 1D GWAS allowed shortlisting of candidate QR-genes in many LD gene blocks involved in important interactions.

Based on current knowledge, the detected multiple-to-multiple interactions between the rice and *Xoo* genes were expected to be of two types in nature. The first type would result from direct interactions between rice R-genes and *Xoo* genes at the molecular level (protein–protein, protein–DNA, protein–RNA, DNA–DNA interactions, etc.) according to their functional correspondences, leading to high-level resistance/immunity or susceptibility (Niño-Liu et al., 2006; White and Yang, 2009) and the second type, described below, result from indirect genetic interactions. For the first type, previously reported cases of direct gene-for-gene interactions between *Xoo* TAL effectors and dominant rice *Xa*-genes are of this type, including *Xa10*/*avrXa10* (Tian et al., 2014), *Xa27*/*avrXa27* (Gu et al., 2005), *Xa23*/*avrXa23* (Wang et al., 2015a), and *Xa21/RaxX* (Pruitt et al., 2015). In this study, the strongest interactions between the seven genes of *Xoo* gene group I and genes of rice QR-gene groups 2 and 4 (particularly those in block L71) are most likely of this type, which may have played the most important role during the observed arms-race between rice and *Xoo* during modern breeding. These included the strongest interactions between *Xa26* (*LOC_Os11g47210*) and two T6SS genes (*PXO*_*00502* and *PXO*_*04700*), *hrpF* and seven *Xoo* genes (*xopX*, *talC3b*, *pthXo1*, *talC6a*, *pthXo6*, *talC9a*, and *talC9b*), and the interaction between *LOC_Os11g46890* (the *Xa40* candidate) and *hrpF*.

The direct interactions may also have occurred between some *Xoo* genes and their target rice susceptibility genes that function to acquire energy, essential nutrients, etc., for successful infection and growth of *Xoo*. Also, bacteria need resistance to plant antibiotics such as the phenazine-1-carboxylic acid in order to grow and reproduce normally (Wang et al., 2011). Thus, the (recessive) nonfunctional alleles at the involved rice susceptibility loci would act as recessive “r-genes”. Correspondingly, *Xoo* genes encoding enzymes involved in energy/nutrient/antibiotics metabolism and relevant transport systems may potentially be involved in this type of interaction, as demonstrated in the cases of *PthXo1*/*OsSWEET11* (Yuan et al., 2011), *PthXo2*/*OsSWEET13* (Zhou et al., 2015), and (*AvrXa7*, *PthXo3*, *TalC* and *Tal5*)/*OsSWEET14* (Blanvillain-Baufume et al., 2017) interactions. We believe that the strong interactions between *Xoo* TBDR (*PXO_01644*) and rice “r-genes” (iron-carrying proteins, such as a 2OG-Fe oxygenase family protein gene *LOC_Os11g43610*) may be of this type.

The finding that *Xoo* NADH may have played an important role in determining *Xoo* virulence was a big surprise because NADH dehydrogenase is well-known for its key role in energy metabolism for all bacteria (Heikal et al., 2014). In contrast to TBDRs, the *Xoo* NADH dehydrogenase gene, *PXO_00908*, is highly conserved harboring only two nonsynonymous SNPs (Supplemental Figure S12). Nevertheless, the two nonsynonymous SNPs distinguish seven SV *Xoo* races from the WV/MV races. Furthermore, the *Xoo* NADH interacts strongly with the same set of the rice genes as *hrpF* does (Figure 7B), suggesting that shifts between MV and SV of the closely related Philippine *Xoo* races caused by *hrpF* might be accompanied with a change or cost in their energy use associated with the corresponding functional changes in the NADH dehydrogenase protein.

Taking these results together, we have demonstrated an efficient strategy to identify and shortlist the numbers of important QR-genes/virulence-genes underlying the rice/*Xoo* coadaptation, which would greatly facilitate future efforts to elucidate the molecular mechanism(s) of the QR-gene–virulence-gene interactions underlying the coadaptation of rice and *Xoo*. Clearly, the power and mapping resolution of the 2D GWAS can be further improved by increasing the representativeness and sizes of sampled host genotypes such as well-designed mapping populations or near-isogenic line sets of QR-genes, and/or by increasing the pathogen population size. Nevertheless, our results support the concept that any specific plant–pathogen interaction involves complex immune receptor networks (Wu et al., 2018), within which the gene-for-gene theory would largely hold true in most cases of these direct interactions between plant R-genes and virulence genes in pathogens, though these direct interactions may not have to be one-for-one.

The second type of interactions between rice genes and *Xoo* genes are genetic and inferred to be indirect, resulting primarily from the functional complementarity between or among different types of *Xoo* virulence genes or rice defensive genes (genetic epistasis; Li et al., 2006), and most QR-gene blocks/virulence genes interactions detected in this study were expected to be of this type. Obviously, the observed multiple-to-multiple pattern of the interactions between rice genes and *Xoo* genes would arise in any of the following scenarios: (1) genetic epistasis arising from genes acting in the same defensive pathways of the host; (2) the co-existence of different allelic combinations of defensive genes in specific rice accessions and/or those of virulence genes in specific *Xoo* races (the small population size); and (3) the complex structure of the rice population used in the 2D GWAS. This is because a susceptible (compatible) phenotype would be expected only when none of the defensive genes in the host genome is effective or the pathogen genome contains the virulent alleles at all its virulence loci. Otherwise, a resistance phenotype would arise when a host plant carries one or more effective *Xa*/*xa* genes. Thus, it would be much more difficult genetically for a pathogen to adapt to its host genetic system containing large numbers of QR-genes. Fortunately, the short generation time (Grewal et al., 2012) and fast evolution rate of pathogens would allow them to generate sufficient new virulence genes/alleles by recombination and/or by mutation to overcome host resistance, and strong selection from host populations with new R-gene(s) would quickly to pick one or more virulent races from existing pathogen populations consisting of diverse races.

In summary, we demonstrated the power and efficiency to apply both 1D and 2D GWAS for genome-wide search for QR-genes/virulence-genes in rice and *Xoo* and their interactions underlying their coadaptation, which led to the identification of 47 *Xoo* virulence-related genes and 318 rice QR-genes located in 41 genomic regions, and genome-wide interactions between many of the detected virulence genes and QR-genes. The discovered QR-genes included previously reported *Xa26*, *xa25*, *Xa40* (experimentally validated) and *Xa22* plus many new ones. The new and novel virulence genes included several T6SS genes, TBDRs and many others. Recombination in the QR-gene clusters was suggested as the most important mechanism for generating novel resistance in rice to new virulent *Xoo* races, while recombination and mutation may have played an important role in generating new and novel virulence to rice resistance and maintaining high levels of genetic diversity at many virulence genes in *Xoo* populations. The relationship between rice and *Xoo* was characterized by strong differentiation among *Xoo* races corresponding to the subspecific differentiation of rice, by corresponding strong shifts toward increased resistance/virulence of rice/*Xoo* populations, by corresponding rich genetic diversity at the detected rice QR-genes and *Xoo* virulence genes, and by genome-wide interactions between many rice QR-genes and *Xoo* virulence genes in a multiple-to-multiple manner, presumably either from direct protein–protein interactions or from genetic epistasis. The observed complex genetic interaction system between rice and *Xoo* is expectedly to exist in other crop–pathogen systems, which would maintain high levels of diversity at their QR-loci/virulence-loci under strong directional selection from host populations, resulting in the dynamic coevolutionary consequences during their reciprocal adaptation. This relationship would last when a balance in evolutionary potential is reached, in which pathogens are depending primarily on their extraordinary abilities to evolve, while plants are dependent on their tremendous within-species genetic diversity and large complex genomes (more genes and more complex genetic systems). The arms-races between crop plants and their pathogens represent more extreme cases of the dynamic coevolutionary consequences of the complex genetic interaction system between pathogens and plants under strong artificial selection for high-level resistance/immunity that tends to break the balance between host plants and pathogens. In the end, the relationships between pathogens and their hosts would persist only if neither side wins.

## Materials and methods

### *Xoo* strains and artificial inoculation

Based on the previous determination of race grouping (Vera Cruz et al., 2000; Zhou et al., 2011; Quibod et al., 2016), the 23 *Xoo* strains representing 14 well-characterized *Xoo* races from tropical Asia (IRRI at the Philippines) and nine races from China collected during 1973–2012 (Supplemental Data Set S3) were used in this study. For the phenotyping experiments, seeds of individual rice (*Oryza sativa*) accessions were sown in the seedling nursery in the summers of 2015 (73 accessions for inoculation with all 23 strains) and 2016 (701 accessions for inoculation with four representative races C3 [JS158-2], C5 [GD1358], P1 [PXO61], and P9a [PXO339]), and 30-day-old seedlings of each accession were transplanted in paddy fields of experiment farms in the Institute of Crop Sciences, Chinese Academy of Agricultural Sciences, Beijing, China. Each of the rice accessions was planted into a 23-row plot for the first set of 73 rice accessions and a four-row plot for the second set of 701 rice accessions (the number of rows within a plot depended on the number of *Xoo* strains used for inoculation) with nine plants in each row at a spacing of 20 × 17 cm. The field planting followed a randomized complete block design with two experimental replications. All plants were managed under standard cultural practices. Each *Xoo* strain was incubated on peptone sucrose agar at 30°C for 2 days, and inoculum was prepared by suspending the bacterial mass in sterile water at a concentration of ∼10^8^ cells·mL^−1^.

At the tillering stage 30 days after rice seedlings were transplanted, plants were inoculated by the leaf-clipping method (Kauffman et al., 1973). For the accuracy of inoculation, only a single *Xoo* strain was used to inoculate rice plants in the same row. Briefly, four to five top fully expanded leaves from each of five and three central plants per row of each (accession) plot were clipped using scissors and then dipped into the *Xoo* inoculum for the first and second set of rice materials. The lesion lengths (cm) were measured 21 days after inoculation on two (three) leaves per plant of five (three) inoculated plants per experimental replication per *Xoo* strain for the first (second) set of rice materials. Thus, these combinations resulted in a total of 73 rice accessions × 23 *Xoo* strains × 5 plants × 2 replications for the first set of rice materials and 701 rice accessions × 4 *Xoo* strains × 3 plants × 2 replications for the second set of rice materials. Phenotypic data for each accession were defined as the average of the two experimental replications in each set of rice materials.

### Plant materials

Two nonoverlapping sets of plant materials were used in this study. The first set consisted of 73 rice accessions from 21 countries, including 49 accessions from the 3,000 Rice Genomes Project (3KRGP; 3K RGP, 2014) plus 24 additional accessions (Supplemental Data Set S2). The second set included 701 rice accessions from 40 countries (Supplemental Data Set S4), 515 of which were from 3KRGP plus 186 additional accessions composed mainly of modern Chinese varieties. According to the population structure, geographical origins, and variety types of accessions, we divided the 701 second-set accessions into different subpopulations, consisting of 419 *Xian* accessions, 219 *Geng* accessions, 451 Chinese accessions (135 landraces and 316 modern varieties), and 241 overseas accessions for specific comparisons of results.

### Heritability estimates

Broad-sense heritability was estimated using the conventional method of ANOVA with two experimental replications. The linear model for estimation of heritability is

$$y_{jk}=\mu +\alpha_{j}+\beta_{k}+\varepsilon_{jk}$$

where $y_{jk}$ is the lesion length of *j*th accession caused by one *Xoo* strain from *k*th replication, μ is the population mean, $\alpha_{j}$ is the genetic effect of the *j*th accession as a random effect following a $N(0,\sigma_{G}^{2})$distribution, $\beta_{k}$ is the effect of the *k*th replication, and $\varepsilon_{jk}$ is the residual error with an assumed$N(0,\sigma_{E}^{2})$ distribution. The broad-sense heritability is defined as

$$H^{2}=\frac{\sigma_{G}^{2}}{\sigma_{G}^{2}+\sigma_{E}^{2}}$$

The two variance components were estimated from the variance component analysis using the lme4 package (Bates et al., 2015) in R.

### Whole-genome sequencing and SNP calling of the 23 *Xoo* strains

Genomic DNA of each of the 23 *Xoo* strains was isolated using a modified cetyltrimethylammonium bromide (CTAB) protocol. Specifically, 2-mL cultures were grown in NYGB (nutrient yeast extract glycerol broth) for two days at 28°C under constant shaking. Cells were pelleted and re-suspended in Tris/ethylenediaminetraacetic acid. Cells were lysed in sodium dodecyl sulfate/CTAB extraction buffer before phenol: chloroform: isoamylalcohol extraction. DNA was precipitated in isopropanol and washed with 70% (vol/vol) ethanol. Quantity and quality of extracted DNA were assessed by a combination of nanodrop and gel electrophoresis. Next, the genomic DNA was fragmented by nebulization with compressed nitrogen gas. The overhangs of the fragments were converted to blunt ends using T4 DNA polymerase and Klenow polymerase. After adding an “A” base to the blunt ends of the double-stranded DNA fragments, adaptors with “T” base overhangs were ligated to the genomic DNA fragments. These fragments were separated on an agarose gel and excised from the gel at the DNA band around 200 bp. Finally, the DNA fragments were enriched by a ten-cycle polymerase chain reaction (PCR) process. The sequencing libraries with insert sizes of ∼500 bp and ∼6,000 bp were generated using a Covaris S-series instrument. We sequenced 100 bp at each end by Illumina Genome Analyzer IIx or Illumina HiSeq 2000. In total, each *Xoo* strain was sequenced with a high sequencing depth of ∼100×. After removal of sequencing adapters and cleaning of duplicated reads of PCR amplification, the clean reads were mapped to the PXO99^A^ genome (Salzberg et al., 2008) by BWA (V0.6.2; Li and Durbin, 2009) with default parameters. Reads realignment around indels were performed by Realigner Target Creator and Indel Realigner in GATK (V2.5-2; McKenna et al., 2010). SNPs were called by HaplotypeCaller in GATK (V2.5-2; McKenna et al., 2010) with default options, and SNPs were further filtered by SNP quality (QUAL > 30) and depth (DP ≥ 10).

### Whole-genome sequencing of 186 Chinese accessions and SNP calling of 701 rice accessions

Total genomic DNA was isolated from leaf tissues of each of the 186 Chinese accessions. A sequencing library with insert sizes of ∼500 bp was constructed for each accession according to Illumina’s standard instructions. Paired-end 90-bp reads were sequenced on Illumina HiSeq 2000 platform, and the raw sequences were further processed to remove adaptor sequences and low-quality reads, yielding approximately 5-Gb sequencing bases with an average sequencing depth of ∼15.2×, similar to sequencing data of the 3KRGP (3K RGP, 2014). All library construction, sequencing, and sequence cleaning were carried out by BGI-Shenzhen, China.

We further collected sequencing data of the 515 rice accessions from the 3KRGP (3K RGP, 2014). SNPs were called for all these 701 rice accessions. Specifically, high-quality reads were aligned to the Nipponbare reference genome (Kawahara et al., 2013) using the BWA software (V0.6.2; Li and Durbin, 2009) with the parameter “-m 10000 -o 1 -e 10 -t 4”. Aligned duplicated reads were then removed using Picard tools (V1.171; http://broadinstitute.github.io/picard/), reads around indels were realigned using GATK (V 2.0-35; McKenna et al., 2010) IndelRealigner, alignments were recalibrated by BaseRecalibrator in GATK. SNP calling was performed using UnifiedGenotyper in GATK with a minimum phred-scaled confidence threshold of 50, a minimum phred-scaled confidence threshold for emitting variants at 30, a minimum mapping quality at 20, and a minimum sequencing depth at 2. Finally, 8,661,876 high-quality SNPs were obtained for further analysis.

### Population analysis

We used the SNPhylo (V20160204; Lee et al., 2014) to construct the unrooted and unweighted neighbor-joining tree for the 701 rice accessions and the 23 *Xoo* strains by the Neighbor program. The genetic ancestry was inferred with STRUCTURE (V2.2.3; Pritchard et al., 2000). Principal component analysis was performed by EIGENSOFT(V6.0.1; Patterson et al., 2006) using pruned SNPs selected by PLINK (V1.90; Purcell et al., 2007) with the parameter “–indep-pairwise 50 10 0.2”. To estimate clonality of the *Xoo* population, MLGs were assigned by the poppr package (Kamvar et al., 2015) in R. By running the software HREfinder (Wang et al., 2013), with the information of SNP sites as input, we characterized the recombination events during the evolution and divergence of the 23 *Xoo* strains. The patterns of LD decay were calculated by PopLDdecay (V3.40; Zhang et al., 2019) in the whole rice population and PLINK in the whole *Xoo* population. Nucleotide diversity (*π*) for each 10-kb window across the genome with overlapping 5-kb step size was calculated using Variscan (V2.0.3; Vilella et al., 2005). Weir and Cockerham’s *F*_ST_ statistics at all significantly associated SNPs between rice subpopulations were calculated by VCFtools (V0.1.15; Danecek et al., 2011).

### Identification of virulence-related SNPs in *Xoo*

We integrated phenotypes of 73 rice accessions to detect virulence-related genes in *Xoo*. Existing multi-phenotype GWAS methods can only handle less than 10 phenotypes. Here, we used a customized strategy to integrate the GWAS results, which was demonstrated correct by revealing many known and highly possible virulence-related genes (see “Results” section for details). Specifically, we first carried out independent GWAS for *Xoo* SNPs with phenotyping data of lesion lengths from the first set of 73 rice accessions. Only 16,607 binary genic variants (SNPs within nonprotein-coding genes, transposases excluded) with MAF ≥0.05 were used for the association studies. We used both linear (LM) and LMMs, which took the population structure into consideration. Association tests based on LM were carried out using PLINK (V1.90; Purcell et al., 2007) with parameters “–linear”. Association tests based on LMM were carried out using EMMAX (Kang et al., 2010) with default parameters. An identical-by-state matrix based on all SNP data was used to create the kinship matrix that measures the genetic similarity between individual *Xoo* strains. The first two principal components were used as covariates to control for population structure. Finally, a CAS for each SNP (SNP *i*) defined as ${\mathrm{CAS}}_{i}={\mathrm{LM}}_{i}+{\mathrm{LMM}}_{i}$. LM and LMM are the integrated scores for LM and LMM and were calculated similarly as a weighted sum of 73 independent GWAS results: ${\mathrm{LM}}_{i}\mathrm{or}{\mathrm{LMM}}_{i}=\sum_{j=1}^{73}D_{j,i}$, where $\sum D_{i}$ is the sum of weighted scores of SNPs in the 73 independent LM/LMM GWAS

$$D_{j,i}=\{\begin{array}{c} 0,P_{j,i}>10^{-3}\\ \frac{1}{K_{j}},P_{j,i}\leq 10^{-3} \end{array}$$

in which, *K_j_* is the total number of significant SNPs (*P* <10^−3^) in the *j*th LM/LMM GWAS. Those SNPs with detected times less than 2 under both LM and LMM models, or with its minimum *P*-value among all tests higher than 10^−4^ were not considered as significantly associated. Finally, those SNPs with a CAS ≥0.3 were considered as significant associations. This threshold was determined by considering the ranks of the known and candidate virulence-related genes. Specifically, we first utilized three different *P*-value cutoffs (1e−3, 1e−4, and 1e−5) to calculate the CAS scores; next, by manually exploring literature in PubMed database (to see if a gene is reported as a virulence-related gene in other bacteria), we manually annotated 113 genes with CAS over 0.1 at each *P*-value cutoff; we then assessed the performance of combinations of the *P*-value and CAS score cutoffs by assuming that all the candidate pathogen genes were true positives, the combination of the *P*-value cut-off at 0.001 and CAS cutoff at 0.3 gave the highest overall accuracy (the average value of sensitivity and specificity at the genic level). We carried out genotype permutation analysis to estimate the false discovery rate. Specifically, we shuffled the genotypes (exchange “whole” genotypes among the 23 *Xoo* strains) and performed the CAS-based GWAS. We did 10,000 permutations and for each permutation, 15.0 ± 8.0 (mean ± sd of the mean) SNPs could be flagged as significant [as described previously in the method section: CAS ≥ 0.3 (calculated at 0.001), detected times ≥2 and minimum *P* <0.0001], which suggested a low SNP-level false discovery rate of 15/86 (the significant SNP number detected in this article) = 0.17.

### Phylogeny and positive selection analysis of *Xoo* virulence genes

In order to obtain evidence for positive selection of two virulence-related genes (*PXO_03417* and *PXO_01644*) during the evolution and divergence of *Xoo*, the CDSs of all genes from the 23 *Xoo* strains were built artificially by replacing SNPs to their corresponding CDS in the PXO99^A^ reference genome and aligned by using PHYML (V3.0; Guindon et al., 2010), and then phylogenetic trees were inferred using the maximum likelihood method accordingly. The software was set to use a BIONJ start tree, the JTT substitution model, the default NNI tree searching method, gamma-distributed rates of evolution with four discrete categories and empirical nucleotide frequencies. Nonparametric bootstrap repetitions were used to evaluate statistical support to branches. Rooting was performed using the midpoint method. The resulting phylogenetic trees were drawn with iTOL (V4; Letunic and Bork, 2019). Sequence alignments and phylogenetic tree files can be found in Supplemental File S1. By comparing a free parameter model (dN/dS is free) and a restricted parameter model (dN/dS is restricted to be 1 on individual tree branches) to detect positive selection genes in the *Xoo* genome, and positively selected amino acid residues in proteins (NSsites model) were inferred using the codeml program from the PAML software package (Yang, 2007). The dN and dS values were estimated using the nonsynonymous and synonymous nucleotide substitutions along lineages on the phylogenetic trees.

### Identification of rice resistance genes by GWAS

A total of 5,379,674, 5,359,202, 2,932,149, 5,003,609, 5,828,337, 5,168,652, and 4,784,025 SNPs with minor allele frequencies >5% and the number of accessions with minor alleles ≥6 were filtered for the association analyses of the whole population, *Xian*, *Geng*, Chinese, overseas, Chinese landrace, and Chinese modern variety panel populations, respectively. All GWAS were completed using a LMM in EMMAX (Kang et al., 2010) to determine the associations between each SNP and phenotyping data of lesion lengths from the second set of 701 rice accessions for resistance to four representative *Xoo* races (C3, C5, P1, and P9a). We used the Balding–Nichols matrix based on LD pruned SNPs across the whole rice genome (with parameter “indep-pairwise 50 10 0.2” in PLINK) to develop the kinship matrix, which measured the genetic similarity between individuals in each GWAS panel. The first three principal components were used as covariates to control for population structure. The effective number of independent markers (N) was calculated using the GEC software (Li et al., 2012b), and significant thresholds (0.05/N) were calculated in each GWAS panel. To account for the effects of replication, the best linear unbiased predictions (BLUPs) for the lesion lengths of the two replications were calculated with the lmer function of the R package lme4 (Bates et al., 2015), using rice accession as random effect and replication as fix effect (Supplemental Data Set S4). Mean lesion lengths and BLUPs of the two replications were both used as the phenotypic input for the association analysis. Because both approaches produced very consistent results, only the results based on the phenotypic means across two replications are presented. Adjacent lead SNPs separated by <300 kb were defined as a single association region based on the LD decay in the whole population and the method described by Chen et al. (2014) and Wang et al. (2015b). Gene symbols of functionally characterized rice genes were obtained from funRiceGenes database (Yao et al., 2018). The Manhattan and quantile–quantile plots for the GWAS results were created using R. Enriched GO terms (FDR < 0.05) were identified using the agriGO v2.0 with the Singular Enrichment Analysis method (Tian et al., 2017).

### Vector construction and rice transformation

To analyze the functions of the predicted candidate genes, the CRISPR/Cas9 binary vector of *LOC_Os11g46890* in a resistant haplotype variety, Wuyugeng20, was constructed according to the multiplex editing method of Ma et al. (2015). Specifically, two guide RNAs were designed to target exon of *LOC_Os11g46890* using the web-based software CRISPR-GE (http://skl.scau.edu.cn/;Xie et al., 2017). To construct the guide RNA constructs for target site 1, the first PCR was carried out in two separate reactions with U-F/U6a-LOC_Os11g46890 and gRT1-LOC_Os11g46890/gR-R using pYLsgRNA-OsU6a as a template. Then the second PCR was performed to generate a guide RNA construct containing target site 1 by overlapping PCR with the first PCR products. Likewise, to construct the guide RNA constructs for target site 2, the first PCR was carried out in two separate reactions with U-F/U6b-LOC_Os11g46890 and gRT2-LOC_Os11g46890/gR-R using pYLsgRNA-OsU6b as a template. Then the second PCR was performed to generate a guide RNA construct containing target site 2 by overlapping PCR with the first PCR products. Finally, these two guide RNA constructs were inserted into the pYLCRISPR/Cas9Pubi-H vector through restriction–ligation reaction. The transgenic plants were created by the *Agrobacterium*-mediated transformation method (Hiei et al., 1994). To confirm the mutations, we designed primer *LOC_Os11g46890*-TF and *LOC_Os11g46890*-TR to amplify the fragment spanning the two target sites in the T_0_ transgenic lines. Through Sanger sequencing with gene-specific sequencing primer *LOC_Os11g46890*-SP, the homozygous mutants in target sites were identified. See Supplemental Table S6 for the primer sequences used for vector construction and mutation detection. The resistance levels of the mutants and Wuyugeng20 were determined by inoculation with *Xoo* race C5 at the tillering stage 30 days after rice seedling transplanting using the leaf-clipping method (Kauffman et al., 1973). Briefly, five top fully expanded leaves from each of three plants of each T_1_ mutant and Wuyugeng20 were clipped using scissors dipped with the *Xoo* inoculum. The water-soaked lesion lengths (cm) of nine leaves (three leaves per plant) of three inoculated plants from each mutant and Wuyugeng20 were measured 21 days after inoculation.

### Cross-species genome–genome interaction analysis

Of the 73 rice accessions of the first set, 49 are sequenced in 3KRGP. Using the SNP genotypes at the detected virulence loci of *Xoo*, resistance loci of rice and the phenotypes (mean lesion lengths of two replications) of the 49 rice accessions inoculated by the 23 *Xoo* strains, we were able to analyze all possible pairwise interactions between the detected virulence loci of *Xoo* and resistance loci of rice statistically using a linear model via a custom-made script in R, which is available on Github (https://github.com/doodlehzq/TwoWayGWAS). Specifically, to test the interaction between the *p*th rice SNP and the *q*th *Xoo* SNP, we fit the below linear model:

$$L∼\beta_{0}+\beta_{1}M_{p}+\beta_{2}N_{q}+\beta_{3}M_{p}N_{q}+\left\{\beta_{4}R_{1}+\cdots +\beta_{51}R_{48}\right\}+\left\{\beta_{52}X_{1}+\cdots +\beta_{73}X_{22}\right\}.$$

*L* {*L*_1,1_, *L*_1,2_,…, *L*_1,23_, *L*_2,1_,…, *L_i,j_*,…, *L*_49,23_} is a phenotype vector (with size of 49 × 23) between 49 rice accessions and 23 *Xoo* strains. *L_i,j_* denotes the lesion length of *j*th *Xoo* strain on *i*th rice accession. *M_p_* {*M*_1,__*p*_,*M*_1,__*p*_,…,*M*_1,_*_p_* (i.e. *M*_1,__*p*_ was repeated 23 times), …, *M_i,p_*,…,*M_i,p_*, …, *M*_49,__*p*_,…,*M*_49,__*p*_} is the *p*th genotypes of the corresponding rice accession, and *N_q_* {*N*_1,__*q*_,*N*_2,__*q*_,…,*N*_23,_*_q_* (i.e. a genotype set of the 23 *Xoo* strains), *N*_1,__*q*_,…,*N_j,p_*,…,*N*_23,__*q*_, …(the genotype set was repeated a total of 49 times)…} is the *q*th genotypes of the corresponding *Xoo* strain. *M_p_N_q_* denotes the interaction variable, which was our interest. *R*_1_, …, *R*_48_ are 48 covariates displaying if a phenotype involved a specific rice accession. For example, *R*_1_ {49 times of 1 followed by 49 × 22 times of 0} represents if the phenotypes were obtained from the first rice accession. Similarly, *X*_1_, …, *X*_22_ were 22 covariates displaying if a phenotype involved a specific *Xoo* strain. Here, to test if there was a significant interaction between *M_p_* and *N_q_*, the null hypothesis is *β*_3_ = 0.

Practically, to limit the number of SNP pairs in the same LD blocks in rice–*Xoo* interaction analysis, we firstly constructed local LD structures around 5,432 significant SNPs detected by the 1D GWAS in the second set of rice materials and selected independent representative SNPs (for the four *Xoo* races) with the most significant hit and/or with the largest contribution to the phenotypic variance for each *Xoo* race within an LD block. Specifically, rice LD blocks were identified to select independent significant association peaks in rice GWAS panels using PLINK command line: “plink –clump –clump-p1 6.81e-8 –clump-p2 1e-5 –clump-r2 0.5 –clump-kb 300”. A total of 75 LD blocks were identified for the four *Xoo* races, including 28, 23, 46, and 20 LD blocks associated with C3, C5, P1, and P9a, respectively. Then, we identified two representative SNPs for each *Xoo* race, one with the lowest *P*-value in 1D GWAS (i.e. the SNP identified by the clump function in PLINK) and one with the largest contribution to the phenotypic variance estimated with the statistical model described by Zhao et al. (2011) within each LD block. Both SNPs were considered as tag SNPs for this LD block to conduct further cross-species interaction analysis. Consequently, a total of 172 tag SNPs within the 75 LD blocks in the rice genome (Supplemental Data Set S6) were selected for determining significant rice–*Xoo* interactions. Then, we tested interactions (328,348 possible pairs) between 1,909 SNPs in two types of *Xoo* genes ((1) known virulence-related genes in Supplemental Table S1 and (2) genes associated with virulence detected by *Xoo* GWAS) and the 172 rice tag SNPs. Using the Bonferroni correction for multiple testing with the number of tests we conducted here, the *P*-value threshold for significant interaction was 1.52 × 10^−7^ [0.05/(1,909 *Xoo* variants in virulence-related genes × 172 rice tag SNPs)]. Finally, significant interactions between all pairs of SNPs were binned into gene-to-block interactions between *Xoo* and rice. For example, when multiple significantly interacted SNPs were located in the same *Xoo* gene and the same rice LD block, respectively, only the strongest SNP–SNP interaction (with the minimum *P*-value) was retained as the gene-to-block interaction.

### The density of large-sized deletions

Based on structural variations detected in the 3KRGP (Wang et al., 2018b), in which deletions with size >100 bp were detected, we calculated the genome-wide deletion densities with the sliding window method. Briefly, the average numbers of deletions were calculated in 500-kb windows that slid forward by 100 kb each time.

### Statistical analysis

The whole GWAS panel SNPs within 1 kb of the upstream promoter region, 3′-UTR, 5′-UTR, and nonsynonymous SNPs in the coding region of a rice candidate gene or the GWAS panel SNPs within the genomic region of an *Xoo* candidate gene were concatenated as the haplotype of resistance or virulence loci identified by GWAS, respectively. SNP annotation information was obtained from Rice SNP-Seek Database (Mansueto et al., 2017). For multiple group comparison of mean lesion lengths of the major haplotypes of resistance loci (shared by at least 10 accessions), Tukey’s HSD post hoc tests followed by one-way ANOVA were carried out with the agricolae package in R. Pearson’s Chi-squared tests using the “chisq.test” function in R were performed to determine significant differences in frequencies of different reactions in rice subpopulations to each *Xoo* race and significant differences in frequencies of QR-gene haplotypes among rice subpopulations. A collection of the data for statistical analyses can be found in Supplemental Data Set S9. Enrichment significance of SNP density was detected with the “phyper” function in R for tests of the hypergeometric distribution. Fisher’s exact tests by the “fisher.test” function in R were used to compare differences in frequencies of detected virulence-related genes or SNPs within and outside the recombination hotspot in *Xoo* genome. ANOVA was performed to partition the variance components for lesion lengths due to the effects of the replications, rice accessions, *Xoo* strains, and the interactions between rice accessions and *Xoo* strains using the “aov” function in R.

### Accession numbers

The datasets supporting the conclusions of this article are included in the article. Raw sequence data of 23 *Xoo* strains are available in the Genome Sequence Archive in National Genomics Data Center (https://bigd.big.ac.cn/gsa) under accession number CRA004049. Raw sequence data and the SNP dataset in PLINK format of 186 Chinese accessions are available at Rice Functional Genomics and Breeding Database (http://www.rmbreeding.cn/tool/dl_rice701).

## Supplemental data

The following materials are available in the online version of this article.

**Supplemental Figure S1**. Genomic recombination, LD decay and nucleotide diversity of 23 *Xoo* strains.

**Supplemental Figure S2**. Positive selection and haplotypes of the *hrpF* gene (*PXO_03417*).

**Supplemental Figure S3**. SNP distribution and positive selection of the TonB-dependent receptor gene (*PXO_01644*) across the 23 *Xoo* strains and the effect of the nonsynonymous mutation (SNP position 3248766) on 3D structure.

**Supplemental Figure S4**. Population structure and LD decay of the 701 rice accessions.

**Supplemental Figure S5**. Quantile–quantile plots of genome-wide association studies for P1, P9a, C5, and C3 in different panel populations of rice.

**Supplemental Figure S6**. Candidate QR-genes to bacterial blight identified in the genome-wide association analyses.

**Supplemental Figure S7**. Haplotype analysis of *xa25* (*LOC_Os12g29220*) and *Xa26* homolog (*LOC_Os11g47240*).

**Supplemental Figure S8**. Comparison of the predicted proteins in wild type Wuyugeng20 (WYG20) and three knockout mutants of *LOC_Os11g46890*.

**Supplemental Figure S9**. Haplotype analysis of *LOC_Os11g46250*.

**Supplemental Figure S10**. The graphical genotype showing coexistence of the resistance alleles at eight QR-loci jointly determining high-level resistance to SV *Xoo* race C5.

**Supplemental Figure S11**. Extended and detailed differences in frequencies of resistance alleles, nucleotide diversity, and copy number of the rice QR-genes.

**Supplemental Figure S12**. The SNP distribution in NADH dehydrogenase gene (*PXO_00908*) across the 23 *Xoo* strains.

**Supplemental Table S1**. Summary of SNPs in the 48 known virulence-related genes previously reported in *Xoo*.

**Supplemental Table S2**. Candidate SNPs associated with *Xoo* virulence (lesion length) detected by GWAS.

**Supplemental Table S3**. Twenty-three genes highly associated with *Xoo* virulence (lesion length) detected by GWAS.

**Supplemental Table S4**. Forty-one genomic regions <300 kb each containing >5 significant SNPs for resistance to 1–4 *Xoo* races detected in the second set of rice materials.

**Supplemental Table S5**. Results of a GO enrichment analysis of the detected rice genes that interacted with *Xoo* virulence-related genes.

**Supplemental Table S6**. Information for vector constructions and primers.

**Supplemental Data Set S1**. The reactions of 73 rice accessions in the first set of rice materials with 23 *Xoo* strains.

**Supplemental Data Set S2**. List of the 73 accessions of the first set of rice materials.

**Supplemental Data Set S3**. Summary of whole-genome sequencing information of 23 *Xoo* strains.

**Supplemental Data Set S4**. The reactions of 701 accessions in the second set of rice materials with four representative *Xoo* races.

**Supplemental Data Set S5**. The list of total 5,432 significant SNPs associated with resistance to 1–4 *Xoo* races detected in the second set of rice materials.

**Supplemental Data Set S6**. Summary of the 75 LD blocks around significant SNPs identified in rice GWAS.

**Supplemental Data Set S7**. Complete list of genome-wide pairwise SNP-SNP interactions of rice genes associated with bacterial blight resistance and virulence-related genes of *Xoo* based on lesion lengths.

**Supplemental Data Set S8**. Genome-wide interactions of rice LD blocks associated with bacterial blight resistance and *Xoo* virulence-related genes identified in Supplemental Data Set S7.

**Supplemental Data Set S9**. Summary of statistical tests.

**Supplemental File S1**. Sequence alignments and phylogenetic tree files of two *Xoo* genes (*PXO_03417* and *PXO_01644*).

## Supplementary Material

koab146_Supplementary_Data

## Notes

Fan Zhang, Zhiqiang Hu and Zhichao Wu contributed equally to this work (F.Z., Z.H., Z.W.).

Z.L., Y.Z., and F.Z. conceived and designed the experiments; F.Z., J.L., Y.S., F.Z., C.V.C., M.W., X.S., D.Z., D.H., W.W., X.Z., T.Z., J.X., B.F., and J.A. performed the experiments; Z.H., F.Z., Z.W., X.W., J.W., Y.C., and M.L. analyzed the data; Z.L., F.Z., Z.H., and Y.Z. wrote the manuscript; and all authors read and approved the final manuscript.

The authors responsible for distribution of materials integral to the findings presented in this article in accordance with the policy described in the Instructions for Authors (https://academic.oup.com/plcell) are: Zhikang Li (lizhikang@caas.cn) and Yongli Zhou (zhouyongli@caas.cn).
