# Supplementary material for: Reciprocal adaptation of rice and Xanthomonas oryzae pv. oryzae: cross-species 2D GWAS reveals the underlying genetics
Source: Plant Cell. 2021 Jun 2;33(8):2538–61. doi: 10.1093/plcell/koab146 (PMC8408478; doi:10.1093/plcell/koab146)
Supplement: koab146_Supplementary_Data [file koab146_supplementary_data.zip › tpc.01058.2020-s01.pdf]

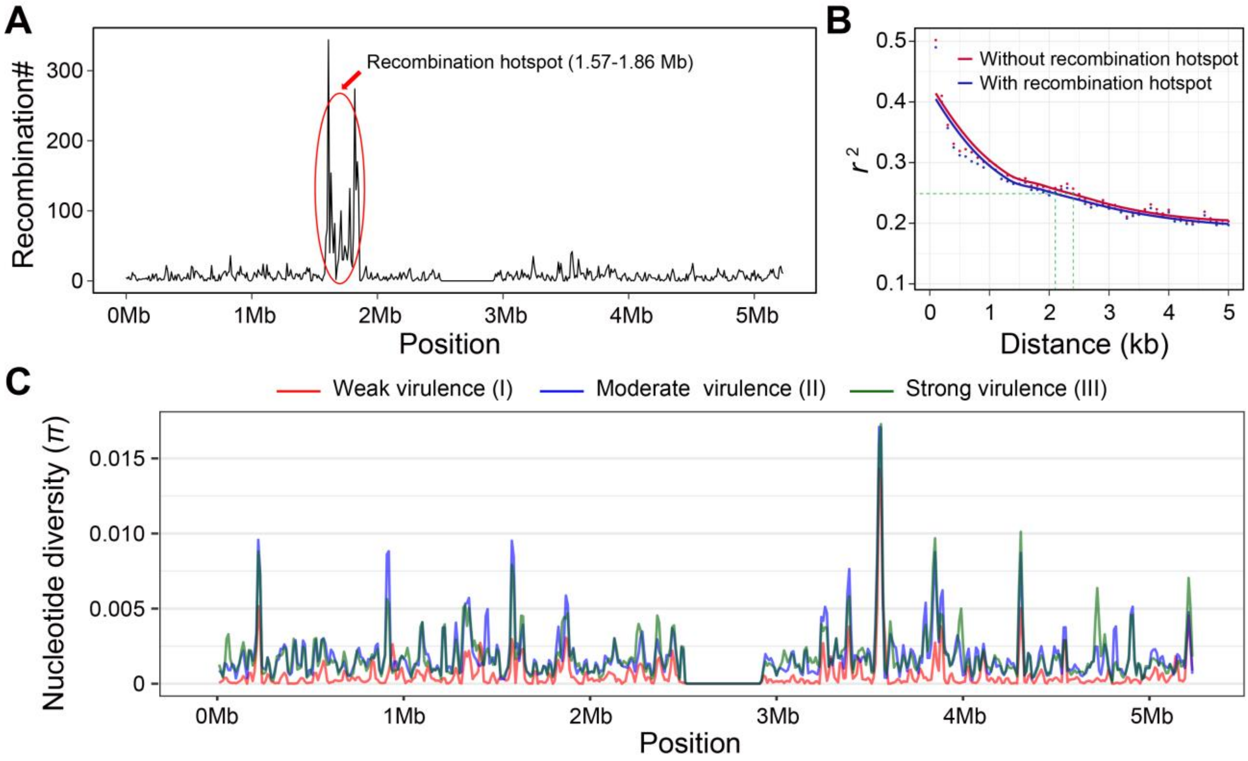

Supplemental Figure S1. Genomic recombination, linkage disequilibrium (LD) decay and nucleotide diversity of 23 *Xoo* strains. (Supports Figure 1)

(**A**) Distribution of recombination events during the evolution of 23 *Xoo* strains. The red arrow points to the recombination hotspot. (**B**) LD decay curves across the *Xoo* genomes with and without the recombination hotspot. The decay of LD was measured by r^2. (**C**) Nucleotide diversity across the *Xoo* genomes. Red, blue, and green lines represent weak virulence, moderate virulence, and strong virulence *Xoo* races.

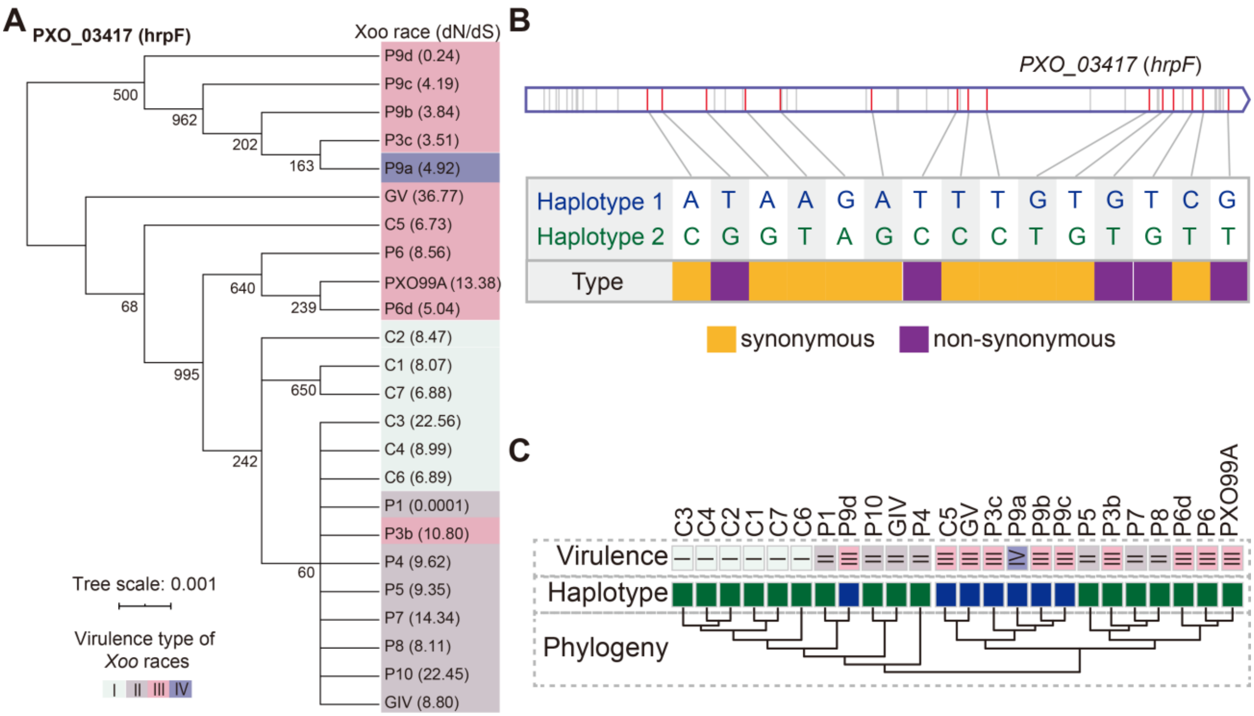

Supplemental Figure S2. Positive selection and haplotypes of the *hrpF* gene (*PXO_03417*). (Supports Supplemental Tables S2 and S3)

(**A**) Phylogenetic and positive selection analysis of *hrpF* across the 23 *Xoo* strains. The CDSs of all genes from the 23 *Xoo* strains were built artificially by replacing SNPs to their corresponding CDS in the PXO99^{A} reference genome and were aligned by using PHYML and then the phylogenetic tree was inferred accordingly. Branch support is obtained from 1000 non-parametric bootstrap repetitions. Branches with a bootstrap value less than 50 are deleted, and the bootstrap values are indicated on tree. The bar of tree scale is 0.001 amino acid substitutions per site. Gene positive selection was detected by comparing a free parameter model (dN/dS is free) and a restricted parameter model (dN/dS is restricted to be 1 on certain tree branches), and to infer positively selected amino acid residues in proteins (NSsites model) using the software PAML:codeml, in which dN and dS were estimated using the nonsynonymous and synonymous nucleotide substitutions along lineages on the phylogenetic trees. (**B**) SNPs and haplotypes of *hrpF*. (**C**) The distribution of the two *hrpF* haplotypes in different *Xoo* strains.

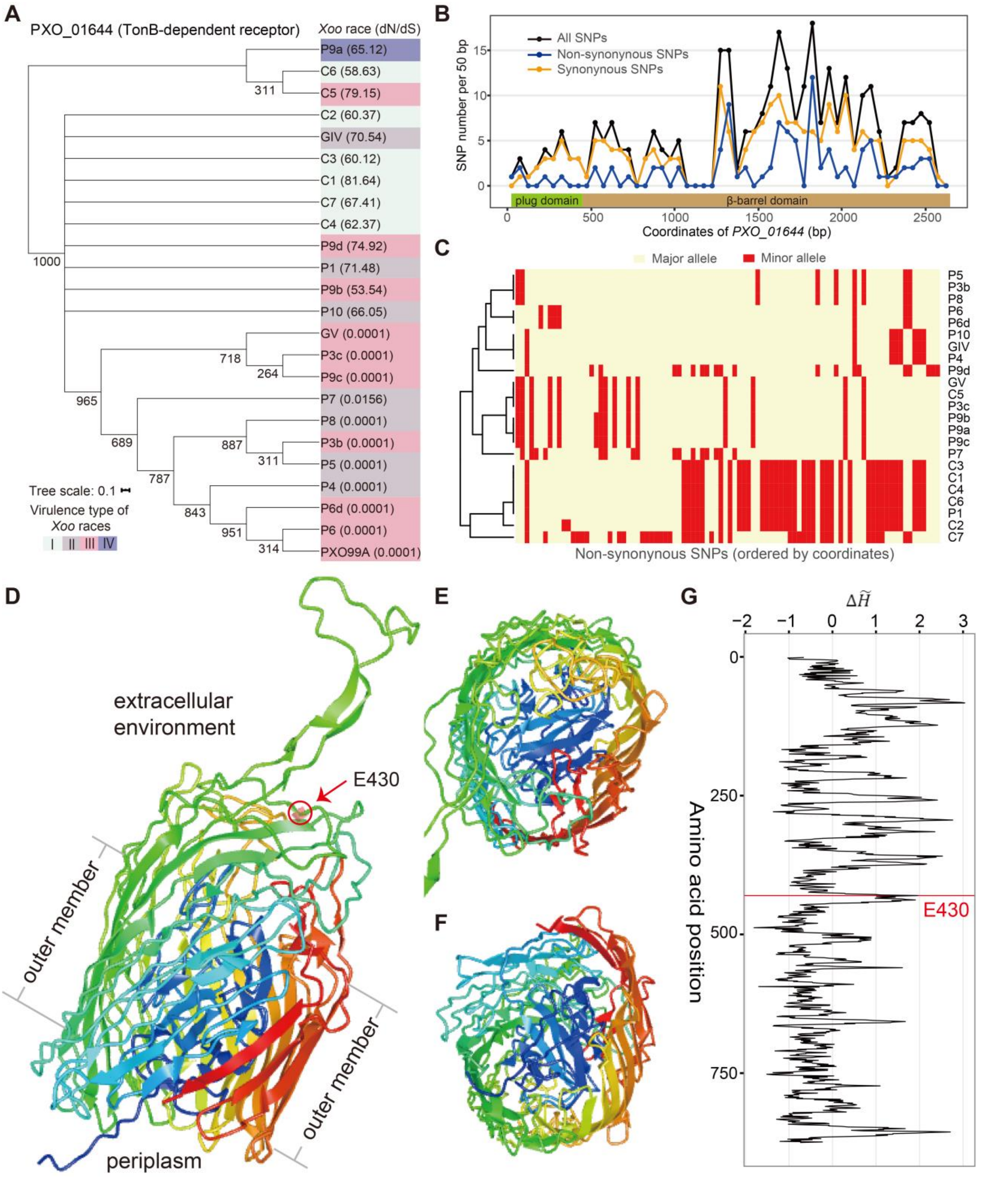

Supplemental Figure S3. SNP distribution and positive selection of the TonB-dependent receptor gene (*PXO_01644*) across the 23 *Xoo* strains and the effect of the nonsynonymous mutation (SNP position 3248766) on 3D structure. (Supports Supplemental Tables S2 and S3)

(**A**) Phylogenetic and positive selection analysis of *PXO_01644* across the 23 *Xoo* strains. The CDSs of all genes from the 23 *Xoo* strains were built artificially by replacing SNPs to their corresponding CDS in the PXO99^{A} reference genome and were aligned by using PHYML and then the phylogenetic tree was inferred accordingly. Branch support is obtained from 1000 non-parametric bootstrap repetitions.

Branches with a bootstrap value less than 50 are deleted, and the bootstrap values are indicated on tree. The bar of tree scale is 0.1 amino acid substitutions per site. Gene positive selection was detected by comparing a free parameter model (dN/dS is free) and a restricted parameter model (dN/dS is restricted to be 1 on certain tree branches), and to infer positively selected amino acid residues in proteins (NSsites model) using the software PAML:codeml, in which dN and dS were estimated using the nonsynonymous and synonymous nucleotide substitutions along lineages on the phylogenetic trees. (**B**) Distribution of SNPs displayed by a non-overlapping 50-bp sliding window. This gene showed extremely high SNP densities (11.6%), with 305 SNPs including 209 synonymous and 96 nonsynonymous SNPs located in 2,628 bps. Synonymous and nonsynonymous SNPs are labeled with orange and blue respectively. (**C**) The heatmap of 96 nonsynonymous SNPs ordered by their coordinates. *Xoo* strains are grouped with hierarchical clustering. (**D**) A global view of the 3D structure of the protein predicted with the intensive mode of Phyre2 website; the amino acid substitution at position 430 is labeled. (**E**) View from the extracellular environment. (**F**) View from the periplasm. (**G**) Estimation of the active sites with a fuzzy-oil-drop model. Amino acids with a higher delta *H* are predicted to be active sites of the protein.

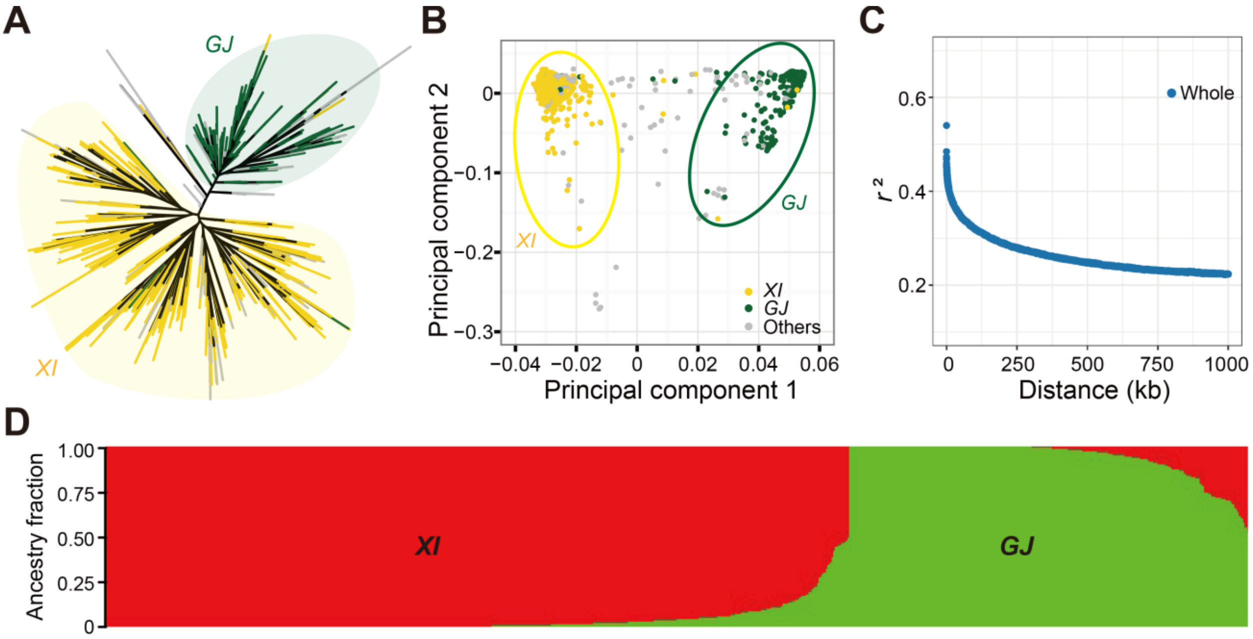

Supplemental Figure S4. Population structure and linkage disequilibrium (LD) decay of the 701 rice accessions. (Supports Figures 2 and 3)

(**A**) The neighbor-joining tree constructed from LD pruned SNPs. (**B**) Principal component analysis plots for the first two components of the 701 accessions. (**C**) LD decay in the whole population. The decay of LD was measured by r^2. (**D**) The distribution of the estimated subpopulation components (ancestry fraction) of each accession analyzed by STRUCTURE. *XI*, *Xian*/*indica*; *GJ*, *Geng*/*japonica*.

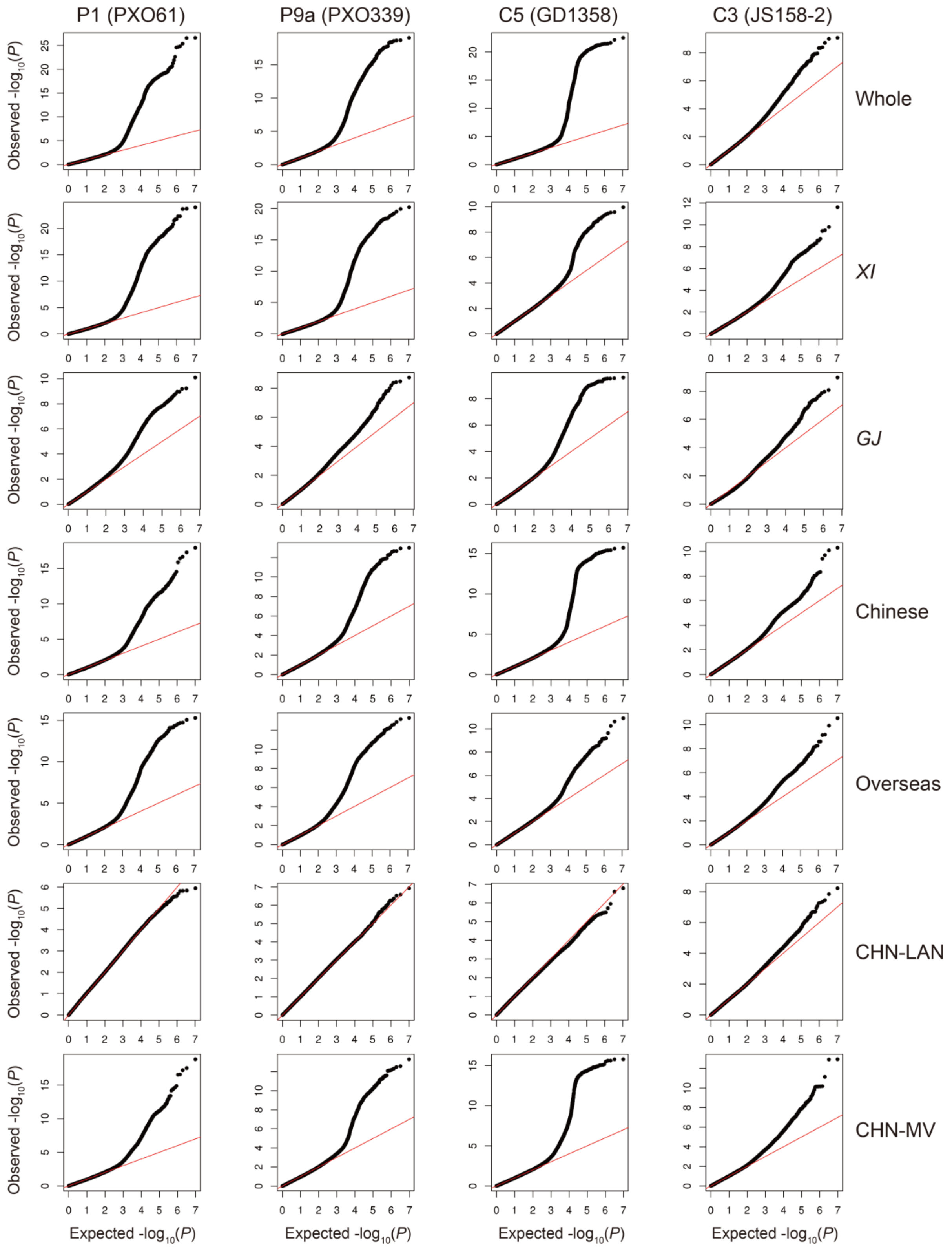

Supplemental Figure S5. Quantile-quantile plots of genome-wide association studies for P1, P9a, C5 and C3 in different panel populations of rice. (Supports Figure 3)

XI, *Xian*/*indica*; *GJ*, *Geng*/*japonica*; CHN-LAN, Chinese landraces; CHN-MV, Chinese modern varieties.

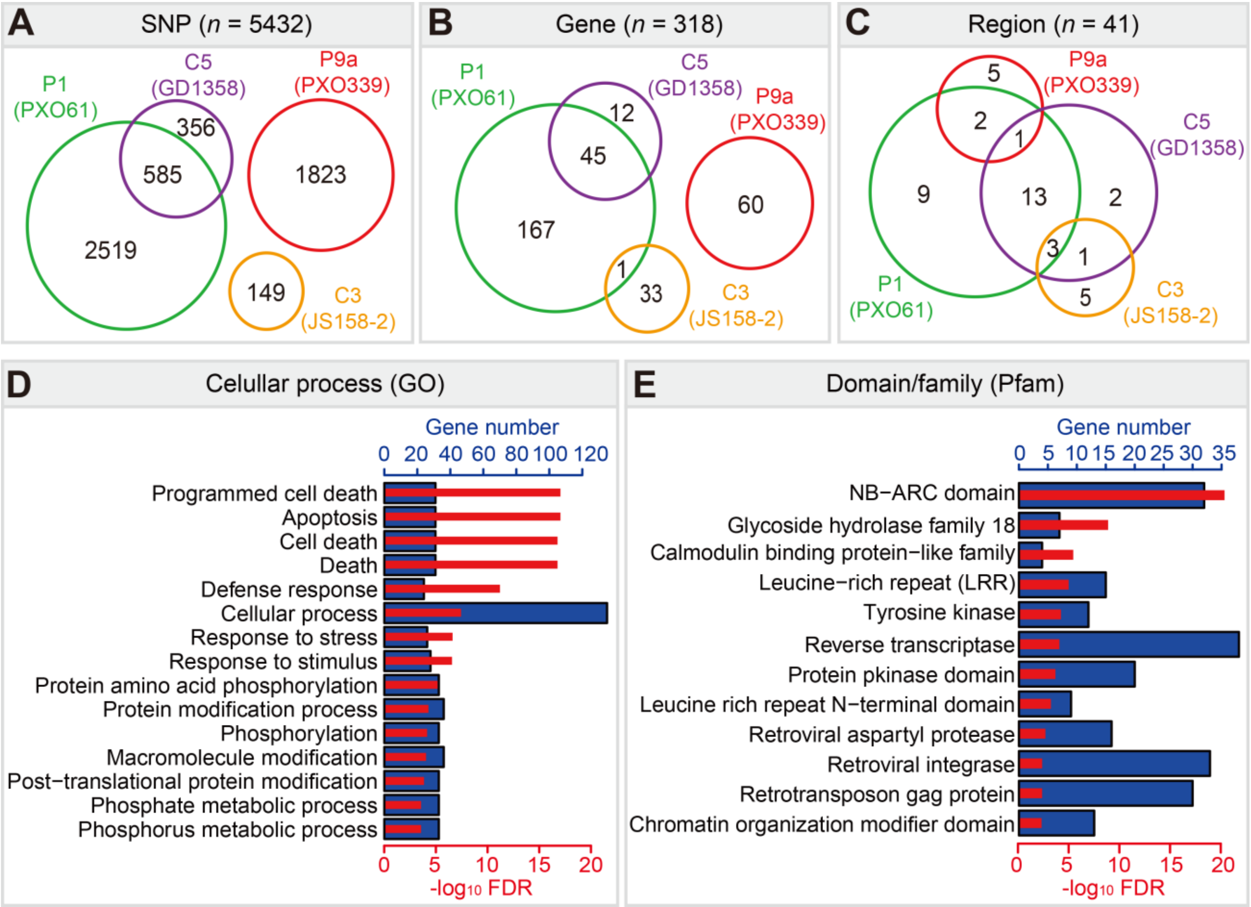

Supplemental Figure S6. Candidate QR-genes to bacterial blight identified in the genome-wide association analyses. (Supports Figure 3)

(**A-C**) Overlapping of detected SNPs (**A**), genes (**B**) and regions (**C**) associated with resistance to *Xoo* races P1, C3, C5 and P9a. (**D**) Enriched GO terms (FDR < 0.05) of the 318 identified rice QR-genes sorted by FDR (FDR < 0.05). (**E**) Enriched Pfam domains/families sorted by FDR (FDR < 0.05).

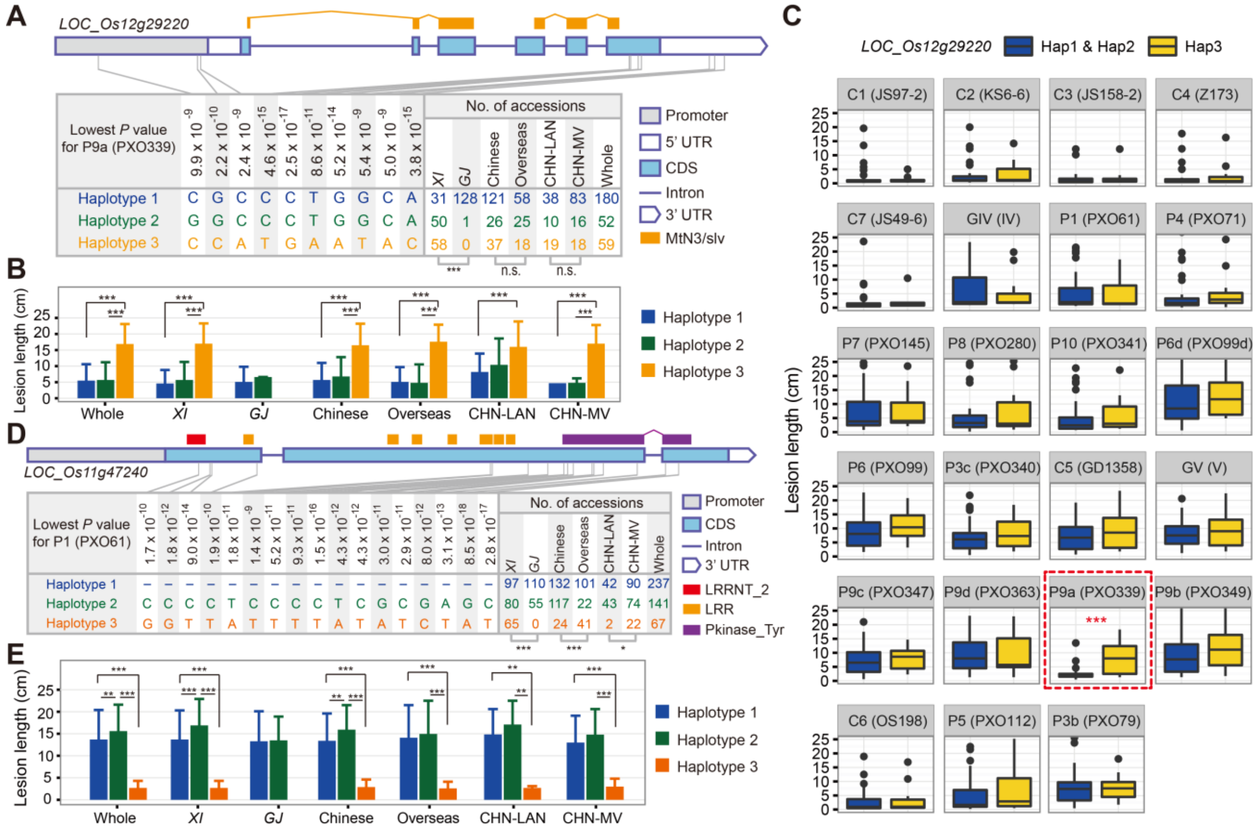

Supplemental Figure S7. Haplotype analysis of *xa25* (*LOC_Os12g29220*) and *Xa26* homolog (*LOC_Os11g47240*). (Supports Figure 3)

(**A**) Haplotypes of *xa25* (*LOC_Os12g29220*) in 291 accessions (rare haplotypes with less than five accessions were not shown) using 10 significant SNPs in the 1 kb upstream promoter region, 3'UTR, and nonsynonymous SNPs in the coding regions in which chi-squared tests were used to compare haplotype frequencies among subpopulations with '***' representing $P < 0.001$ and 'n.s.' representing no significant difference. (**B**) Comparisons of mean lesion lengths between different haplotypes of *xa25* against *Xoo* race P9a in different rice subpopulations based on one-way ANOVA followed by Tukey's HSD post-hoc tests. Data are presented as mean ± standard deviation (*n* = the number of accessions in corresponding subpopulation in [**A**]) (*** $P < 0.001$, 'n.s.' no significant difference). (**C**) Comparisons of mean lesion lengths against 23 *Xoo* strains between haplotypes 1&2 and haplotype 3 of *xa25* (*LOC_Os12g29220*). Significant difference was only detected for *Xoo* race P9a using the *t*-test (*** $P < 0.001$). Boxplots represent the interquartile range, the thick line in the middle of each box represents the median, and the whiskers represent 1.5 times the interquartile range. The data are based on two replications. (**D**) Haplotypes of *Xa26* homolog (*LOC_Os11g47240*) in 445 accessions using 16 nonsynonymous SNPs in the coding regions and 1 stop-gained significant SNP in which chi-squared tests were used to compare haplotype frequencies among subpopulations. Sequences corresponding to the PFAM protein domains were also illustrated: LRRNT_2 (leucine-rich repeat N terminal domain), LRR (leucine-rich repeat) and Pkinase_Tyr (tyrosine kinase). '–' represent missing reads. (**E**) Mean lesion lengths (caused by *Xoo* race P1) vs. haplotypes of *LOC_Os11g47240* in subpopulations. One-way ANOVA followed by Tukey's HSD post-hoc tests were used to detect the major effects. Data are presented as mean ± standard deviation (*n* = the number of accessions in corresponding subpopulation in [**D**]) (* $P < 0.05$, ** $P < 0.01$, *** $P < 0.001$).

```
WYG20 MRASLSHTGISPSFLPTLFFSPHYPLNPPTPSPPLLTSLIASNSLSPSTQLLTSHTYPILATAAAISGDSQSGIGG
JK143 MRASLSHTGISPSFLPTLFFSPHYPLNPPTPSPPLLTSLIASNSLSPSTQLLTSHTYPILATAAAISGDSQSGIGG
JK150 MRASLSHTGISPSFLPTLFFSPHYPLNPPTPSPPLLTSLIASNSLSPSTQLLTSHTYPILATAAAISGDSQSGIGG
JK151 MRASLSHTGISPSFLPTLFFSPHYPLNPPTPSPPLLTSLIASNSLSPSTQLLTSHTYPILATAAAISGDSQSGIGG

WYG20 VDEGDGSLGQWRHQRGQQQFPGNDGGGSNVDGIDEGQVCNRQTPASGSVADNRSDDSRTDNNDDSGGGESNVGGID
JK143 VDEGDGSLGQLAASARAATVSWQRRWREQRRWHRRGPGLQPPDSSERIRCRQPQRRQQDGQQR*
JK150 VDEGDGSLGHGGISEGSNSFLATTVAGATSMASTRARSATARLQRADPLPTTAATTAGRTTTMIVAAARATSAALT
JK151 ISEGSNSFLATTVAGATSMASTRARSATARLQRADPLPTTAATTAGRTTTMIVAAARATSAALTKAGCTTTGLEQV

WYG20 EGRMHHHRARASGSVADDCGDDGGGDGRGDDSDGGRCNDDGRQWLLD*
JK143
JK150 KAGCTTTGLEQVDPLLTTAGTTVAAMGGATIAMAGGATMMAGSGFWI
JK151 DPLLTTAGTTVAAMGGATIAMAGGATMMAGSGFWI
```

Supplemental Figure S8. Comparison of the predicted proteins in wild type Wuyugeng20 (WYG20) and three knockout mutants of *LOC_Os11g46890*. (Supports Figure 4)

JK143, JK150, and JK151 were T_1 mutants of *LOC_Os11g46890* using CRISPR/Cas9 system.

A

Gene models	Promoter					5'UTR			Exon1			
Position (bp)	28005114	28005262	28005304	28005310	28005367	28005704	28006345	28006348	28006410	28006425	28006599	28006602
Lowest *P* value for P1 (PXO61)	8.2E-14	1.4E-12	2.6E-10	9.8E-11	2.2E-11	3.5E-12	3.4E-12	2.0E-12	6.4E-13	1.8E-13	3.0E-11	8.6E-11
Lowest *P* value for C5 (GD1358)	4.9E-22	5.8E-22	3.7E-18	2.5E-18	1.2E-17	6.7E-23	5.9E-19	1.9E-20	3.7E-21	8.1E-21	5.0E-19	1.7E-19
Haplotype1	C	G	C	C	T	T	T	T	G	T	C	T
Haplotype2	C	G	C	C	T	T	T	T	G	T	C	T
Haplotype3	T	A	A	T	A	C	C	C	A	C	A	A

Gene models	Exon4																	
Position (bp)	28007508	28007601	28007607	28007611	28007713	28007716	28007724	28007781	28007787	28007850	28007971	28008027	28008056	28008150	28008292	28008310	28008366	28008377
Lowest *P* value for P1 (PXO61)	7.9E-13	8.8E-13	4.5E-13	4.8E-13	1.9E-13	8.1E-13	2.0E-13	1.5E-13	2.9E-13	4.0E-13	1.2E-10	3.9E-13	2.4E-14	4.2E-14	4.8E-11	1.0E-11	3.0E-12	6.3E-13
Lowest *P* value for C5 (GD1358)	1.3E-21	6.6E-20	4.3E-19	4.0E-20	3.3E-22	6.9E-22	3.1E-22	2.1E-20	4.0E-20	3.9E-21	4.1E-17	8.1E-19	2.2E-21	1.5E-20	2.6E-18	7.4E-18	1.8E-18	3.7E-20
Haplotype1	G	G	G	A	A	C	A	C	A	C	G	T	T	C	A	A	C	G
Haplotype2	G	G	G	A	A	C	A	C	A	C	G	T	T	C	A	A	C	G
Haplotype3	A	A	C	G	G	T	G	G	G	T	A	G	A	G	T	G	G	T

Gene models	Exon6								
Position (bp)	28009122	28009262	28009303	28009319	28009330	28009481	28009546	28009589	28009751
Lowest *P* value for P1 (PXO61)	3.1E-12	2.7E-11	2.8E-10	3.1E-11	4.2E-12	1.6E-11	4.8E-12	2.0E-13	1.7E-08
Lowest *P* value for C5 (GD1358)	1.5E-20	3.7E-19	6.9E-18	1.1E-18	2.5E-20	3.4E-20	3.7E-18	3.1E-20	–
Haplotype1	T	G	A	C	A	G	A	C	C
Haplotype2	T	G	A	C	A	G	A	C	T
Haplotype3	G	A	T	A	G	A	G	T	C

Gene models	Exon7															
Position (bp)	28009976	28010120	28010200	28010237	28010326	28010452	28010462	28010492	28010642	28010717	28010725	28010804	28011043	28011118	28011120	28011772
Lowest *P* value for P1 (PXO61)	5.8E-14	7.4E-13	3.5E-14	2.9E-14	2.0E-14	9.1E-13	7.8E-13	1.4E-12	3.6E-12	1.4E-12	1.5E-12	1.4E-12	5.4E-14	1.3E-09	2.3E-13	2.4E-13
Lowest *P* value for C5 (GD1358)	9.6E-21	1.2E-20	3.6E-20	7.8E-20	6.9E-21	3.3E-19	1.9E-20	3.0E-20	2.4E-21	1.9E-20	1.6E-20	7.1E-19	1.5E-19	4.1E-15	2.3E-19	3.7E-21
Haplotype1	T	A	A	C	C	G	T	G	A	G	G	A	G	A	A	G
Haplotype2	T	A	A	C	C	G	T	G	A	G	G	A	G	A	A	G
Haplotype3	C	G	G	T	G	A	C	A	G	A	A	G	T	C	C	T

Gene models	Exon8	Exon9		No. of accessions						
Position (bp)	28012384	28012559	28012572							
Lowest *P* value for P1 (PXO61)	6.0E-11	7.0E-09	1.6E-09	*XI*	*GJ*	Chinese	Overseas	CHN-LAN	CHN-MV	Whole
Lowest *P* value for C5 (GD1358)	1.9E-16	1.5E-11	–							
Haplotype1	A	G	C	262	169	311	162	104	207	479
Haplotype2	A	G	A	77	0	53	23	2	51	78
Haplotype3	G	C	C	10	11	12	9	2	10	22

XI vs. *GJ*: ***; Chinese vs. Overseas: n.s.; CHN-LAN vs. CHN-MV: ***

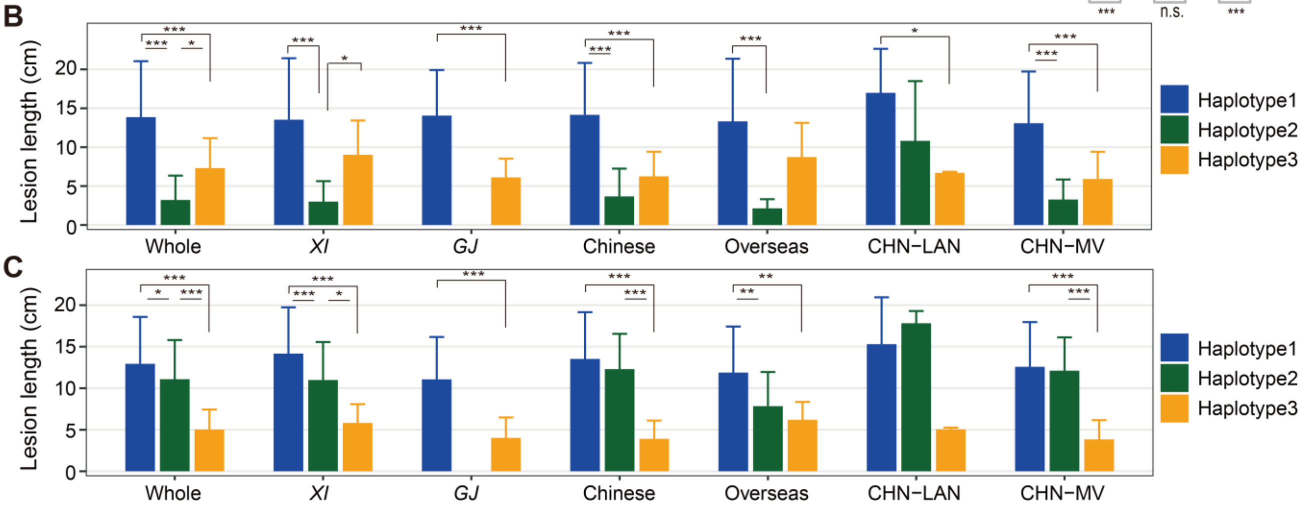

Supplemental Figure S9. Haplotype analysis of *LOC_Os11g46250*. (Supports Figure 3)
(**A**) Haplotypes in 550 accessions using 58 significant SNPs. Chi-square tests were used to compare haplotype frequencies among subpopulations, with '***' representing $P < 0.001$ and 'n.s.' representing no significant difference. (**B**) Mean lesion lengths (caused by *Xoo* race P1) vs. haplotypes in subpopulations. (**C**) Mean lesion lengths (caused by *Xoo* race C5) vs. haplotypes in subpopulations. (**B**) and (**C**) One-way ANOVA followed by Tukey's HSD post-hoc tests were used to detect the major effects. Data are presented as mean ± standard deviation (n = the number of accessions in corresponding subpopulation in [**A**]), with *, ** and *** indicating $P < 0.05$, 0.01 and 0.001, respectively. *XI*, *Xian/indica*; *GJ*, *Geng/japonica*; CHN-LAN, Chinese landraces; CHN-MV, Chinese modern varieties.

Accession ID	Chr4_18654708	Chr8_21203219	Chr11_27996648	Chr2_28599352	Chr5_6202644	Chr11_25667164	Chr12_13459472	Chr3_14441923	Lesion length (cm)
CX380	1	1	1	1	1	1	1	1	0.73
CX353	1	1	1	1	1	1	1	1	1.02
CX190	1	1	1	1	1	1	1	1	1.21
CX324	1	1	1	1	1	1	1	1	1.29
CX355	1	1	1	1	1	1	1	1	1.29
B037	1	1	1	1	1	1	1	1	1.33
YB24_1	1	1	1	1	–	–	–	–	1.35
YB11_1	1	1	1	1	–	–	1	1	1.36
CX350	1	1	1	1	1	1	1	1	1.73
CX311	1	1	1	1	1	1	1	1	1.74
CX326	1	1	1	1	1	1	1	1	1.89
YB17_1	1	1	1	1	1	–	1	1	1.98
CX294	1	1	1	1	1	1	1	–	2.01
YB10_1	1	1	1	1	1	–	1	1	2.03
CX112	1	1	1	1	1	1	1	1	2.13
CX313	1	1	1	1	1	1	1	1	2.25
B091	1	1	1	–	–	–	1	1	2.39
CX185	1	1	1	1	1	1	1	1	2.48
CX11	1	1	1	1	1	1	1	1	2.74
CX369	0	0	0	0	–	0	0	–	0.78
CX126	0	0	0	0	–	–	–	–	0.91
B168	0	0	0	0	1	–	0	–	1.01
CX134	0	0	0	0	0	0	–	–	1.17
CX96	0	0	0	0	–	–	–	–	1.48
CX335	0	0	0	–	–	–	–	–	1.99
B039	0	0	0	0	0	–	–	0	2.23
CX197	0	0	0	–	–	–	–	–	2.31
CX357	0	0	0	–	0	1	0	–	2.33
CX545	0	0	0	0	0	–	–	0	2.77
CX56	0	0	0	0	0	0	0	0	2.82

Supplemental Figure S10. The graphical genotype showing coexistence of the resistance alleles at eight QR-loci jointly determining high-level resistance to SV *Xoo* race C5. (Supports Figure 3)

The green background, the high-level resistance of 19 accessions resulted from the coexistence of resistance alleles at all eight loci; The blue background, the resistance of the remaining 11 accessions appeared to under different genetic control. ‘0’, ‘1’, and ‘ – ’ in the cells represent the susceptibility alleles, resistance alleles, and missing reads, respectively.

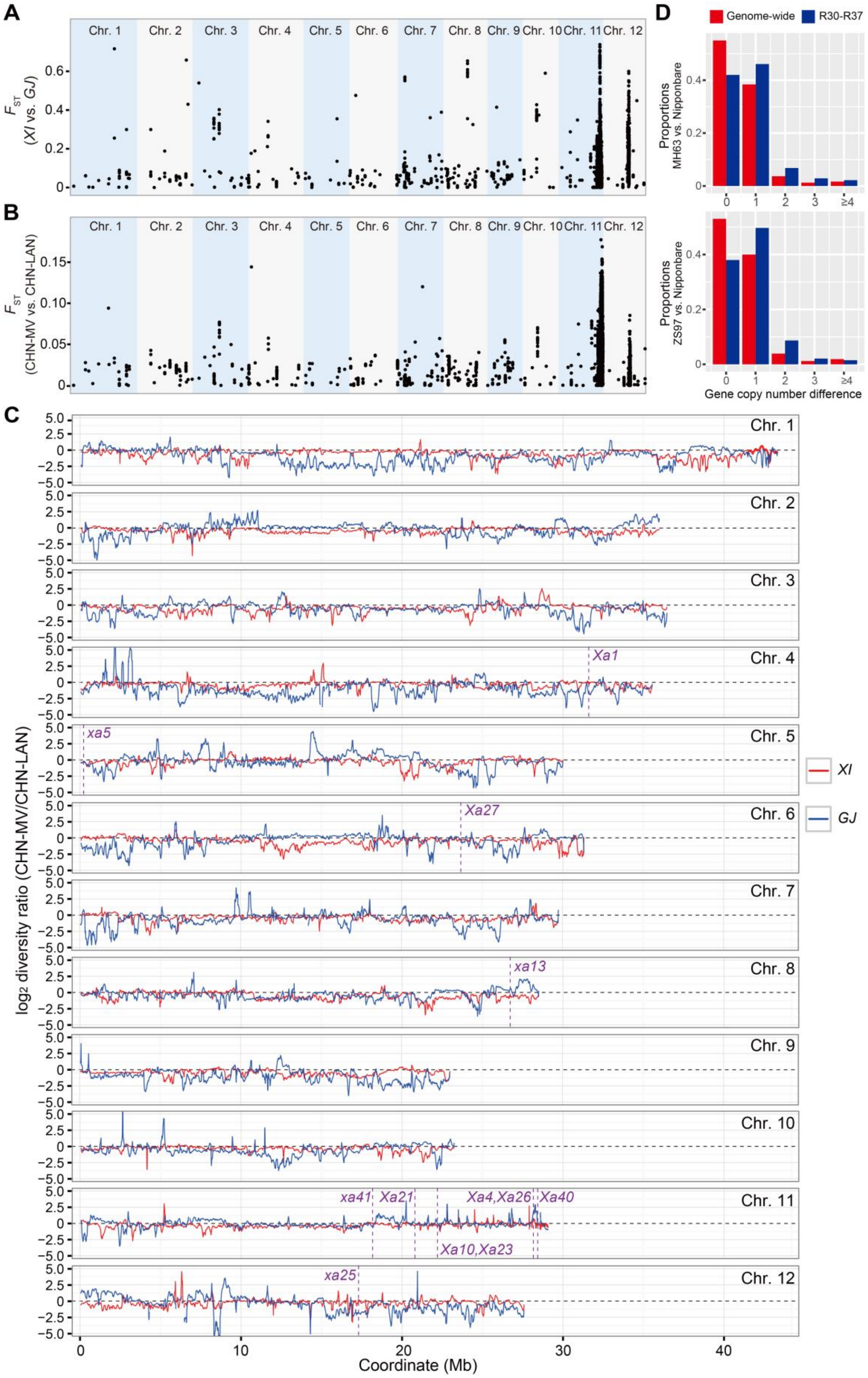

Supplemental Figure S11. Extended and detailed differences in frequencies of resistance alleles, nucleotide diversity, and copy number of the rice QR-genes. (Supports Figures 5 and 6).

(**A**) Differences in frequencies of the resistance alleles at 5,432 significant SNPs between *Xian/indica* (*XI*) accessions (*n* = 419) and *Geng/japonica* (*GJ*) accessions (*n* = 219) measured by F_{ST} statistics. (**B**) Differences in frequencies of the resistance alleles between Chinese modern varieties (CHN-MV) (*n* = 316 accessions) and Chinese landraces (CHN-LAN) (*n* = 135 accessions) measured by F_{ST} statistics. (**C**) The nucleotide diversity of QR-genes. The y-axis shows the nucleotide diversity ratio in log2 scale and the x-axis shows the coordinate of each chromosome. The red line shows the diversity ratio of Chinese *XI* modern varieties (*n* = 188) and Chinese *XI* landraces (*n* = 78) (CHN-MV-*XI*/CHN-LAN-*XI*) and the blue line shows the diversity ratio of Chinese *GJ* modern varieties (*n* = 107) and Chinese *GJ* landraces (*n* = 51) (CHN-MV-*GJ*/CHN-LAN-*GJ*). Coordinates of known *Xa*/*xa* genes are displayed with purple dashed lines. (**D**) Copy number difference of genes within R30-R37. The upper panel shows copy number difference calculated by comparing Nipponbare and Minghui63 genes and the lower panel shows copy number difference calculated by comparing Nipponbare and Zhenshan97 genes. Gene copies were identified with blastp search. Genes with E-value < 1e-5 and identity coverage (IC) > 0.6 were considered as copies. Identity coverage was calculated as IC = 2 × (number of identical residues)/(sum of lengths of the two proteins). For both two comparisons between Nipponbare and Minghui63 and between Nipponbare and Zhenshan97, R30-R37 genes showed more copy numbers (Wilcoxon rank-sum tests, $P < 0.001$).

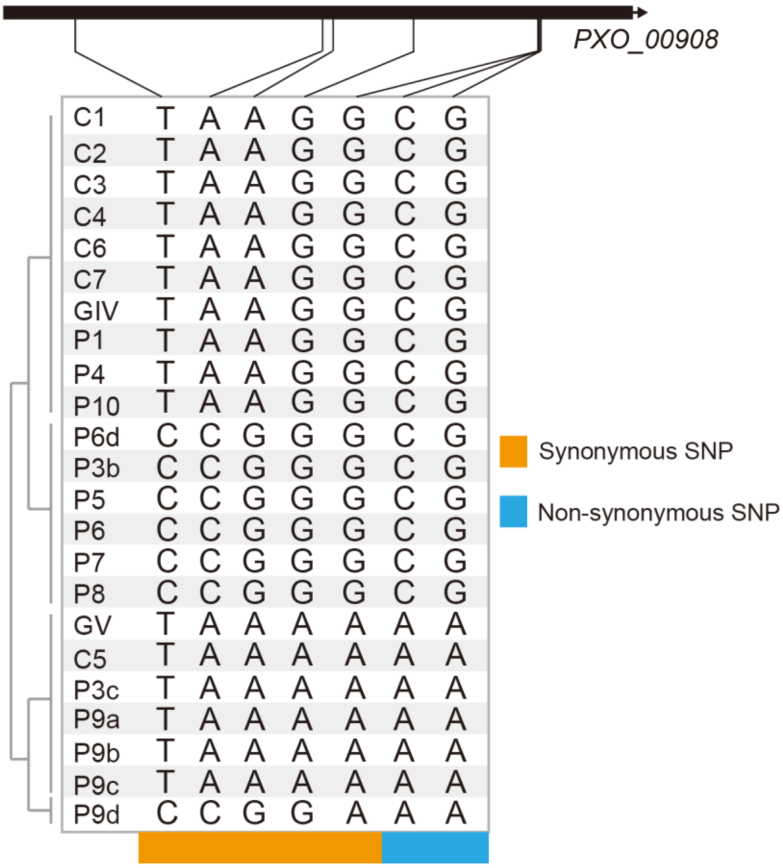

Supplemental Figure S12. The SNP distribution in NADH dehydrogenase gene (*PXO_00908*) across the 23 *Xoo* strains. (Supports Supplemental Table S2)
Five synonymous and two nonsynonymous SNPs are labeled with orange and blue, respectively.

Supplemental Table S1. Summary of SNPs in the 48 known virulence-related genes previously reported in *Xoo* .

Gene ID	Symbol	Gene annotation	Gene length (bp)	SNP	Synonymous	Non-synonymous	SNP density	Hypergeometric test *P* value (SNP_density vs. background=0.0058)
PXO_00567	***talC5a***	***TAL effector AvrBs3/PthA***	***2433***	***55***	***25***	***30***	***0.0226***	***0.0000***
PXO_03417	***hrpF***	***HrpF/NolX/HrpK***	***2409***	***49***	***26***	***23***	***0.0203***	***0.0000***
PXO_05714	***talC9b***	***TAL effector AvrBs3/PthA***	***4452***	***47***	***23***	***24***	***0.0106***	***0.0000***
PXO_00572	*pthXo6*	*TAL effector AvrBs3/PthA*	*3144*	*44*	*13*	*31*	*0.0140*	*0.0000*
PXO_03922	***pthXo7***	***TAL effector AvrBs3/PthA***	***3114***	***40***	***20***	***20***	***0.0128***	***0.0000***
PXO_00546	*talC6a*	*TAL effector AvrBs3/PthA*	*2910*	*40*	*16*	*24*	*0.0137*	*0.0000*
PXO_02172	*talC9a*	*TAL effector AvrBs3/PthA*	*3261*	*38*	*14*	*24*	*0.0117*	*0.0000*
PXO_00505	*talC3b*	*TAL effector AvrBs3/PthA*	*1962*	*35*	*17*	*18*	*0.0178*	*0.0000*
PXO_00227	***pthXo1***	***TAL effector AvrBs3/PthA***	***3804***	***34***	***12***	***22***	***0.0089***	***0.0144***
PXO_02269	*talC9d*	*TAL effector AvrBs3/PthA*	*2205*	*32*	*15*	*17*	*0.0145*	*0.0000*
PXO_00318	talC4	TAL effector AvrBs3/PthA	2436	26	11	15	0.0107	0.0024
PXO_05609	*talC6b*	*TAL effector AvrBs3/PthA*	*3054*	*21*	*6*	*15*	*0.0069*	*0.5039*
PXO_05718	*avrXa27*	*TAL effector AvrBs3/PthA*	*2607*	*19*	*3*	*16*	*0.0073*	*0.3812*
PXO_03420	hpaF	HpaF leucine rich hrp associated protein	1437	17	6	11	0.0118	0.0045
PXO_00223	talC2A	TAL effector AvrBs3/PthA	3207	17	2	15	0.0053	0.8010
PXO_02272	talC9e	TAL effector AvrBs3/PthA	3324	12	4	8	0.0036	0.1223
PXO_03702	*xopX*	*XopX effector protein*	*1911*	*11*	*4*	*7*	*0.0058*	*1.0000*
PXO_03330	*AvrBs2*	*avirulence protein AvrBs2*	*1983*	*9*	*5*	*4*	*0.0045*	*0.5559*
PXO_01578	*hrpA*	*ATP-dependent helicase HrpA*	*4122*	*8*	*2*	*6*	*0.0019*	*0.0016*
PXO_00511	*talC3a*	*TAL effector AvrBs3/PthA*	*1872*	*7*	*3*	*4*	*0.0037*	*0.3080*
PXO_02760		XopN effector	2205	7	2	5	0.0032	0.1387
PXO_01951	*hrpG*	*HrpG protein*	*792*	*4*	*2*	*2*	*0.0051*	*0.9667*
PXO_01953	*hrpX*	*HrpX/HrpB*	*1356*	*3*	*2*	*1*	*0.0022*	*0.1190*
PXO_03398	hrpB4	type III hypothetical protein HrpB4	573	3	2	1	0.0052	1.0000
PXO_03402	hrcU	type III secretion system protein HrcU	1080	2	1	1	0.0019	0.1319
PXO_03405	hrpD1	type III secretion apparatus protein, YscQ/HrcQ family	822	2	1	1	0.0024	0.2983
PXO_03901		XopQ effector	1188	2	1	1	0.0017	0.0938
PXO_03396	*hrcN*	*type III secretion system ATPase*	*1329*	*8*	*7*	*1*	*0.0060*	*1.0000*
PXO_03413	*xopF1*	*XopF1 effector*	*1704*	*8*	*7*	*1*	*0.0047*	*0.6610*
PXO_03399	*hrcJ*	*type III secretion apparatus lipoprotein, YscJ/HrcJ family*	*741*	*2*	*2*	*0*	*0.0027*	*0.3853*
PXO_03409	*hrpD5*	*protein HrpD5*	*759*	*2*	*2*	*0*	*0.0026*	*0.3640*
PXO_03392	hpa1	protein Hpa1	420	1	1	0	0.0024	0.5483
PXO_03394	epaR	type III secretion apparatus protein SpaR/YscT/HrcT	831	1	1	0	0.0012	0.1297
PXO_03397		type III secretion system protein HrpB	702	1	1	0	0.0014	0.2017
PXO_03401		type III hypothetical protein HrpB1/HrpK	426	1	1	0	0.0023	0.5364
PXO_03406	epaP	type III secretion system protein	645	1	1	0	0.0016	0.2457

PXO_03408	*hpaA*	*protein HpaA*	*828*	*1*	*1*	*0*	*0.0012*	*0.1310*
PXO_03411	hrpE	HrpE type III pilin	159	1	1	0	0.0063	1.0000
PXO_03412	hpaB	protein HpaB	192	1	1	0	0.0052	1.0000
PXO_01144		histone acetyltransferase HPA2-like acetyltransferase	495	0	0	0	0.0000	0.1608
PXO_03391	Hpa2	protein Hpa2	327	0	0	0	0.0000	0.3095
PXO_03393	YscC/HrcC	HrpA type III secretion outer membrane pore, YscC/HrcC family	1776	0	0	0	0.0000	0.0022
PXO_03395	SpaR/YscT/HrcT	type III hypothetical protein HrpB7	510	0	0	0	0.0000	0.1520
PXO_03400	hrpB2	type III hypothetical protein HrpB2	393	0	0	0	0.0000	0.2375
PXO_03410	hrpD6	protein HrpD6	243	0	0	0	0.0000	0.4428
PXO_05633	talC7A	TAL effector AvrBs3/PthA	2715	0	0	0	0.0000	0.0001
PXO_06229	talC8a	TAL effector AvrBs3/PthA	2715	0	0	0	0.0000	0.0001
PXO_06234	talC8b	TAL effector AvrBs3/PthA	2952	0	0	0	0.0000	0.0001

The black bold genes are associated with virulence detected by *Xoo* GWAS (Supplemental Table S2). The italic genes are identified in the significant cross-species pairwise interactions in Supplemental Data Set S8.

Supplemental Table S2. Candidate SNPs associated with *Xoo* virulence (lesion length) detected by GWAS.

SNP position (bp)	Gene ID	Symbol	Gene length (bp)	All SNP	Synonymous	Non-synonymous	SNP density	Hypergeometric test *P* value (SNP_density vs. background=0.0058)	CAS[a]	Times of detection	Minimum *P* value	Description	Virulence-associated[b]	Within or outside of the recombination hotspot
21118	*PXO_03467*	---	2139	1	1	0	0.000	0.002	0.895	6	1.53E-05	TonB-dependent outer membrane Receptor	Highly likely	outside
58633	*PXO_03417*	*hrpF*	2409	49	26	23	0.020	0.000	0.928	22	1.00E-05	hypersensitive reaction and pathogenicity, belong to type III secretion system	Yes	outside
58716									0.928	22	1.00E-05			
58753									0.928	22	1.00E-05			
58813									0.928	22	1.00E-05			
58848									0.928	22	1.00E-05			
58896									0.928	22	1.00E-05			
59433									0.928	22	1.00E-05			
59496									0.928	22	1.00E-05			
59531									0.928	22	1.00E-05			
59820									0.928	22	1.00E-05			
60123									0.928	22	1.00E-05			
60240									0.928	22	1.00E-05			
60369									1.420	26	4.30E-06			
60515									0.928	22	1.00E-05			
60564									0.928	22	1.00E-05			
219037	*PXO_03644*	---	2721	76	54	22	0.028	0.000	1.636	15	1.16E-05	Rhs element Vgr protein, belong to type VI secretion system	Highly likely	outside
220755	*PXO_03643*	---	2145	115	66	49	0.054	0.000	0.464	13	2.95E-05	hypothetical protein,linked with *PXO_03644*	Possibly not	outside
227397	*PXO_03638*	---	1677	102	74	28	0.061	0.000	1.779	17	6.18E-06	hypothetical protein,linked with *PXO_03644*	Possibly not	outside
227408									0.820	7	6.02E-05			
227414									0.382	2	9.55E-05			
560092	*PXO_03922*	*pthXo7*	3114	40	20	20	0.013	0.000	0.339	2	1.48E-05	TAL effector AvrBs3/PthA	Yes	outside
560678									0.339	2	1.48E-05			
560682									0.339	2	1.48E-05			
565181	*PXO_03928*	---	546	2	2	0	0.004	0.707	0.300	5	6.64E-05	arabinogalactan endo-1,4-beta-galactosidase, linked with *PXO_03922*	Possibly not	outside
826130	*PXO_04183*	---	711	3	2	1	0.004	0.758	0.300	5	6.64E-05	hypothetical protein, linked with *PXO_03922*	Possibly not	outside
859626	*PXO_04151*	---	1443	25	23	2	0.017	0.000	0.386	2	1.56E-05	major extracellular endoglucanase,cellulase	Highly likely	outside
859632									0.386	2	1.56E-05			
1322914	*PXO_04700*	---	2940	83	51	32	0.028	0.000	0.360	7	7.25E-05	Rhs element Vgr protein, belong to type VI secretion system	Highly likely	outside
1326846	*PXO_04701*	---	2928	73	42	31	0.025	0.000	0.398	2	4.92E-05	DNA repair ATPase, linked with *PXO_04700*	Possibly not	outside
1327811	*PXO_04702*	---	960	31	27	4	0.032	0.000	1.668	18	9.73E-07	lipoprotein, sulfatase-modifying factor 1	Highly likely	outside
1339477	*PXO_04712*	---	1011	20	16	4	0.020	0.000	1.319	4	3.86E-05	Rhs element Vgr protein, belong to type VI secretion system	Highly likely	outside
1464138	*PXO_04839*	---	1098	24	11	13	0.022	0.000	0.469	14	4.07E-05	efflux RND transporter periplasmic adaptor subunit,linked with *PXO_04840*	Possibly not	outside
1464205	*PXO_04840*	---	3057	24	7	17	0.008	0.169	0.469	14	4.07E-05	acriflavin resistance protein	Highly likely	outside
1464481									0.469	14	4.07E-05			
1558675	*PXO_00291*	---	1239	36	24	12	0.029	0.000	0.464	13	2.95E-05	RHS Repeat family	Unknown	outside
1581643	*PXO_00274*	---	1968	88	53	35	0.045	0.000	0.386	2	9.07E-05	hypothetical protein, linked with *PXO_00272*	Unknown	within
1583352	*PXO_00272*	---	2835	215	142	73	0.076	0.000	0.772	3	3.45E-05	hypothetical protein	Unknown	within
1583354									0.772	3	3.45E-05			
1583355									0.772	3	3.45E-05			
1583357									0.772	3	3.45E-05			
1583358									0.772	3	3.45E-05			
1583762									0.300	5	6.64E-05			
1646734	*PXO_00227*	*pthXo1*	3804	34	12	22	0.009	0.015	0.300	5	6.64E-05	TAL effector AvrBs3/PthA	Yes	within
1646748									0.300	5	6.64E-05			
1647463									0.300	5	6.64E-05			
1742977	*PXO_00124*	---	258	4	2	2	0.016	0.100	0.339	2	1.69E-05	ferredoxin	Highly likely	within
1869341	*PXO_00502*	---	2763	74	46	28	0.027	0.000	0.3	5	6.64E-05	Rhs element Vgr protein, belong to type VI secretion system	Highly likely	within
1894180	*PXO_00486*	---	996	4	3	1	0.004	0.594	0.300	5	6.64E-05	LacI family transcriptional regulator, belongs to xenobiotic response family	Highly likely	outside
2008253	*PXO_00394*	---	1377	5	2	3	0.004	0.378	0.3	5	6.64E-05	ATP-dependent RNA helicase DbpA, contain DEAD-box	Unknown	outside
2362111	*PXO_00567*	*talC5a*	2433	55	25	30	0.023	0.000	0.979	8	6.59E-07	TAL effector AvrBs/FPthA	Yes	outside
2397669	*PXO_00833*	*hisD*	1296	1	1	0	0.001	0.028	0.3	5	6.64E-05	histidinol dehydrogenase	Unknown	outside
2458448	*PXO_00908*	---	1413	7	5	2	0.005	0.808	0.928	22	1.00E-05	NADH dehydrogenase	Unknown	outside
2458453									0.928	22	1.00E-05			
2458455									0.928	22	1.00E-05			
2458845	*PXO_00907*	---	2439	12	9	3	0.005	0.661	0.928	22	1.00E-05	hypothetical protein, linked with *PXO_00908*	Possibly not	outside
2459017									0.928	22	1.00E-05			
2459095									0.928	22	1.00E-05			
2459130									0.928	22	1.00E-05			
2459292									0.928	22	1.00E-05			
2459922									0.928	22	1.00E-05			
2943290	*PXO_01233*	*metH*	1140	8	4	4	0.007	0.729	0.300	5	6.64E-05	methionine synthase, linked with *PXO_01764*	Possibly not	outside
3064114	*PXO_05648*	*dnaE*	3591	9	7	2	0.003	0.013	0.3	5	6.64E-05	DNA polymerase III subunit alpha,linked with *PXO_01764*	Possibly not	outside
3121994	*PXO_01764*	---	1212	1	0	1	0.001	0.036	0.300	5	6.64E-05	major facilitator superfamily (MFS) transporter, facilitating the transport across cytoplasmic or internal membranes of various substrates including ions, sugar phosphates, drugs, neurotransmitters, nucleosides, amino acids, and peptides	Highly likely	outside
3245832	*PXO_01645*	*glk*	999	10	5	5	0.010	0.123	0.652	4	1.49E-06	glucokinase	Highly likely	outside
3249971	*PXO_01644*	---	2628	305	209	96	0.116	0.000	0.634	17	3.13E-05	TonB-dependent receptor	Highly likely	outside
3380824	*PXO_01534*	---	2835	164	118	46	0.058	0.000	0.667	2	7.42E-05	hypothetical protein, linked with *PXO_01532*	Unknown	outside
3381418									2.077	2	6.98E-05			

3383169	*PXO_01532*	---	327	7	5	2	0.021	0.001	0.398	2	4.92E-05	hypothetical protein, linked with *PXO_01533*	Unknown	outside
3465283	*PXO_01443*	---	1866	9	4	5	0.005	0.687	0.3	5	6.64E-05	hypothetical protein, putative member protein	Unknown	outside
3849115<br>3849116<br>3849383	*PXO_02058*	---	2499	108	61	47	0.043	0.000	2.233<br>2.233<br>0.386	30<br>30<br>2	7.69E-06<br>7.69E-06<br>9.07E-05	hypothetical protein	Unknown	outside
4054397	*PXO_02227*	*rpoN*	1398	2	1	1	0.001	0.048	0.386	2	9.07E-05	RNA polymerase factor sigma-54, regulating expression of virulence and virnlence-associated genes	Highly likely	outside
4107529	*PXO_05714*	*talC9b*	4452	47	23	24	0.011	0.000	1.376	7	5.77E-05	TAL effector AvrBs3/PthA	Yes	outside
4310531<br>4310532	*PXO_02463*	---	2148	162	92	70	0.075	0.000	0.784<br>0.784	5<br>5	2.06E-05<br>2.06E-05	hypothetical protein,with Pimeloyl-ACP methyl ester	Unknown	outside
4678336	*PXO_03117*	*glmU*	1344	11	7	4	0.008	0.331	0.895	6	1.53E-05	UDP-N-acetylglucosamine pyrophosphorylase	Unknown	outside
4680787	*PXO_03115*	---	534	1	1	0	0.002	0.363	0.3	5	6.64E-05	chorismate mutase	Highly likely	outside
4726257	*PXO_03063*	---	657	8	6	2	0.012	0.058	0.895	6	1.53E-05	two-component response regulator	Highly likely	outside
4744019	*PXO_03046*	---	777	1	1	0	0.001	0.155	0.3	5	6.64E-05	4,5-dopa dioxygenase extradiol, linked with *PXO_03021*	Possibly not	outside
4764572	*PXO_03021*	---	381	5	0	5	0.013	0.122	0.300	5	6.64E-05	regulatory protein, an CheY chemotaxis protein	Highly likely	outside
5118971<br>5119040	*PXO_03275*	---	1029	2	2	0	0.002	0.155	0.996<br>0.564	7<br>5	6.81E-05<br>5.56E-05	quinone oxidoreductase	Highly likely	outside
5124888	*PXO_03270*	---	2472	10	6	4	0.004	0.309	1.871	21	3.30E-06	ATP-dependent RNA helicase	Unknown	outside
5159459	*PXO_03231*	---	1395	3	1	2	0.002	0.106	0.634	17	3.13E-05	hypothetical protein, putative NAD(P)/FAD-binding protein	Unknown	outside

[a]CAS: combined association score. See the Methods section for more details.

[b]Virulence-associated: Yes - known virulence-related genes previously reported in *Xoo* ; Highly likely - with evidence to be virulence associated based on literature on other bacterial species; Possibly not - linked to known or highly possible virulence-related genes, while itself shows no evidence to be virulence-related; Unknown - cannot be explained by a nearby genes with significant SNPs and no/weak evidence to be virulence-related.

Supplemental Table S3. Twenty-three genes highly associated with *Xoo* virulence (lesion length) detected by GWAS.

Gene ID	Symbol	Leading SNPs			Description	Virulence-associated[b]	Evidence
		CAS[a]	Times of detection	Minimum *P* value			
PXO_04702	---	1.668	18	9.70E-07	lipoprotein	Highly likely	(Kovacs-Simon et al., 2011)
PXO_03644	---	1.636	15	1.20E-05	type VI secretion system	Highly likely	(Alteri and Mobley, 2016)
PXO_03417	*hrpF*	1.42	26	4.30E-06	type III secretion system	Yes	
PXO_05714	*talC9b*	1.376	7	5.80E-05	TAL effector	Yes	
PXO_04712	---	1.319	4	3.90E-05	type VI secretion system	Highly likely	(Alteri and Mobley, 2016)
PXO_03275	---	0.996	7	6.80E-05	quinone oxidoreductase	Highly likely	(Ryan et al., 2014)
PXO_00567	*talC5a*	0.979	8	6.60E-07	TAL effector	Yes	
PXO_03467	---	0.895	6	1.50E-05	TonB-dependent outer membrane Receptor	Highly likely	(Ryan et al., 2014)
PXO_03063	---	0.895	6	1.50E-05	two-component response regulator	Highly likely	(Ryan et al., 2014)
PXO_01645	*glk*	0.652	4	1.50E-06	glucokinase	Highly likely	(Wang et al., 2017)
PXO_01644	---	0.634	17	3.10E-05	TonB-dependent receptor	Highly likely	(Ryan et al., 2014)
PXO_04840	---	0.469	14	4.10E-05	acriflavine resistance protein	Highly likely	(Nishino et al., 2006)
PXO_04151	---	0.386	2	1.60E-05	cellulase	Highly likely	(Xia et al., 2016)
PXO_02227	*rpoN*	0.386	2	9.10E-05	RNA polymerase factor sigma-54	Highly likely	(Kazmierczak et al., 2005)
PXO_04700	---	0.36	7	7.30E-05	type VI secretion system	Highly likely	(Alteri and Mobley, 2016)
PXO_03922	*pthXo7*	0.339	2	1.50E-05	TAL effector	Yes	
PXO_00124	---	0.339	2	1.70E-05	ferredoxin	Highly likely	(Grinter et al., 2016)
PXO_00227	*pthXo1*	0.3	5	6.60E-05	TAL effector	Yes	
PXO_00502	---	0.3	5	6.60E-05	type VI secretion system	Highly likely	(Alteri and Mobley, 2016)
PXO_00486	---	0.3	5	6.60E-05	LacI family transcriptional regulator, xenobiotic response family	Highly likely	(Nishino et al., 2006)
PXO_01764	---	0.3	5	6.60E-05	major facilitator superfamily transporter	Highly likely	(Nishino et al., 2006)
PXO_03115	---	0.3	5	6.60E-05	chorismate mutase	Highly likely	(Degrassi et al., 2010)
PXO_03021	---	0.3	5	6.60E-05	CheY chemotaxis protein	Highly likely	(Yao et al., 1997)

[a]CAS: combined association score. See the Methods section for more details.

[b]Virulence-associated: Yes - known virulence-related genes previously reported in *Xoo*; Highly likely - with evidence to be virulence associated based on the literature on other bacterial species.

Supplemental Table S4. Forty-one genomic regions < 300 kb each containing > 5 significant SNPs for resistance to 1-4 *Xoo* races detected in the second set of rice materials.

Region ID	Chr.	Position (bp)	Leading SNP position (bp)	No. of significant SNPs	GWAS panel[a]	*Xoo* races	Minimum *P* value	Selection/differentiation based on Figure 5 and Supplemental Figure S11	Known *Xa*/*xa* genes
R1	1	32274258-32275070	32274685	7	Whole, *XI*, CHN, Overseas, CHN-MV	P9a	7.3E-15		
R2	1	36902819-36920007	36902819	7	Whole, CHN, CHN-MV	C5	3.6E-12		
R3	2	25941893-25976332	25943338	16	Whole, *XI*, Overseas	P1	1.7E-10		
R4	2	32189432-32480516	32189584	9	Whole, *XI*, *GJ*, CHN, Overseas	P1, P9a	3.1E-13	*XI*-*GJ* differentiation	
R5	3	14441416-14441961	14441923	10	Whole, GJ, CHN, CHN-MV	C5,P1	3.3E-18	*XI*-*GJ* differentiation	
R6	3	17879096-17879805	17879116	9	Whole, *XI*, CHN-MV	P1	5.9E-11	Artificial selection	
R7	3	31998146-32000365	32000365	12	CHN, CHN-MV	C5	1.4E-11		
R8	4	579031-583634	579454	17	Whole, *XI*, Overseas, CHN-MV	C5, P1	1.1E-15		
R9	4	18654588-18656689	18654708	23	Whole, *XI*, *GJ*, CHN, Overseas, CHN-MV	C5, P1	4.3E-20		
R10	6	2739748-3029850	3029845	11	Whole, CHN, CHN-MV	C3, C5, P1	8.1E-14		
R11	6	5822478-5823373	5823342	9	Whole, *XI*, Overseas	P9a	1.4E-13		
R12	7	178697-222511	222462	46	Whole, *XI*, CHN, CHN-MV	C5, P1	3.9E-15		
R13	7	4601794-4796704	4601794	9	CHN-MV	C3	7.0E-11		
R14	7	5132570-5313560	5304728	35	CHN-MV	C3	6.9E-11	*XI*-*GJ* differentiation	
R15	7	7718050-7720854	7720662	33	Whole, *XI*, CHN, Overseas, CHN-MV	P1	1.9E-17		
R16	7	26199068-26203533	26202354	8	Whole, *XI*, *GJ*, CHN-MV	C5, P1	1.2E-13		
R17	7	29015054-29252029	29015054	11	Whole, *GJ*, CHN, CHN-MV	C3	1.2E-13		
R18	8	3316885-3480604	3317201	15	Whole, *XI*, CHN, Overseas, CHN-MV	P1, P9a	1.2E-15		
R19	8	3853402-3855713	3854299	34	Whole, *XI*, Overseas	P1	4.6E-17		
R20	8	15994461-16289218	15994464	14	Whole, *GJ*, CHN, CHN-MV	C5, P1, P9a	1.2E-17		
R21	8	21201867-21204241	21203219	13	Whole, *XI*, *GJ*, CHN, Overseas, CHN-MV	C5,P1	1.3E-19		
R22	8	21526854-21531248	21527491	37	Whole, *XI*, CHN	P1	1.2E-11		
R23	9	11360839-11622919	11425437	11	Whole, *XI*, CHN, CHN-MV	C3	2.5E-12		
R24	9	11679069-11763493	11728944	22	Whole, *XI*, CHN	C3	1.6E-10		
R25	9	13505037-13511371	13510784	7	Whole, *XI*	P1	5.6E-13		
R26	10	9709662-9712237	9710084	14	Whole, *XI*, CHN, Overseas, CHN-MV	P1	6.3E-15	Artificial selection, *XI*-*GJ* differentiation	
R27	11	8211304-8357742	8357014	8	Whole, *GJ*, CHN, CHN-MV	C3, C5	4.5E-15		
R28	11	21553084-21554057	21553661	8	Whole, *XI*, Overseas, CHN-MV	P1	3.2E-10		
R29	11	23449282-23450834	23449282	6	Whole, CHN, Overseas, CHN-MV	C5, P1	2.8E-12		

R30	11	25516416-25718643	25656776	125	Whole, *XI* , *GJ* , CHN, Overseas, CHN-MV	C5, P1	1.4E-13	Artificial selection	
R31	11	26106272-26123951	26116101	18	Whole, *XI* , *GJ* , CHN, CHN-MV	P1	2.3E-10		
R32	11	26297057-26502906	26461020	118	Whole, *XI* , *GJ* , CHN, Overseas, CHN-MV	C5, P1	1.7E-17	Artificial selection	
R33	11	27000551-27284653	27035350	225	Whole, *XI* , CHN, Overseas, CHN-MV	C5, P1	6.5E-14	Artificial selection	
R34	11	27381223-27675979	27474872	814	Whole, *XI* , CHN, Overseas, CHN-MV	C3, C5, P1	2.4E-23	Artificial selection, *XI* -*GJ* differentiation	*Xa32* , *Xa35* , *Xa36*
R35	11	27920237-28187166	28091702	782	Whole, *XI* , *GJ* , CHN, Overseas, CHN-MV	C5, P1	2.4E-27	Artificial selection, *XI* -*GJ* differentiation	*Xa40*
R36	11	28212179-28497785	28229125	505	Whole, *XI* , *GJ* , CHN, Overseas, CHN-MV	C5, P1	2.4E-22	Artificial selection, *XI* -*GJ* differentiation	*Xa4, Xa26* , *Xa22*
R37	11	28652407-28796522	28758679	116	Whole, *XI* , CHN, Overseas, CHN-MV	C5, P1	6.5E-16		
R38	12	13216938-13513245	13459472	10	Whole, *XI* , CHN, Overseas, CHN-MV	C3, C5, P1	1.5E-17		
R39	12	16413092-16602641	16600192	57	Whole, *XI* , CHN, Overseas	P9a	1.1E-12	*XI* -*GJ* differentiation	
R40	12	17035491-17312462	17195211	887	Whole, *XI* , CHN, Overseas, CHN-MV	P9a	6.3E-21	*XI* -*GJ* differentiation	*xa25*
R41	12	17324810-17528364	17366478	728	Whole, *XI* , CHN, Overseas, CHN-MV	P9a	8.7E-20	*XI* -*GJ* differentiation	

[a] Whole (*n* = 701 accessions); *XI* , *Xian/indica* (*n* = 419 accessions); *GJ* , *Geng/japonica* (*n* = 219 accessions); CHN, Chinese (*n* = 451 accessions); Overseas (n = 451 accessions); CHN-MV, Chinese modern varieties (*n* = 316 accessions).

Supplemental Table S5. Results of a GO enrichment analysis of the detected rice genes that interacted with *Xoo* virulence-related genes.

GO term	Ontology[a]	Description	Number in input list	Number in BG/Ref	*P* value	FDR
GO:0006468	BP	protein amino acid phosphorylation	26	1570	3.10E-09	5.10E-07
GO:0016310	BP	phosphorylation	26	1695	1.50E-08	1.20E-06
GO:0006464	BP	protein modification process	27	1929	4.80E-08	1.60E-06
GO:0006796	BP	phosphate metabolic process	26	1795	4.60E-08	1.60E-06
GO:0006793	BP	phosphorus metabolic process	26	1795	4.60E-08	1.60E-06
GO:0043687	BP	post-translational protein modification	26	1815	5.70E-08	1.60E-06
GO:0043412	BP	macromolecule modification	27	1979	8.00E-08	1.90E-06
GO:0006915	BP	apoptosis	14	532	1.10E-07	2.00E-06
GO:0012501	BP	programmed cell death	14	532	1.10E-07	2.00E-06
GO:0008219	BP	cell death	14	544	1.40E-07	2.10E-06
GO:0016265	BP	death	14	544	1.40E-07	2.10E-06
GO:0044267	BP	cellular protein metabolic process	29	2877	1.10E-05	1.60E-04
GO:0009987	BP	cellular process	75	12839	1.20E-04	1.50E-03
GO:0006952	BP	defense response	9	452	1.80E-04	2.20E-03
GO:0019538	BP	protein metabolic process	31	4333	2.70E-03	3.00E-02
GO:0044260	BP	cellular macromolecule metabolic process	55	9350	3.40E-03	3.50E-02
GO:0006950	BP	response to stress	10	873	5.10E-03	5.00E-02
GO:0050896	BP	response to stimulus	11	1026	5.40E-03	5.00E-02
GO:0005524	MF	ATP binding	46	3085	5.80E-15	5.10E-13
GO:0032559	MF	adenyl ribonucleotide binding	46	3087	6.00E-15	5.10E-13
GO:0001883	MF	purine nucleoside binding	46	3247	3.90E-14	1.50E-12
GO:0001882	MF	nucleoside binding	46	3257	4.40E-14	1.50E-12
GO:0030554	MF	adenyl nucleotide binding	46	3247	3.90E-14	1.50E-12
GO:0032555	MF	purine ribonucleotide binding	46	3292	6.40E-14	1.60E-12
GO:0032553	MF	ribonucleotide binding	46	3292	6.40E-14	1.60E-12
GO:0017076	MF	purine nucleotide binding	46	3456	3.80E-13	8.10E-12
GO:0000166	MF	nucleotide binding	47	3874	5.20E-12	9.90E-11
GO:0004672	MF	protein kinase activity	26	1575	3.30E-09	5.70E-08
GO:0004674	MF	protein serine/threonine kinase activity	24	1478	2.10E-08	3.00E-07
GO:0016773	MF	phosphotransferase activity, alcohol group as acceptor	26	1726	2.10E-08	3.00E-07
GO:0016301	MF	kinase activity	26	1750	2.80E-08	3.70E-07
GO:0016772	MF	transferase activity, transferring phosphorus-containing groups	43	4781	6.60E-07	8.00E-06
GO:0005488	MF	binding	92	16823	1.70E-05	1.90E-04
GO:0016740	MF	transferase activity	45	6190	1.00E-04	1.10E-03
GO:0004553	MF	hydrolase activity, hydrolyzing O-glycosyl compounds	8	424	6.00E-04	6.00E-03
GO:0016798	MF	hydrolase activity, acting on glycosyl bonds	8	480	1.30E-03	1.20E-02

[a]BP,Biological process; MF, Molecular function.

Supplemental Table S6. Information for vector constructions and primers.

Name	Primer sequences (5'-3')	Purpose
U-F	CTCCGTTTTACCTGTGGAATCG	Construction for CRISPR/Cas9 vector
gR-R	CGGAGGAAAATTCCATCCAC	
U6a-LOC_Os11g46890	CCATTGTCCAAGACTACCGTCggcagccaagccagca	
gRT1-LOC_Os11g46890	ACGGTAGTCTTGGACAATGGgttttagagctagaaat	
U6b-LOC_Os11g46890	CGCTGATGCCGCCATTGTCCaacacaagcggcagc	
gRT2-LOC_Os11g46890	GACAATGGCGGCATCAGCGgttttagagctagaaat	
LOC_Os11g46890-TF	TGCAAAAATCGCTTCCCTAT	Amplification of fragment containing editing sites
LOC_Os11g46890-TR	ATCCAGAAGCCACTGCCGG	
LOC_Os11g46890-SP	ATGCGAGCTTCTCTCTCCCACA	Sequecing the fragment containing editing sites

REFERENCES

Kazmierczak, M.J., Wiedmann, M., and Boor, K.J. (2005). Alternative sigma factors and their roles in bacterial virulence. Microbiol. Mol. Biol. Rev. 69, 527-543.

Kovacs-Simon, A., Titball, R.W., and Michell, S.L. (2011). Lipoproteins of bacterial pathogens. Infect. Immun. 79, 548-561.

Nishino, K., Latifi, T., and Groisman, E.A. (2006). Virulence and drug resistance roles of multidrug efflux systems of Salmonella enterica serovar Typhimurium. Mol. Microbiol. 59, 126-141.

Ryan, A., Kaplan, E., Nebel, J.C., Polycarpou, E., Crescente, V., Lowe, E., Preston, G.M., and Sim, E. (2014). Identification of NAD(P)H quinone oxidoreductase activity in azoreductases from P. aeruginosa: azoreductases and NAD(P)H quinone oxidoreductases belong to the same FMN-dependent superfamily of enzymes. PLoS One 9, e98551.

Wang, J., Guo, J., Wang, S., Zeng, Z., Zheng, D., Yao, X., Yu, H., and Ruan, L. (2017). The global strategy employed by Xanthomonas oryzae pv. oryzae to conquer low-oxygen tension. J. Proteomics 161, 68-77.

Xia, T., Li, Y., Sun, D., Zhuo, T., Fan, X., and Zou, H. (2016). Identification of an extracellular endoglucanase that is required for full virulence in Xanthomonas citri subsp. citri. PLoS One 11, e0151017.

Yao, R., Burr, D.H., and Guerry, P. (1997). CheY-mediated modulation of Campylobacter jejuni virulence. Mol. Microbiol. 23, 1021-1031.
