# Supplementary material for: Reciprocal adaptation of rice and Xanthomonas oryzae pv. oryzae: cross-species 2D GWAS reveals the underlying genetics
Source: Plant Cell. 2021 Jun 2;33(8):2538–61. doi: 10.1093/plcell/koab146 (PMC8408478; doi:10.1093/plcell/koab146)
Supplement: koab146_Supplementary_Data [file koab146_supplementary_data.zip › tpc.01058.2020-s06.pdf]

Supplemental File S1. Sequence alignments and phylogenetic tree files of two *Xoo* genes (*PXO_03417* and *PXO_01644*).

cds.prot.fasta for PXO_03417 (Supports Supplemental Figure S2A)

>PXO99A [gene=hrpF] [protein=HrpF/NolX/HrpK] [protein_id=YP_001911263.1] [location=complement(58563..60971)]
MSLNMLSTGSNPSQLLGTSSNESSSSELFGSDSSNDGSDLPSTMDTIFQQIYLLLAALQA
NTQTSASGDTPANTASGDADTQMSASDWQATQPIEKRTSWPSLGYDFDPKNIKGKDAPPA
LEGSTVTWNDGTLTKSELQIVSTLNAHKDQMPIEYKNLDDKINDPSTPPDLKAALQGLKQ
DPRLFFAIGSQGDGKCGGKVSAQDLWDFSDSHPQVKDLGGKNDEFNPKDIKGSNPPQAAE
GSTVTWNDGQLNQSELEIVSVLDRHKDQVDSLSFDQLDAKINDPSTQPDLKKALKGLQKD
PRLFFAIGSQKDGKCGGKIKAQDLTDFSYYHPQIAEYNDKKAKSYTQNYIASDSPDKTKA
SVMTKSDALREMYRYSDYLPGNLSEDEFAKIVDGDSKTGKCPPQLIAAAQYFRDHPDEWK
EFSGDAGTMSTPDFLQKSTSEMHLTADEQKTLDTINSHQDAFYGDGKELTRDKLDAISKD
NKADPAVKEAATQLASDPLLFGLLNNSITGYKKPHHFFGGGHVVDSGKISQNDFRQFYDH
MSAANKTVNTPATHEASSPDQQKAVADMLMGKDDPPAIKKPKKDVGTFQQGLHEFLKWDS
KILDWMSVGLSALNGIPVIGEIADAAAIALESEAQAAQVVDTAIQGGDMSLALKLAGINM
AGAVVGAVGGPTARIGAKGAAKGVAEVAAKEAAEGAAKGTAKGSAKGAGKTAAERPSAAA
FAKGYVAGSTISKSTEILKKPVMAGLHYEEYQLDKQKDGEIHQKLDNAGGAPVGKQIIPT
GIADNFEGDVRQNLRNVRIRRR*
>C1 locus=Scaffold13:70961:73369:-
MSLNMLSTGSNPSQLLGTSSNESSSSELFGSDSSNDGSDLPSTMDTIFQQIYLLLAALQA
NTQTSASGDTPANTASGDADTQMSASDWQATQPIEKRTSWPSLGYDFDPKNIKGKDAPPA
LEGSTVTWNDGTLTKSELQIVSTLNAHKDQMPIEYKNLDDKINDPSTPPDLKAALQGLKQ
DPRLFFAIGSQGDGKCGGKVSAQDLWDFSDSHPQVKDLGGKNDEFNPKDIKGSNPPQAAE
GSTVTWNDGQLNQSELEIVSVLDRHKDQVDSLSFDQLDAKINDPSTQPDLKKALKGLQKD
PRLFFAIGSQKDGKCGGKIKAQDLTDFSYYHPQIAEYNDKKAKSYTQNYIASDSPDKTKA
SVMTKSDALREMYRYSDYLPGNLSEDEFAKIVDGDSKTGKCPPQLIAAAQYFRDHPDEWK
EFSGDAGTMSTPDFLQKSTSEMHLTADEQKTLDTINSHQDAFYGDGKELTRDKLDAISKD
NKADPAVKEAATQLASDPLLFGLLNNSITGYKKPHHFFGGGHVVDSGKISQNDFRQFYDH
MSAANKTVNTPATHEASSPDQQKAVADMLMGKDDPPAIKKPKKDVGTFQQGLHEFLKWDS
KILDWMSVGLSALNGIPVIGEIADAAAIALESEAQAAQVVDTAIQGGDMSLALKLAGINM
AGAVVGAVGGPTARIGAKGAAKGVAEVAAKEAAEGAAKGTAKGAAKGAGKTAAERPSAAA
FAKGYVAGSTISKSTEILKKPVMAGLHYEEYQLDKQKDGEIHQKLDNAGGAPVGRQIIPT
GIADNFEGDVRQNLRNVRIRRR*
>C2 locus=Scaffold1:71354:73726:-
MSLNMLSTGSNPSQLLGTSSNESSSSELFGSDSSNDGSDLPSTMDTIFQQIYLLLAALQA
NTQTSASGDTPANTASGDADTQMSASDWQATQPIEKRTSWPSLGYDFDPKNIKGKDAPPA
LEGSTVTWNDGTLTKSELQIVSTLNAHKDQMPIEYKNLDDKINDPSTPPDLKAALQGLKQ
DPRLFFAIGSQGDGKCGGKVSAQDLWDFSDSHPQVKDLGGKNDEFNPKDIKGSNPPQAAE
GSTVTWNDGQLNQSELEIVSVLDRHKDQVDSLSFDQLDAKINDPSTQPDLKKALKGLQKD
PRLFFAIGSQKDGKCGGKIKAQDLTDFSYYHPQIAEYNDKKAKSYTQNYIASDSPDKTKA
SVMTKSDALREMYRYSDYLPGNLSEDEFAKIVDGDSKTGKCPPQLIAAAQYFRDHPDEWK

EFSGDAGTMSTPDFLQKSTSEMHLTADEQKTLDTINSHQDAFYGDGKELTRDKLDAISKD
NKADPAVKEAATQLASDPLLFGLLNNSITGYKKPHHFFGGGHVVDSGKISQNDFRQFYDH
MSAANKTVNTPATHEASSPDQQKAVADMLMGKDDPPAIKKPKKDVGTFQQGLHEFLKWDS
KILDWMSVGLSALNGIPVIGEIADAAAIALESEAQAAQVVDTAIQGGDMSLALKLAGINM
AGAVVGAVGGPTARIGAKEAAEGAAKGTAKGAAKGAGKTAAERPSAAAFAKGYVAGSTIS
KSTEILKKPVMAGLHYEEYQLDKQKDGEIHQKLDNAGGAPVGKQIIPTGIADNFEGDVRQ
NLRNVRIRRR*
>C3 locus=Scaffold15:30301:32673:+
MSLNMLSTGSNPSQLLGTSSNESSSSELFGSDSSNDGSDLPSTMDTIFQQIYLLLAALQA
NTQTSASGDTPANTASGDADTQMSASDWQATQPIEKRTSWPSLGYDFDPKNIKGKDAPPA
LEGSTVTWNDGTLTKSELQIVSTLNAHKDQMPIEYKNLDDKINDPSTPPDLKAALQGLKQ
DPRLFFAIGSQGDGKCGGKVSAQDLWDFSDSHPQVKDLGGKNDEFNPKDIKGSNPPQAAE
GSTVTWNDGQLNQSELEIVSVLDRHKDQVDSLSFDQLDAKINDPSTQPDLKKALKGLQKD
PRLFFAIGSQKDGKCGGKIKAQDLTDFSYYHPQIAEYNDKKAKSYTQNYIASDSPDKTKA
SVMTKSDALREMYRYSDYLPGNLSEDEFAKIVDGDSKTGKCPPQLIAAAQYFRDHPDEWK
EFSGDAGTMSTPDFLQKSTSEMHLTADEQKTLDTINSHQDAFYGDGKELTRDKLDAISKD
NKADPAVKEAATQLASDPLLFGLLNNSITGYKKPHHFFGGGHVVDSGKISQNDFRQFYDH
MSAANKTVNTPATHEASSPDQQKAVADMLMGKDDPPAIKKPKKDVGTFQQGLHEFLKWDS
KILDWMSVGLSALNGIPVIGEIADAAAIALESEAQAAQVVDTAIQGGDMSLALKLAGINM
AGAVVGAVGGPTARIGAKEAAEGAAKGTAKGAAKGAGKTAAERPSAAAFAKGYVAGSTIS
KSTEILKKPVMAGLHYEEYQLDKQKDGEIHQKLDNAGGAPVGKQIIPTGIADNFEGDVRQ
NLRNVRIRRR*
>C4 locus=Scaffold3:601414:603786:+
MSLNMLSTGSNPSQLLGTSSNESSSSELFGSDSSNDGSDLPSTMDTIFQQIYLLLAALQA
NTQTSASGDTPANTASGDADTQMSASDWQATQPIEKRTSWPSLGYDFDPKNIKGKDAPPA
LEGSTVTWNDGTLTKSELQIVSTLNAHKDQMPIEYKNLDDKINDPSTPPDLKAALQGLKQ
DPRLFFAIGSQGDGKCGGKVSAQDLWDFSDSHPQVKDLGGKNDEFNPKDIKGSNPPQAAE
GSTVTWNDGQLNQSELEIVSVLDRHKDQVDSLSFDQLDAKINDPSTQPDLKKALKGLQKD
PRLFFAIGSQKDGKCGGKIKAQDLTDFSYYHPQIAEYNDKKAKSYTQNYIASDSPDKTKA
SVMTKSDALREMYRYSDYLPGNLSEDEFAKIVDGDSKTGKCPPQLIAAAQYFRDHPDEWK
EFSGDAGTMSTPDFLQKSTSEMHLTADEQKTLDTINSHQDAFYGDGKELTRDKLDAISKD
NKADPAVKEAATQLASDPLLFGLLNNSITGYKKPHHFFGGGHVVDSGKISQNDFRQFYDH
MSAANKTVNTPATHEASSPDQQKAVADMLMGKDDPPAIKKPKKDVGTFQQGLHEFLKWDS
KILDWMSVGLSALNGIPVIGEIADAAAIALESEAQAAQVVDTAIQGGDMSLALKLAGINM
AGAVVGAVGGPTARIGAKEAAEGAAKGTAKGAAKGAGKTAAERPSAAAFAKGYVAGSTIS
KSTEILKKPVMAGLHYEEYQLDKQKDGEIHQKLDNAGGAPVGKQIIPTGIADNFEGDVRQ
NLRNVRIRRR*
>C5 locus=Scaffold12:56496:58904:+
MSLNMLSTGSNPSQLLGTSSNESSSSELFGSDSSNDGSDLPSTMDTIFQQIYLLLAALQA
NTQTSASGDTPANTASGDADTQMSASDWQATQPIEKRTSWPSLGYDFDPKNIKGKDAPPA
LEGSTVTWNDGTLTKSELQIVSTLNAHKDQMPLEYKNLDDKINDPSTPPDLKAALQGLKQ
DPRLFFAIGSQGDGKCGGKVSAQDLWDFSDSHPQVKDLGGKNDEFNPKDIKGSNPPQAAE
GSTVTWNDGQLNQSELEIVSVLDRHKDQVDSLSFDQLDAKINDPSTQPDLKEALKGLQKD
PRLFFAIGSQKDGKCGGKIKAQDLTDFSYYHPQIAEYNDKKAKSYTQNYIASDSPDKTKA

SVMTKSDALREMYRYSDYLPGNLSEDEFAKIVDGDSKTGKCPPQLIAAAQYFRDHPDEWK
EFSGDAGTMSTPDFLQKSTSEMHLTADEQKTLDTINSHQDAFYGDGKELTRDKLDAISKD
DKADPAVKEAATQLASDPLLFGLLNNSITGYKKPHHFFGGGHVVDSGKISQNDFRQFYDH
MSAANKTVNTPATHEASSPDQQKAVADMLMGKDDPPAIKKPKKDVGTFQQGLHEFLKWDS
KILDWMSVGLSALNGIPVIGEIADAASIALESEAQAAQVVDTAIQGGDMSLALKLAGINM
AGAVVGAVGGPTARIGAKGAAKGVAEVAAKEAAEGAAKGTAKGAAKGAGKTAAERPSAAE
FAKGYVAGSNISKSTEILKTPVMAGLHYEEYQLDKQKDGEIHQKLEHAGGVPVGKQIIPK
GIADNFEGDVRQNLRNVRIRRR*
>C6 locus=Scaffold2:54764:57136:-
MSLNMLSTGSNPSQLLGTSSNESSSSELFGSDSSNDGSDLPSTMDTIFQQIYLLLAALQA
NTQTSASGDTPANTASGDADTQMSASDWQATQPIEKRTSWPSLGYDFDPKNIKGKDAPPA
LEGSTVTWNDGTLTKSELQIVSTLNAHKDQMPIEYKNLDDKINDPSTPPDLKAALQGLKQ
DPRLFFAIGSQGDGKCGGKVSAQDLWDFSDSHPQVKDLGGKNDEFNPKDIKGSNPPQAAE
GSTVTWNDGQLNQSELEIVSVLDRHKDQVDSLSFDQLDAKINDPSTQPDLKKALKGLQKD
PRLFFAIGSQKDGKCGGKIKAQDLTDFSYYHPQIAEYNDKKAKSYTQNYIASDSPDKTKA
SVMTKSDALREMYRYSDYLPGNLSEDEFAKIVDGDSKTGKCPPQLIAAAQYFRDHPDEWK
EFSGDAGTMSTPDFLQKSTSEMHLTADEQKTLDTINSHQDAFYGDGKELTRDKLDAISKD
NKADPAVKEAATQLASDPLLFGLLNNSITGYKKPHHFFGGGHVVDSGKISQNDFRQFYDH
MSAANKTVNTPATHEASSPDQQKAVADMLMGKDDPPAIKKPKKDVGTFQQGLHEFLKWDS
KILDWMSVGLSALNGIPVIGEIADAAAIALESEAQAAQVVDTAIQGGDMSLALKLAGINM
AGAVVGAVGGPTARIGAKEAAEGAAKGTAKGAAKGAGKTAAERPSAAAFAKGYVAGSTIS
KSTEILKKPVMAGLHYEEYQLDKQKDGEIHQKLDNAGGAPVGKQIIPTGIADNFEGDVRQ
NLRNVRIRRR*
>C7 locus=Scaffold11:81986:84394:+
MSLNMLSTGSNPSQLLGTSSNESSSSELFGSDSSNDGSDLPSTMDTIFQQIYLLLAALQA
NTQTSASGDTPANTASGDADTQMSASDWQATQPIEKRTSWPSLGYDFDPKNIKGKDAPPA
LEGSTVTWNDGTLTKSELQIVSTLNAHKDQMPIEYKNLDDKINDPSTPPDLKAALQGLKQ
DPRLFFAIGSQGDGKCGGKVSAQDLWDFSDSHPQVKDLGGKNDEFNPKDIKGSNPPQAAE
GSTVTWNDGQLNQSELEIVSVLDRHKDQVDSLSFDQLDAKINDPSTQPDLKKALKGLQKD
PRLFFAIGSQKDGKCGGKIKAQDLTDFSYYHPQIAEYNDKKAKSYTQNYIASDSPDKTKA
SVMTKSDALREMYRYSDYLPGNLSEDEFAKIVDGDSKTGKCPPQLIAAAQYFRDHPDEWK
EFSGDAGTMSTPDFLQKSTSEMHLTADEQKTLDTINSHQDAFYGDGKELTRDKLDAISKD
NKADPAVKEAATQLASDPLLFGLLNNSITGYKKPHHFFGGGHVVDSGKISQNDFRQFYDH
MSAANKTVNTPATHEASSPDQQKAVADMLMGKDDPPAIKKPKKDVGTFQQGLHEFLKWDS
KILDWMSVGLSALNGIPVIGEIADAAAIALESEAQAAQVVDTAIQGGDMSLALKLAGINM
AGAVVGAVGGPTARIGAKGAAKGVAEVAAKEAAEGAAKGTAKGAAKGAGKTAAERPSAAA
FAKGYVAGSTISKSTEILKKPVMAGLHYEEYQLDKQKDGEIHQKLDNAGGAPVGRQIIPT
GIADNFEGDVRQNLRNVRIRRR*
>P1 locus=Scaffold1:9581:11953:-
MSLNMLSTGSNPSQLLGTSSNESSSSELFGSDSSNDGSDLPSTMDTIFQQIYLLLAALQA
NTQTSASGDTPANTASGDADTQMSASDWQATQPIEKRTSWPSLGYDFDPKNIKGKDAPPA
LEGSTVTWNDGTLTKSELQIVSTLNAHKDQMPIEYKNLDDKINDPSTPPDLKAALQGLKQ
DPRLFFAIGSQGDGKCGGKVSAQDLWDFSDSHPQVKDLGGKNDEFNPKDIKGSNPPQAAE
GSTVTWNDGQLNQSELEIVSVLDRHKDQVDSLSFDQLDAKINDPSTQPDLKKALKGLQKD

PRLFFAIGSQKDGKCGGKIKAQDLTDFSYYHPQIAEYNDKKAKSYTQNYIASDSPDKTKA
SVMTKSDALREMYRYSDYLPGNLSEDEFAKIVDGDSKTGKCPPQLIAAAQYFRDHPDEWK
EFSGDAGTMSTPDFLQKSTSEMHLTADEQKTLDTINSHQDAFYGDGKELTRDKLDAISKD
NKADPAVKEAATQLASDPLLFGLLNNSITGYKKPHHFFGGGHVVDSGKISQNDFRQFYDH
MSAANKTVNTPATHEASSPDQQKAVADMLMGKDDPPAIKKPKKDVGTFQQGLHEFLKWDS
KILDWMSVGLSALNGIPVIGEIADAAAIALESEAQAAQVVDTAIQGGDMSLALKLAGINM
AGAVVGAVGGPTARIGAKEAAEGAAKGTAKGAAKGAGKTAAERPSAAAFAKGYVAGSTIS
KSTEILKKPVMAGLHYEEYQLDKQKDGEIHQKLDNAGGAPVGKQIIPTGIADNFEGDVRQ
NLRNVRIRRR*
>P6d locus=Scaffold4:209838:212246:-
MSLNMLSTGSNPSQLLGTSSNESSSSELFGSDSSNDGSDLPSTMDTIFQQIYLLLAALQA
NTQTSASGDTPANTASGDADTQMSASDWQATQPIEKRTSWPSLGYDFDPKNIKGKDAPPA
LEGSTVTWNDGTLTKSELQIVSTLNAHKDQMPIEYKNLDDKINDPSTPPDLKAALQGLKQ
DPRLFFAIGSQGDGKCGGKVSAQDLWDFSDSHPQVKDLGGKNDEFNPKDIKGSNPPQAAE
GSTVTWNDGQLNQSELEIVSVLDRHKDQVDSLSFDQLDAKINDPSTQPDLKKALKGLQKD
PRLFFAIGSQKDGKCGGKIKAQDLTDFSYYHPQIAEYNDKKAKSYTQNYIASDSPDKTKA
SVMTKSDALREMYRYSDYLPGNLSEDEFAKIVDGDSKTGKCPPQLIAAAQYFRDHPDEWK
EFSGDAGTMSTPDFLQKSTSEMHLTADEQKTLDTINSHQDAFYGDGKELTRDKLDAISKD
NKADPAVKEAATQLASDPLLFGLLNNSITGYKKPHHFFGGGHVVDSGKISQNDFRQFYDH
MSAANKTVNTPATHEASSPDQQKAVADMLMGKDDPPAIKKPKKDVGTFQQGLHEFLKWDS
KILDWMSVGLSALNGIPVIGEIADAAAIALESEAQAAQVVDTAIQGGDMSLALKLAGINM
AGAVVGAVGGPTARIGAKGAAKGVAEVAAKEAAEGAAKGTAKGSAKGAGKTAAERPSAAA
FAKGYVAGSTISKSTEILKKPVMAGLHYEEYQLDKQKDGEIHQKLDNAGGAPVGKQIIPT
GIADNFEGDVRQNLRNVRIRRR*
>P3b locus=Scaffold3:212408:214780:-
MSLNMLSTGSNPSQLLGTSSNESSSSELFGSDSSNDGSDLPSTMDTIFQQIYLLLAALQA
NTQTSASGDTPANTASGDADTQMSASDWQATQPIEKRTSWPSLGYDFDPKNIKGKDAPPA
LEGSTVTWNDGTLTKSELQIVSTLNAHKDQMPIEYKNLDDKINDPSTPPDLKAALQGLKQ
DPRLFFAIGSQGDGKCGGKVSAQDLWDFSDSHPQVKDLGGKNDEFNPKDIKGSNPPQAAE
GSTVTWNDGQLNQSELEIVSVLDRHKDQVDSLSFDQLDAKINDPSTQPDLKKALKGLQKD
PRLFFAIGSQKDGKCGGKIKAQDLTDFSYYHPQIAEYNDKKAKSYTQNYIASDSPDKTKA
SVMTKSDALREMYRYSDYLPGNLSEDEFAKIVDGDSKTGKCPPQLIAAAQYFRDHPDEWK
EFSGDAGTMSTPDFLQKSTSEMHLTADEQKTLDTINSHQDAFYGDGKELTRDKLDAISKD
NKADPAVKEAATQLASDPLLFGLLNNSITGYKKPHHFFGGGHVVDSGKISQNDFRQFYDH
MSAANKTVNTPATHEASSPDQQKAVADMLMGKDDPPAIKKPKKDVGTFQQGLHEFLKWDS
KILDWMSVGLSALNGIPVIGEIADAAAIALESEAQAAQVVDTAIQGGDMSLALKLAGINM
AGAVVGAVGGPTARIGAKEAAEGAAKGTAKGAAKGAGKTAAERPSAAAFAKGYVAGSTIS
KSTEILKKPVMAGLHYEEYQLDKQKDGEIHQKLDNAGGAPVGKQIIPTGIADNFEGDVRQ
NLRNVRIRRR*
>P3c locus=Scaffold3:192692:195100:+
MSLNMLSTGSNPSQLLGTSSNESSSSDLFGSDSSRDGFDLPSTMDTLFQQIYLVLAALQA
NTQTSASGDTPANTASGDADTQMSASDWQATQPIEKRTSWPSLGYDFDPKNIKGKDAPPA
LEGSTVTWNDGTLTKSELQIVSTLNAHKDQMPLEYKNLDDKINDPSTPPDLKAALQGLKQ
DPRLFFAIGSQGDGKCGGKVSAQDLWDFSDSHPQVKDLGGKNDEFNPKDIKGSNPPQAAE

GSTVTWNDGQLNQSELEIVSVLDRHKDQVDSLSFDQLDAKINDPSTQPDLKKALKGLQKD
PRLFFAIGSQKDGKCGGKIKAQDLTDFSYYHPQIAEYNDKKAKSYTQNYIASDSPDKTKA
SVMTKSDALREMYRYSDYLPGNLSEDEFAKIVDGDSKTGKCPPQLIAAAQYFREHPDEWK
EFSGDAGTMSTPDFLQKSTSEMHLTADEQKTLDTINSHQDAFYGDGKELTRDKLDAISKD
DKADPAVKEAATQLASDPLLFGLLNNSITGYKKPHHFFGGGHVVDSGKISQNDFRQFYDH
MSAANKTVNTPATHEASSPDQQKAVADMLMGKDDPPAIKKPKKDVGTFQQGLHEFLKWDS
KILDWMSVGLSALNGIPVIGEIADAAAIALESEAQAAQVVDTAIQGGDMSLALKLAGINM
AGAVVGAVGGPTARIGAKGAAKGVAEVAAKEAAEGAAKGTANGAAKGAGKTAAERPSAAE
FAKGYVAGSTISKSTEILKTPVMAGLHYEEYQLDKQKDGEIHQKLDNAGGAPVGKQIIPK
GIADNFEGDVRQNLRNVRIRRR*
>P4 locus=Scaffold5:196334:198706:+
MSLNMLSTGSNPSQLLGTSSNESSSSELFGSDSSNDGSDLPSTMDTIFQQIYLLLAALQA
NTQTSASGDTPANTASGDADTQMSASDWQATQPIEKRTSWPSLGYDFDPKNIKGKDAPPA
LEGSTVTWNDGTLTKSELQIVSTLNAHKDQMPIEYKNLDDKINDPSTPPDLKAALQGLKQ
DPRLFFAIGSQGDGKCGGKVSAQDLWDFSDSHPQVKDLGGKNDEFNPKDIKGSNPPQAAE
GSTVTWNDGQLNQSELEIVSVLDRHKDQVDSLSFDQLDAKINDPSTQPDLKKALKGLQKD
PRLFFAIGSQKDGKCGGKIKAQDLTDFSYYHPQIAEYNDKKAKSYTQNYIASDSPDKTKA
SVMTKSDALREMYRYSDYLPGNLSEDEFAKIVDGDSKTGKCPPQLIAAAQYFRDHPDEWK
EFSGDAGTMSTPDFLQKSTSEMHLTADEQKTLDTINSHQDAFYGDGKELTRDKLDAISKD
NKADPAVKEAATQLASDPLLFGLLNNSITGYKKPHHFFGGGHVVDSGKISQNDFRQFYDH
MSAANKTVNTPATHEASSPDQQKAVADMLMGKDDPPAIKKPKKDVGTFQQGLHEFLKWDS
KILDWMSVGLSALNGIPVIGEIADAAAIALESEAQAAQVVDTAIQGGDMSLALKLAGINM
AGAVVGAVGGPTARIGAKEAAEGAAKGTAKGAAKGAGKTAAERPSAAAFAKGYVAGSTIS
KSTEILKKPVMAGLHYEEYQLDKQKDGEIHQKLDNAGGAPVGKQIIPTGIADNFEGDVRQ
NLRNVRIRRR*
>P5 locus=Scaffold7:99413:101785:+
MSLNMLSTGSNPSQLLGTSSNESSSSELFGSDSSNDGSDLPSTMDTIFQQIYLLLAALQA
NTQTSASGDTPANTASGDADTQMSASDWQATQPIEKRTSWPSLGYDFDPKNIKGKDAPPA
LEGSTVTWNDGTLTKSELQIVSTLNAHKDQMPIEYKNLDDKINDPSTPPDLKAALQGLKQ
DPRLFFAIGSQGDGKCGGKVSAQDLWDFSDSHPQVKDLGGKNDEFNPKDIKGSNPPQAAE
GSTVTWNDGQLNQSELEIVSVLDRHKDQVDSLSFDQLDAKINDPSTQPDLKKALKGLQKD
PRLFFAIGSQKDGKCGGKIKAQDLTDFSYYHPQIAEYNDKKAKSYTQNYIASDSPDKTKA
SVMTKSDALREMYRYSDYLPGNLSEDEFAKIVDGDSKTGKCPPQLIAAAQYFRDHPDEWK
EFSGDAGTMSTPDFLQKSTSEMHLTADEQKTLDTINSHQDAFYGDGKELTRDKLDAISKD
NKADPAVKEAATQLASDPLLFGLLNNSITGYKKPHHFFGGGHVVDSGKISQNDFRQFYDH
MSAANKTVNTPATHEASSPDQQKAVADMLMGKDDPPAIKKPKKDVGTFQQGLHEFLKWDS
KILDWMSVGLSALNGIPVIGEIADAAAIALESEAQAAQVVDTAIQGGDMSLALKLAGINM
AGAVVGAVGGPTARIGAKEAAEGAAKGTAKGAAKGAGKTAAERPSAAAFAKGYVAGSTIS
KSTEILKKPVMAGLHYEEYQLDKQKDGEIHQKLDNAGGAPVGKQIIPTGIADNFEGDVRQ
NLRNVRIRRR*
>P6 locus=Scaffold1:1333966:1336374:+
MSLNMLSTGSNPSQLLGTSSNESSSSELFGSDSSNDGSDLPSTMDTIFQQIYLLLAALQA
NTQTSASGDTPANTASGDADTQMSASDWQATQPIEKRTSWPSLGYDFDPKNIKGKDAPPA
LEGSTVTWNDGTLTKSELQIVSTLNAHKDQMPIEYKNLDDKINDPSTPPDLKAALQGLKQ

DPRLFFAIGSQGDGKCGGKVSAQDLWDFSDSHPQVKDLGGKNDEFNPKDIKGSNPPQAAE
GSTVTWNDGQLNQSELEIVSVLDRHKDQVDSLSFDQLDAKINDPSTQPDLKKALKGLQKD
PRLFFAIGSQKDGKCGGKIKAQDLTDFSYYHPQIAEYNDKKAKSYTQNYIASDSPDKTKA
SVMTKSDALREMYRYSDYLPGNLSEDEFAKIVDGDSKTGKCPPQLIAAAQYFRDHPDEWK
EFSGDAGTMSTPDFLQKSTSEMHLTADEQKTLDTINSHQDAFYGDGKELTRDKLDAISKD
NKADPAVKEAATQLASDPLLFGLLNNSITGYKKPHHFFGGGHVVDSGKISQNDFRQFYDH
MSAANKTVNTPATHEASSPDQQKAVADMLMGKDDPPAIKKPKKDVGTFQQGLHEFLKWDS
KILDWMSVGLSALNGIPVIGEIADAAAIALESEAQAAQVVDTAIQGGDMSLALKLAGINM
AGAVVGAVGGPTARIGAKGAAKGVAEVAAKEAAEGAAKGTAKGSAKGAGKTAAERPSAAA
FAKGYVAGSTISKSTEILKKPVMAGLHYEEYQLDKQKDGEIHQKLDNAGGAPVGKQIIPT
GIADNFEGDVRQNLRNVRIRRR*
>P7 locus=Scaffold20:32063:34435:+
MSLNMLSTGSNPSQLLGTSSNESSSSELFGSDSSNDGSDLPSTMDTIFQQIYLLLAALQA
NTQTSASGDTPANTASGDADTQMSASDWQATQPIEKRTSWPSLGYDFDPKNIKGKDAPPA
LEGSTVTWNDGTLTKSELQIVSTLNAHKDQMPIEYKNLDDKINDPSTPPDLKAALQGLKQ
DPRLFFAIGSQGDGKCGGKVSAQDLWDFSDSHPQVKDLGGKNDEFNPKDIKGSNPPQAAE
GSTVTWNDGQLNQSELEIVSVLDRHKDQVDSLSFDQLDAKINDPSTQPDLKKALKGLQKD
PRLFFAIGSQKDGKCGGKIKAQDLTDFSYYHPQIAEYNDKKAKSYTQNYIASDSPDKTKA
SVMTKSDALREMYRYSDYLPGNLSEDEFAKIVDGDSKTGKCPPQLIAAAQYFRDHPDEWK
EFSGDAGTMSTPDFLQKSTSEMHLTADEQKTLDTINSHQDAFYGDGKELTRDKLDAISKD
NKADPAVKEAATQLASDPLLFGLLNNSITGYKKPHHFFGGGHVVDSGKISQNDFRQFYDH
MSAANKTVNTPATHEASSPDQQKAVADMLMGKDDPPAIKKPKKDVGTFQQGLHEFLKWDS
KILDWMSVGLSALNGIPVIGEIADAAAIALESEAQAAQVVDTAIQGGDMSLALKLAGINM
AGAVVGAVGGPTARIGAKEAAEGAAKGTAKGAAKGAGKTAAERPSAAAFAKGYVAGSTIS
KSTEILKKPVMAGLHYEEYQLDKQKDGEIHQKLDNAGGAPVGKQIIPTGIADNFEGDVRQ
NLRNVRIRRR*
>P8 locus=Scaffold7:61947:64319:+
MSLNMLSTGSNPSQLLGTSSNESSSSELFGSDSSNDGSDLPSTMDTIFQQIYLLLAALQA
NTQTSASGDTPANTASGDADTQMSASDWQATQPIEKRTSWPSLGYDFDPKNIKGKDAPPA
LEGSTVTWNDGTLTKSELQIVSTLNAHKDQMPIEYKNLDDKINDPSTPPDLKAALQGLKQ
DPRLFFAIGSQGDGKCGGKVSAQDLWDFSDSHPQVKDLGGKNDEFNPKDIKGSNPPQAAE
GSTVTWNDGQLNQSELEIVSVLDRHKDQVDSLSFDQLDAKINDPSTQPDLKKALKGLQKD
PRLFFAIGSQKDGKCGGKIKAQDLTDFSYYHPQIAEYNDKKAKSYTQNYIASDSPDKTKA
SVMTKSDALREMYRYSDYLPGNLSEDEFAKIVDGDSKTGKCPPQLIAAAQYFRDHPDEWK
EFSGDAGTMSTPDFLQKSTSEMHLTADEQKTLDTINSHQDAFYGDGKELTRDKLDAISKD
NKADPAVKEAATQLASDPLLFGLLNNSITGYKKPHHFFGGGHVVDSGKISQNDFRQFYDH
MSAANKTVNTPATHEASSPDQQKAVADMLMGKDDPPAIKKPKKDVGTFQQGLHEFLKWDS
KILDWMSVGLSALNGIPVIGEIADAAAIALESEAQAAQVVDTAIQGGDMSLALKLAGINM
AGAVVGAVGGPTARIGAKEAAEGAAKGTAKGAAKGAGKTAAERPSAAAFAKGYVAGSTIS
KSTEILKKPVMAGLHYEEYQLDKQKDGEIHQKLDNAGGAPVGKQIIPTGIADNFEGDVRQ
NLRNVRIRRR*
>P9a locus=Scaffold2:307128:309536:+
MSLNMLSTGSNPSQLLGTSSNESSSSDLFGSDSSRDGFDLPSTMDTLFQQIYLVLAALQA
NTQTSASGDTPANTASGDADTQMSASDWQATQPIEKRTSWPSLGYDFDPKNIKGKDAPPA

LEGSTVTWNDGTLTKSELQIVSTLNAHKDQMPLEYKNLDDKINDPSTPPDLKAALQGLKQ
DPRLFFAIGSQGDGKCGGKVSAQDLWDFSDSHPQVKDLGGKNDEFNPKDIKGSNPPQAAE
GSTVTWNDGQLNQSELEIVSVLDRHKDQVDSLSFDQLDAKINDPSTQPDLKKALKGLQKD
PRLFFAIGSQKDGKCGGKIKAQDLTDFSYYHPQIAEYNDKKAKSYTQNYIASDSPDKTKA
SVMTKSDALREMYRYSDYLPGNLSEDEFAKIVDGDSKTGKCPPQLIAAAQYFREHPDEWK
EFSGDAGTMSTPDFLQKSTSEMHLTADEQKTLDTINSHQDAFYGDGKELTRDKLDAISKD
DKADPAVKEAATQLASDPLLFGLLNNSITGYKKPHHFFGGGHVVDSGKISQNDFRQFYDH
MSAANKTVNTPATHEASSPDQQKAVADMLMGKDDPPAIKKPKKDVGTFQQGLHEFLKWDS
KILDWMSVGLSALNGIPVIGEIADAAAIALESEAQAAQVVDTAIQGGDMSLALKLAGINM
AGAVVGAVGGPTARIGAKGAAKGVAEVAAKEAAEGAAKGTANGAAKGAGKTAAERPSAAE
FAKGYVAGSTISKSTEILKTPVMAGLHYEEYQLDKQKDGEIHQKLDNAGGAPVGKQIIPK
GIADNFEGDVRQNLRNVRIRRR*
>P9b locus=Scaffold1:99125:101533:-
MSLNMLSTGSNPSQLLGTSSNESSSSDLFGSDSSRDGFDLPSTMDTLFQQIYLVLAALQA
NTQTSASGDTPANTASGDADTQMSASDWQATQPIEKRTSWPSLGYDFDPKNIKGKDAPPA
LEGSTVTWNDGTLTKSELQIVSTLNAHKDQMPLEYKNLDDKINDPSTPPDLKAALQGLKQ
DPRLFFAIGSQGDGKCGGKVSAQDLWDFSDSHPQVKDLGGKNDEFNPKDIKGSNPPQAAE
GSTVTWNDGQLNQSELEIVSVLDRHKDQVDSLSFDQLDAKINDPSTQPDLKKALKGLQKD
PRLFFAIGSQKDGKCGGKIKAQDLTDFSYYHPQIAEYNDKKAKSYTQNYIASDSPDKTKA
SVMTKSDALREMYRYSDYLPGNLSEDEFAKIVDGDSKTGKCPPQLIAAAQYFREHPDEWK
EFSGDAGTMSTPDFLQKSTSEMHLTADEQKTLDTINSHQDAFYGDGKELTRDKLDAISKD
DKADPAVKEAATQLASDPLLFGLLNNSITGYKKPHHFFGGGHVVDSGKISQNDFRQFYDH
MSAANKTVNTPATHEASSPDQQKAVADMLMGKDDPPAIKKPKKDVGTFQQGLHEFLKWDS
KILDWMSVGLSALNGIPVIGEIADAAAIALESEAQAAQVVDTAIQGGDMSLALKLAGINM
AGAVVGAVGGPTARIGAKGAAKGVAEVAAKEAAEGAAKGTANGAAKGAGKTAAERPSAAE
FAKGYVAGSTISKSTEILKTPVMAGLHYEEYQLDKQKDGEIHQKLDNAGGAPVGKQIIPK
GIADNFEGDVRQNLRNVRIRRR*
>P9c locus=Scaffold3:56720:59128:-
MSLNMLSTGSNPSQLLGTSSNESSSSDLFGSDSSRDGFDLPSTMDTLFQQIYLVLAALQA
NTQTSASGDTPANTASGDADTQMSASDWQATQPIEKRTSWPSLGYDFDPKNIKGKDAPPA
LEGSTVTWNDGTLTKSELQIVSTLNAHKDQMPLEYKNLDDKINDPSTPPDLKAALQGLKQ
DPRLFFAIGSQGDGKCGGKVSAQDLWDFSDSHPQVKDLGGKNDEFNPKDIKGSNPPQAAE
GSTVTWNDGQLNQSELEIVSVLDRHKDQVDSLSFDQLDAKINDPSTQPDLKKALKGLQKD
PRLFFAIGSQKDGKCGGKIKAQDLTDFSYYHPQIAEYNDKKAKSYTQNYIASDSPDKTKA
SVMTKSDALREMYRYSDYLPGNLSEDEFAKIVDGDSKTGKCPPQLIAAAQYFREHPDEWK
EFSGDAGTMSTPDFLQKSTSEMHLTADEQKTLDTINSHQDAFYGDGKELTRDKLDAISKD
DKADPAVKEAATQLASDPLLFGLLNNSITGYKKPHHFFGGGHVVDSGKISQNDFRQFYDH
MSAANKTVNTPATHEASSPDQQKAVADMLMGKDDPPAIKKPKKDVGTFQQGLHEFLKWDS
KILDWMSVGLSALNGIPVIGEIADAAAIALESEAQAAQVVDTAIQGGDMSLALKLAGINM
AGAVVGAVGGPTARIGAKGAAKGVAEVAAKEAAEGAAKGTANGAAKGAGKTAAERPSAAE
FAKGYVAGSTISKSTEILKTPVMAGLHYEEYQLDKQKDGEIHQKLDNAGGAPVGKQIIPK
GIADNFEGDVRQNLRNVRIRRR*
>P9d locus=Scaffold2:71657:74065:-
MSLNMLSTGSNPSQLLGTSSNESSSSDLFGSDSSRDGFDLPSTMDTLFQQIYLVLAALQA

NTQTGASGDTPANTASGDADTQMSASDWQATQPIEKRTSWPSLDYDFDPKNIKGKDAPPA
LEGSTVTWNDGTLTKSELQIVSTLNAHKDQMPLEYKNLDDKINDPSTPPDLKAALQGLKQ
DPRLFFAIGSQGDGKCGGKVSAQDLWDFSDSHPQVKDLGGKNDEFNPKDIKGSNPPQAAE
GSTVTWNDGQLNQSELEIVSVLDRHKDQVDSLSFDQLDAKINDPSTQPDLKEALKGLQKD
PRLFFAIGSQKDGKCGGKIKAQDLTDFSYYHPQIAEYNDKKAKSYTQNYIASDSPDKTKA
SVMTKSDALREMYRYSDYLPGNLSEDEFAKIVDGDSKTGKCPPQLIAAAQYFRDHPDEWK
EFSGDAGTMSTPDFLQKSTSEMHLTADEQKTLDTINSHQDAFYGDGKELTRDKLDAISKD
DKADPAVKEAATQLASDPLLFGLLNNSITGYKKPHHFFGGGHVVDSGKISQNDFRQFYDH
MSAANKTVNTPATHEASSPDQQKAVADMLMGKDDPPAIKKPKKDVGTFQQGLHEFLKWDS
KILDWMSVGLSALNGIPVIGEIADAASIALESEAQAAQVVDTAIQGGDMSLALKLAGINM
AGAVVGAVGGPTARIGAKGAAKGVAEVAAKEAAEGAAKGTAKGAAKGAGKTAAERPSAAE
FAKGYVAGSNISKSTEILKTPVMAGLHYEEYQLDKQKDGEIHQKLEHAGGVPVGKQIIPK
GIADNFEGDVRQNLRNVRIRRR*
>P10 locus=Scaffold6:193445:195817:+
MSLNMLSTGSNPSQLLGTSSNESSSSELFGSDSSNDGSDLPSTMDTIFQQIYLLLAALQA
NTQTSASGDTPANTASGDADTQMSASDWQATQPIEKRTSWPSLGYDFDPKNIKGKDAPPA
LEGSTVTWNDGTLTKSELQIVSTLNAHKDQMPIEYKNLDDKINDPSTPPDLKAALQGLKQ
DPRLFFAIGSQGDGKCGGKVSAQDLWDFSDSHPQVKDLGGKNDEFNPKDIKGSNPPQAAE
GSTVTWNDGQLNQSELEIVSVLDRHKDQVDSLSFDQLDAKINDPSTQPDLKKALKGLQKD
PRLFFAIGSQKDGKCGGKIKAQDLTDFSYYHPQIAEYNDKKAKSYTQNYIASDSPDKTKA
SVMTKSDALREMYRYSDYLPGNLSEDEFAKIVDGDSKTGKCPPQLIAAAQYFRDHPDEWK
EFSGDAGTMSTPDFLQKSTSEMHLTADEQKTLDTINSHQDAFYGDGKELTRDKLDAISKD
NKADPAVKEAATQLASDPLLFGLLNNSITGYKKPHHFFGGGHVVDSGKISQNDFRQFYDH
MSAANKTVNTPATHEASSPDQQKAVADMLMGKDDPPAIKKPKKDVGTFQQGLHEFLKWDS
KILDWMSVGLSALNGIPVIGEIADAAAIALESEAQAAQVVDTAIQGGDMSLALKLAGINM
AGAVVGAVGGPTARIGAKEAAEGAAKGTAKGAAKGAGKTAAERPSAAAFAKGYVAGSTIS
KSTEILKKPVMAGLHYEEYQLDKQKDGEIHQKLDNAGGAPVGKQIIPTGIADNFEGDVRQ
NLRNVRIRRR*
>GIV locus=Scaffold4:53942:56314:-
MSLNMLSTGSNPSQLLGTSSNESSSSELFGSDSSNDGSDLPSTMDTIFQQIYLLLAALQA
NTQTSASGDTPANTASGDADTQMSASDWQATQPIEKRTSWPSLGYDFDPKNIKGKDAPPA
LEGSTVTWNDGTLTKSELQIVSTLNAHKDQMPIEYKNLDDKINDPSTPPDLKAALQGLKQ
DPRLFFAIGSQGDGKCGGKVSAQDLWDFSDSHPQVKDLGGKNDEFNPKDIKGSNPPQAAE
GSTVTWNDGQLNQSELEIVSVLDRHKDQVDSLSFDQLDAKINDPSTQPDLKKALKGLQKD
PRLFFAIGSQKDGKCGGKIKAQDLTDFSYYHPQIAEYNDKKAKSYTQNYIASDSPDKTKA
SVMTKSDALREMYRYSDYLPGNLSEDEFAKIVDGDSKTGKCPPQLIAAAQYFRDHPDEWK
EFSGDAGTMSTPDFLQKSTSEMHLTADEQKTLDTINSHQDAFYGDGKELTRDKLDAISKD
NKADPAVKEAATQLASDPLLFGLLNNSITGYKKPHHFFGGGHVVDSGKISQNDFRQFYDH
MSAANKTVNTPATHEASSPDQQKAVADMLMGKDDPPAIKKPKKDVGTFQQGLHEFLKWDS
KILDWMSVGLSALNGIPVIGEIADAAAIALESEAQAAQVVDTAIQGGDMSLALKLAGINM
AGAVVGAVGGPTARIGAKEAAEGAAKGTAKGAAKGAGKTAAERPSAAAFAKGYVAGSTIS
KSTEILKKPVMAGLHYEEYQLDKQKDGEIHQKLDNAGGAPVGKQIIPTGIADNFEGDVRQ
NLRNVRIRRR*
>GV locus=Scaffold15:13748:16156:-

MSLNMLSTGSNPSQLLGTSSNESSSSELFGSDSSNDGSDLPSTMDTIFQQIYLLLAALQA
NTQTSASGDTPANTASGDADTQMSASDWQATQPIEKRTSWPSLGYDFDPKNIKGKDAPPA
LEGSTVTWNDGTLTKSELQIVSTLNAHKDQMPLEYKNLDDKINDPSTPPDLKAALQGLKQ
DPRLFFAIGSQGDGKCGGKVSAQDLWDFSDSHPQVKDLGGKNDEFNPKDIKGSNPPQAAE
GSTVTWNDGQLNQSELEIVSVLDRHKDQVDSLSFDQLDAKINDPSTQPDLKEALKGLQKD
PRLFFAIGSQKDGKCGGKIKAQDLTDFSYYHPQIAEYNDKKAKSYTQNYIASDSPDKTKA
SVMTKSDALREMYRYSDYLPGNLSEDEFAKIVDGDSKTGKCPPQLIAAAQYFRDHPDEWK
EFSGDAGTMSTPDFLQKSTSEMHLTADEQKTLDTINSHQDAFYGDGKELTRDKLDAISKD
DKADPAVKEAATQLASDPLLFGLLNNSITGYKKPHHFFGGGHVVDSGKISQNDFRQFYDH
MSAANKTVNTPATHEASSPDQQKAVADMLMGKDDPPAIKKPKKDVGTFQQGLHEFLKWDS
KILDWMSVGLSALNGIPVIGEIADAASIALESEAQAAQVVDTAIQGGDMSLALKLAGINM
AGAVVGAVGGPTARIGAKGAAKGVAEVAAKEAAEGAAKGTAKGAAKGAGKTAAERPSAAE
FAKGYVAGSNISKSTEILKTPVMAGLHYEEYQLDKQKDGEIHQKLEHAGGVPVGKQIIPK
GIADNFEGDVRQNLRNVRIRRR*

Tree file for PXO_03417 (Supports Supplemental Figure S2A)

((P9d:0.00252606,(P9c:0.00000001,(P9b:0.00000001,(P3c:0.00000001,P9a:0.00000001)163:0.00000001)202:0.00000001)962:0.01022609)500:0.00642864,GV:0.00000001,(C5:0.00000010,((P6:0.00000001,(PXO99A:0.00000001,P6d:0.00000001)239:0.00000001)640:0.00124408,((C3:0.00000001,(C4:0.00000001,(C6:0.00000001,(P1:0.00000001,(P3b:0.00000001,(P4:0.00000001,(P5:0.00000001,(P7:0.00000001,(P8:0.00000001,(P10:0.00000001,GIV:0.00000001)18:0.00000001)4:0.00000001)1:0.00000001)3:0.00000001)1:0.00000001)1:0.00000001)2:0.00000001)6:0.00000001)16:0.00000001)60:0.00000001,(C2:0.00000001,(C1:0.00000001,C7:0.00000001)650:0.00124279)9:0.00000001)242:0.00000007)995:0.01407116)68:0.00000008);

cds.prot.fasta for PXO_01644 (Supports Supplemental Figure S3A)

>PXO99A [gene=PXO_01644] [protein=TonB-dependent receptor] [protein_id=YP_001914282.1] [location=3247479..3250106]
MAAGQEQQAPASTPSATTELDTITVTGYRASLEKSQSVKRAANSIVDAISAEDIGKFPDT
NAAESLAHVPGISVDRQFGEGEKVSINGTDPALNRVLLNGQTIASGDWGGNPSDTSGRTF
NYTLLSPEIIGLMEVYKTPEARIDEGSIGGTVIVHTRKPLDLPKNTIRGSVGYNYNNRSK
EGNPRGSALWSWKNDDETFGALISATHDKQDLARAGIEYFGYTTGDKIPPTATITGDGSN
VATARVPAGISSAFFQQTRERNGLQGALQWKPDENNEFNLTGIYIKGKYNNYSQARYVCP
ACNDDLKKVTRANVENGVVTSATVSDNTQGGVNDQPYAQMDTNYRESTVTTKSLNLRHDW
SGEKWVFTTQIGDTEATGGKNPEYLMKYLMQDGGYNYAFDGRNTAVNYDNGGAANWALPG
NPAGLAPGAETIPGTSPSVSPMQAGGIYYQKSKDQEKYFQWDASRDLALGPFNKLQFGYK
YINHDNGVDARGNRINTTDPVSLTQFNPGTTQSSLYDGLGASGDLTTWPTANLGAVRRYL
LAQPQGPYNTDFGGSFDVKEITQNVYTQLNFESGQWRGNVGVRLLDTTDKSEYWQSPDNG
QSYSRVAETHEYRKALPSFNVAYDVTDDAVLRFSVAKVMARPRYADLAGSFTVNSGNGNL
TASGGNPDLKPYESTNYDLAAEWYFAPSSMLSGEVFYRDISSYIVSTTVGEQRPATNLNP
AGLYQVTTPTNASDAKVKGASINYQQTFGLGFGLQANYTYAKADASTGLNLPYLSRDTYN
VIPYWEHGDWTVRVNYSYRSKYFTQLGRLASEDFADSYKQLDLTASYQITDYMGLTFGAT
NLLDSTYKLYSGTRDTPTAFYKNGRGYQAQLNFKF*

>C1 locus=Scaffold4:300688:301917:-
LLIGGVGVGGAMALPASEQQPPVPAPSQPQSAPASVQEPAPASPWVSTLDGIKVVGYQAS
LGKALNVKRNADAIIDAISAEDIGKFPDTNVAESLSRLSGITVDRQFGEGEKVSILGTDP
ALNRVLLNGQAIASTSWGGDPNDPDSSSFNYSTLAPEVVGLMEVYKTPEARIDEGSIGGT
VIVNTRKPLDLERNTFTGTVTYGYNDRPDEGKPNASVLYSWKNQDETLGVLTSVMHSQRL
LRRDGVEIFGYDNVAGAAFPPAVVGNNTGVFPTSINTALFQQTRKRDGCSAALQWKPDSE
FELNLTGPYVKESFDNYNQSRYGYWGSNPGDAQALGFENGVATSGTFGDQSNTYLDGYLR
NSEVTTGSIHLRTDWHGDGWNASSQVGYTSSQGGAERIYGIQIPQSGWL*
>C2 locus=Scaffold7:297693:298787:-
VSTLDGIKVVGYQASLGKALNVKRNADAIIDAISAEDIGKFPDTNVAESLSRLSGITVDR
QFGEGEKVSILGTDPALNRVLLNGQAIASTSWGGDPNDPDSSSFNYSTLAPEVVGLMEVY
KTPEARIDEGSIGGTVIVNTRKPLDLERNTFTGTVTYGYNDRPDEGKPNASVLYSWKNQD
ETLGVLTSVMHSQRLLRRDGVEIFGYDNVAGAAFPPAVVGNNTGVFPTSINTALFQQTRK
RDGCSAALQWKPDSEFELNLTGPYVKESFDNYNQSRYGYWGSNPGDAQALGFENGVATSG
TFGDQSNTYLDGYLRNSEVTTGSIHLRTDWHGDGWNASSQVGYTSSQGGAERIYGIQIPQ
SGWL*
>C3 locus=Scaffold5:152731:153825:-
VSTLDGIKVVGYQASLGKALNVKRNADAIIDAISAEDIGKFPDTNVAESLSRLSGITVDR
QFGEGEKVSILGTDPALNRVLLNGQAIASTSWGGDPNDPDSSSFNYSTLAPEVVGLMEVY
KTPEARIDEGSIGGTVIVNTRKPLDLERNTFTGTVTYGYNDRPDEGKPNASVLYSWKNQD
ETLGVLTSVMHSQRLLRRDGVEIFGYDNVAGAAFPPAVVGNNTGVFPTSINTALFQQTRK
RDGCSAALQWKPDSEFELNLTGPYVKESFDNYNQSRYGYWGSNPGDAQALGFENGVATSG
TFGDQSNTYLDGYLRNSEVTTGSIHLRTDWHGDGWNASSQVGYTSSQGGAERIYGIQIPQ
SGWL*
>C4 locus=Scaffold5:117336:118430:-
VSTLDGIKVVGYQASLGKALNVKRNADAIIDAISAEDIGKFPDTNVAESLSRLSGITVDR
QFGEGEKVSILGTDPALNRVLLNGQAIASTSWGGDPNDPDSSSFNYSTLAPEVVGLMEVY
KTPEARIDEGSIGGTVIVNTRKPLDLERNTFTGTVTYGYNDRPDEGKPNASVLYSWKNQD
ETLGVLTSVMHSQRLLRRDGVEIFGYDNVAGAAFPPAVVGNNTGVFPTSINTALFQQTRK
RDGCSAALQWKPDSEFELNLTGPYVKESFDNYNQSRYGYWGSNPGDAQALGFENGVATSG
TFGDQSNTYLDGYLRNSEVTTGSIHLRTDWHGDGWNASSQVGYTSSQGGAERIYGIQIPQ
SGWL*
>C5 locus=Scaffold5:338029:338361:+
LSRPSLPYNSRNAYNISPYYEQGKWSARVNLGWRSEYLTQIGRLNGQQMTDAFTQVDASF
GYQATERLRVALEATNLLDETYFSYIGNKNQPYYIYKNGRSFMLSLNFKL*
>C6 locus=Scaffold21:26708:27040:+
LSRPSLPYNSRNAYNISPYYEQGKWSARVNLGWRSEYLTQIGRLNGQQMTDAFTQVDASF
GYQATERLRVALEATNLLDETYFSYIGNKNQPYYIYKNGRSFMLSLNFKL*
>C7 locus=Scaffold8:7991:9085:-
VSTLDGIKVVGYQASLGKALNVKRNADAIIDAISAEDIGKFPDTNVAESLSRLSGITVDR
QFGEGEKVSILGTDPALNRVLLNGQAIASTSWGGDPNDPDSSSFNYSTLAPEVVGLMEVY
KTPEARIDEGSIGGTVIVNTRKPLDLERNTFTGTVTYGYNDRPDEGKPNASVLYSWKNQD
ETLGVLTSVMHSQRLLRRDGVEIFGYDNVAGAAFPPAVVGNNTGVFPTSINTALFQQTRK
RDGCSAALQWKPDSEFELNLTGPYVKESFDNYNQSRYGYWGSNPGDAQALGFENGVATSG

TFGDQSNTYLDGYLRNSEVTTGSIHLRTDWHGDGWNASSQVGYTSSQGGAERIYGIQIPQ
SGWL*
>P1 locus=Scaffold14:118414:119643:-
LLIGGVGVGGAMALPASEQQPPVPAPSQPQSAPASVQEPAPASPWVSTLDGIKVVGYQAS
LGKALNVKRNADAIIDAISAEDIGKFPDTNVAESLSRLSGITVDRQFGEGEKVSILGTDP
ALNRVLLNGQAIASTSWGGDPNDPDSSSFNYSTLAPEVVGLMEVYKTPEARIDEGSIGGT
VIVNTRKPLDLERNTFTGTVTYGYNDRPDEGKPNASVLYSWKNQDETLGVLTSVMHSQRL
LRRDGVEIFGYDNVAGAAFPPAVVGNNTGVFPTSINTALFQQTRKRDGCSAALQWKPDSE
FELNLTGPYVKESFDNYNQSRYGYWGSNPGDAQALGFENGVATSGTFGDQSNTYLDGYLR
NSEVTTGSIHLRTDWHGDGWNASSQVGYTSSQGGAERIYGIQIPQSGWL*
>P6d locus=Scaffold8:85797:88424:+
MAAGQEQQAPASTPSATTELDTITVTGYRASLEKSQSVKRAANSIVDAISAEDIGKFPDT
NAAESLAHVPGISVDRQFGEGEKVSINGTDPALNRVLLNGQTIASGDWGGNPSDTSGRTF
NYTLLSPEIIGLMEVYKTPEARIDEGSIGGTVIVHTRKPLDLPKNTIRGSVGYNYNNRSK
EGNPRGSALWSWKNDDETFGALISATHDKQDLARAGIEYFGYTTGDKIPPTATITGDGSN
VATARVPAGISSAFFQQTRERNGLQGALQWKPDENNEFNLTGIYIKGKYNNYSQARYVCP
ACNDDLKKVTRANVENGVVTSATVSDNTQGGVNDQPYAQMDTNYRESTVTTKSLNLRHDW
SGEKWVFTTQIGDTEATGGKNPEYLMKYLMQDGGYNYAFDGRNTAVNYDNGGAANWALPG
NPAGLAPGAETIPGTSPSVSPMQAGGIYYQKSKDQEKYFQWDASRDLALGPFNKLQFGYK
YINHDNGVDARGNRINTTDPVSLTQFNPGTTQSSLYDGLGASGDLTTWPTANLGAVRRYL
LAQPQGPYNTDFGGSFDVKEITQNVYTQLNFESGQWRGNVGVRLLDTTDKSEYWQSPDNG
QSYSRVAETHEYRKALPSFNVAYDVTDDAVLRFSVAKVMARPRYADLAGSFTVNSGNGNL
TASGGNPDLKPYESTNYDLAAEWYFAPSSMLSGEVFYRDISSYIVSTTVGEQRPATNLNP
AGLYQVTTPTNASDAKVKGASINYQQTFGLGFGLQANYTYAKADASTGLNLPYLSRDTYN
VIPYWEHGDWTVRVNYSYRSKYFTQLGRLASEDFADSYKQLDLTASYQITDYMGLTFGAT
NLLDSTYKLYSGTRDTPTAFYKNGRGYQAQLNFKF*
>P3b locus=Scaffold2:136056:138686:-
MAAGQEQQAPASTPSTTTQLDTITVTGYRASLEKSQSVKRAANSIVDAISAEDIGKFPDT
NAAESLAHVPGISVDRQFGEGEKVSINGTDPALNRVLLNGQTIASGDWGGNPSDTSGRTF
NYTLLSPEIIGLMEVYKTPEARIDEGSIGGTVIVHTRKPLDLPKNTIRGSVGYNYNDRSK
EGNPRGSALWSWKNDDETFGALISATRDEQDLARAGIEYFGYTTGKGIPPTATITGDGSN
IATARVPAGISSAFFQQTRERNGLQGALQWKPDENNEFNLTGIYIKGKYNNYSQARYVCP
ACNDDLKKVTRANVENGVVTSATVSDNTQGGVNDQPYAQMDTNYRESTVTTKSLNLRHDW
SGEKWVFTTQIGDTEATGGKNPEYLMKYLMQDGGYNYAFDGRNTAVNYDNGGAANWALPG
NPAGLAPGAETIPGTSPSVSPMQAGGIYYQKSKDQEKYFQWDASRDLALGPFNKLQFGYK
YINHDNGVDARGNRINTTDPVSLTQFNPGTTQSSLYDGLGASGDLTTWPTANLGAVRRYL
LAQPQGPYNTDFGGSFDVKEITQNVYTQLNFESGQWRGNVGVRLLDTTDKSEYWQSADNG
ASYSRVAETHDYRKALPSFNVAYDVTDDAVLRFSVAKVMARPRYGDLAGSFTVNSGNGNL
TASGGNPDLRPYESTNYDLAAEWYFAPSSMLSGEVFYRDISSYIVSTTVSEFRQADRRGN
EQGIYQITTPTNASDAKVKGASINYQQTFGLGFGLQANYTYAKADASTGLNLPYLSRDTY
NVIPYWEHGDWTVRVNYSYRSKYFTQLGRLASEDFADSYKQLDLTASYQITDYMGLTFGA
TNLLDSTYKLYSGTRDTPTAFYKNGRGYQAQLNFKF*
>P3c locus=Scaffold7:129945:132569:+
MAAGQEQQAPASTPSTTTQLDTITVTGYRASLEKSQSVKRAANSIVDAISAEDIGKFPDT

NAAESLAHIPGISVDRQFGEGEKVSINGTDPALNRVLLNGQTIASGDWGGNPSDTSGRTF
NYTLLSPEIIGLMEVYKTPEARIDEGSIGGTVIVHTRKPLDLPKNTIRGSVGYNYNDRSK
EGNPRGSALWSWKNDDETFGALISATHDEQDLARAGIEYFGYTTGDKIPPTATITGDGSN
VATARVPAGISSAFFQQTRERNGLQGALQWKPDENNEFNLTGIYIKGKYNNYSQARYVCP
ACNDDLKKVTRANVENGVVTSATVSDNTQGGVNDQPYAQMDTNYRESTVTTKSLNLRHDW
SGEKWVFTTQIGDTEATGGKNPEYLMKYLMQDGGYNYAFDGRNTAVNYDNGGAANWALPG
NPAGLAPGQQGKIPGTSTDIMQAGGIYYQKSKDQEKYFQWDASRDLALGPFNKLQFGYKY
INHDNGVDARGNRINTTDPVSLTQFNPGTTQSSLYDGLGASGDLTTWPTANLGAVRRYLL
AQPQGPYNTDFDGSFDVKEITQNVYTQLNFESGQWRGNVGVRLLDTTDKSEYWQSADNRD
SYSRVAETHEYRKALPSFNVAYDVTDDAVLRFSVAKVMARPRYADLAGSFTVNSGNGNLT
ASGGNPDLKPYESTNYDLAAEWYFAPSSMLSGEVFYRDISSYIVSTTVSQQLNPAPPALP
GIYQITTPTNASDAKVKGASINYQQTFGLGFGLQANYTYAKADASTGLNLPYLSRDTYNV
IPYWEHGDWTVRVNYSYRSKYFTQIGRLGSEDFADSYKQLDLTASYQITDYMGLTFGATN
LLDSTYKLYSGTRDTPTAFYKNGRGYQAQLNFKF*
>P4 locus=Scaffold2:332660:335287:+
MAAGQEQQAPASTPSATTELDTVTVTGYRASLEKSQSVKRAANSIVDAISAEDIGKFPDT
NAAESLAHVPGISVDRQFGEGEKVSINGTDPALNRVLLNGQTIASGDWGGNPSDTSGRTF
NYTLLSPEIIGLMEVYKTPEARIDEGSIGGTVIVHTRKPLDLPKNTIRGSVGYNYNDRSK
EGNPRGSALWSWKNDDETFGALISATRDEQDLARAGIEYFGYTTGKGIPPTATITGDGSN
IATARVPAGISSAFFQQTRERNGLQGALQWKPDENNEFNLTGIYIKGKYNNYSQARYVCP
ACNDDLKKVTRANVENGVVTSATVSDNTQGGVNDQPYAQMDTNYRESTVTTKSLNLRHDW
SGEKWVFTTQIGDTEATGGKNPEYLMKYLMQDGGYNYAFDGRNTAVNYDNGGAANWALPG
NPAGLAPGAETIPGTSPSVSPMQAGGIYYQKSKDQEKYFQWDASRDLALGPFNKLQFGYK
YINHDNGVDARGNRINTTDPVSLTQFNPGTTQSSLYDGLGASGDLTTWPTANLGAVRRYL
LAQPQGPYNTDFGGSFDVKEITQNVYTQLNFESGQWRGNVGVRLLDTTDKSEYWQSPDNG
QSYSRVAETHEYRKALPSFNVAYDVTDDAVLRFSVAKVMARPRYADLAGSFTVNSGNGNL
TASGGNPDLKPYESTNYDLAAEWYFAPSSMLSGEVFYRDISSYIVSTTVGEQRPATNLNP
AGLYQVTTPTNASDAKVKGASINYQQTFGLGFGLQANYTYAKADASTDLNLPYLSRDTYN
VIPYWEHGGWMVRVNYSYRSKYFTQIGRLGSEDFADSYKQLDLTASYQINDTMGLTFGAT
NLLDSTYKLYSGTRDTPTAFYKNGRGYQAQLNFKF*
>P5 locus=Scaffold5:108776:111406:-
MAAGQEQQAPASTPSTTTQLDTITVTGYRASLEKSQSVKRAANSIVDAISAEDIGKFPDT
NAAESLAHVPGISVDRQFGEGEKVSINGTDPALNRVLLNGQTIASGDWGGNPSDTSGRTF
NYTLLSPEIIGLMEVYKTPEARIDEGSIGGTVIVHTRKPLDLPKNTIRGSVGYNYNDRSK
EGNPRGSALWSWKNDDETFGALISATRDEQDLARAGIEYFGYTTGKGIPPTATITGDGSN
IATARVPAGISSAFFQQTRERNGLQGALQWKPDENNEFNLTGIYIKGKYNNYSQARYVCP
ACNDDLKKVTRANVENGVVTSATVSDNTQGGVNDQPYAQMDTNYRESTVTTKSLNLRHDW
SGEKWVFTTQIGDTEATGGKNPEYLMKYLMQDGGYNYAFDGRNTAVNYDNGGAANWALPG
NPAGLAPGAETIPGTSPSVSPMQAGGIYYQKSKDQEKYFQWDASRDLALGPFNKLQFGYK
YINHDNGVDARGNRINTTDPVSLTQFNPGTTQSSLYDGLGASGDLTTWPTANLGAVRRYL
LAQPQGPYNTDFGGSFDVKEITQNVYTQLNFESGQWRGNVGVRLLDTTDKSEYWQSADNG
ASYSRVAETHDYRKALPSFNVAYDVTDDAVLRFSVAKVMARPRYGDLAGSFTVNSGNGNL
TASGGNPDLRPYESTNYDLAAEWYFAPSSMLSGEVFYRDISSYIVSTTVSEFRQADRRGN
EQGIYQITTPTNASDAKVKGASINYQQTFGLGFGLQANYTYAKADASTGLNLPYLSRDTY

NVIPYWEHGDWTVRVNYSYRSKYFTQLGRLASEDFADSYKQLDLTASYQITDYMGLTFGA
TNLLDSTYKLYSGTRDTPTAFYKNGRGYQAQLNFKF*
>P6 locus=Scaffold2:174767:177394:-
MAAGQEQQAPASTPSATTELDTITVTGYRASLEKSQSVKRAANSIVDAISAEDIGKFPDT
NAAESLAHVPGISVDRQFGEGEKVSINGTDPALNRVLLNGQTIASGDWGGNPSDTSGRTF
NYTLLSPEIIGLMEVYKTPEARIDEGSIGGTVIVHTRKPLDLPKNTIRGSVGYNYNNRSK
EGNPRGSALWSWKNDDETFGALISATHDKQDLARAGIEYFGYTTGDKIPPTATITGDGSN
VATARVPAGISSAFFQQTRERNGLQGALQWKPDENNEFNLTGIYIKGKYNNYSQARYVCP
ACNDDLKKVTRANVENGVVTSATVSDNTQGGVNDQPYAQMDTNYRESTVTTKSLNLRHDW
SGEKWVFTTQIGDTEATGGKNPEYLMKYLMQDGGYNYAFDGRNTAVNYDNGGAANWALPG
NPAGLAPGAETIPGTSPSVSPMQAGGIYYQKSKDQEKYFQWDASRDLALGPFNKLQFGYK
YINHDNGVDARGNRINTTDPVSLTQFNPGTTQSSLYDGLGASGDLTTWPTANLGAVRRYL
LAQPQGPYNTDFGGSFDVKEITQNVYTQLNFESGQWRGNVGVRLLDTTDKSEYWQSPDNG
QSYSRVAETHEYRKALPSFNVAYDVTDDAVLRFSVAKVMARPRYADLAGSFTVNSGNGNL
TASGGNPDLKPYESTNYDLAAEWYFAPSSMLSGEVFYRDISSYIVSTTVGEQRPATNLNP
AGLYQVTTPTNASDAKVKGASINYQQTFGLGFGLQANYTYAKADASTGLNLPYLSRDTYN
VIPYWEHGDWTVRVNYSYRSKYFTQLGRLASEDFADSYKQLDLTASYQITDYMGLTFGAT
NLLDSTYKLYSGTRDTPTAFYKNGRGYQAQLNFKF*
>P7 locus=Scaffold6:128671:131295:-
MAAGQEQQAPASTPSATTQLDTITVTGYRASLEKSQSVKRAANSIVDAISAEDIGKFPDT
NAAESLAHIPGISVDRQFGEGEKVSINGTDPALNRVLLNGQTIASGDWGGNPTDTSGRTF
NYTLLSPEIIGLMEVYKTPEARIDEGSIGGTVIVHTRKPLDLPKNTIRGSVGYNYNDRSR
EGNPRGSALWSWKNDDETFGALISATRDEQDLARAGIEYFGYTTGKGIPPTATITGDGSN
IATARVPAGISSAFFQQTRERNGLQGALQWKPDENNEFNLTGIYIKGKYNNYSQARYVCP
ACNDDLKKVTRANVENGVVTSATVSDNTQGGVNDQPYAQMDTNYRESTVTTKSLNLRHDW
SGEKWVFTTQIGDTEATGGKNPEYLMKYLMQDGGYNYAFDGRNTAVNYDNGGAANWALPG
NPAGLAPGQQGKIPGTDTDIMQAGGIYYQKSKDQEKYFQWDASRDLALGPFNKLQFGYKY
INHDNGVDARGNRINTTDPVSLTQFNPGTTQSSLYDGLGASGDLTTWPTADLAAVRRYLL
SQPQGPYSIDHGASFDVKEITQNVYTQLNFESGQWRGNVGVRLLDTTDKSEYWQSADNGD
SYSRVAETHEYRKALPSFNVAYDVTDDAVLRFSVAKVMARPRYADLAGSFTVNSGNGNLT
ASGGNPDLKPYESTNYDLAAEWYFAPSSMLSGEVFYRDISSYIVSTTVSQQLNPAPPALP
GIYQITTPTNASDAKVKGASINYQQTFGLGFGLQANYTYAKADASTGLNLPYLSRDTYNV
IPYWEHGDWTVRVNYSYRSKYFTQIGRLGSEDFADSYKQLDLTASYQITDYMGLTFGATN
LLDSTYKLYSGTRDTPTAFYKNGRGYQAQLNFKF*
>P8 locus=Scaffold4:126928:129558:-
MAAGQEQQAPASTPSTTTQLDTITVTGYRASLEKSQSVKRAANSIVDAISAEDIGKFPDT
NAAESLAHVPGISVDRQFGEGEKVSINGTDPALNRVLLNGQTIASGDWGGNPSDTSGRTF
NYTLLSPEIIGLMEVYKTPEARIDEGSIGGTVIVHTRKPLDLPKNTIRGSVGYNYNDRSK
EGNPRGSALWSWKNDDETFGALISATRDEQDLARAGIEYFGYTTGKGIPPTATITGDGSN
IATARVPAGISSAFFQQTRERNGLQGALQWKPDENNEFNLTGIYIKGKYNNYSQARYVCP
ACNDDLKKVTRANVENGVVTSATVSDNTQGGVNDQPYAQMDTNYRESTVTTKSLNLRHDW
SGEKWVFTTQIGDTEATGGKNPEYLMKYLMQDGGYNYAFDGRNTAVNYDNGGAANWALPG
NPAGLAPGAETIPGTSPSVSPMQAGGIYYQKSKDQEKYFQWDASRDLALGPFNKLQFGYK
YINHDNGVDARGNRINTTDPVSLTQFNPGTTQSSLYDGLGASGDLTTWPTANLGAVRRYL

LAQPQGPYNTDFGGSFDVKEITQNVYTQLNFESGQWRGNVGVRLLDTTDKSEYWQSADNG
ASYSRVAETHDYRKALPSFNVAYDVTDDAVLRFSVAKVMARPRYGDLAGSFTVNSGNGNL
TASGGNPDLRPYESTNYDLAAEWYFAPSSMLSGEVFYRDISSYIVSTTVSEFRQADRRGN
EQGIYQITTPTNASDAKVKGASINYQQTFGLGFGLQANYTYAKADASTGLNLPYLSRDTY
NVIPYWEHGDWTVRVNYSYRSKYFTQLGRLASEDFADSYKQLDLTASYQITDYMGLTFGA
TNLLDSTYKLYSGTRDTPTAFYKNGRGYQAQLNFKF*
>P9a locus=Scaffold5:129748:130080:+
LSRPSLPYNSRNAYNISPYYEQGKWSARVNLGWRSEYLTQIGRLNGQQMTDAFTQVDASF
GYQATERLRVALEATNLLDETYFSYIGNKNQPYYIYKNGRSFMLSLNFKL*
>P9b locus=Scaffold3:295142:296371:-
LLIGGVGVGGAMALPASEQQPPVPAPSQPQSAPASVQEPAPASPWVSTLDGIKVVGYQAS
LGKALNVKRNADAIIDAISAEDIGKFPDTNVAESLSRLSGITVDRQFGEGEKVSILGTDP
ALNRVLLNGQAIASTSWGGDPNDPDSSSFNYSTLAPEVVGLMEVYKTPEARIDEGSIGGT
VIVNTRKPLDLERNTFTGTVTYGYNDRPDEGKPNASVLYSWKNQDETLGVLTSVMHSQRL
LRRDGVEIFGYDNVAGAAFPPAVVGNNTGVFPTSINTALFQQTRKRDGCSAALQWKPDSE
FELNLTGPYVKESFDNYNQSRYGYWGSNPGDAQALGFENGVATSGTFGDQSNTYLDGYLR
NSEVTTGSIHLRTDWHGDGWNASSQVGYTSSQGGAERIYGIQIPQSGWL*
>P9c locus=Scaffold2:260199:262823:+
MAAGQEQQAPASTPSTTTQLDTITVTGYRASLEKSQSVKRAANSIVDAISAEDIGKFPDT
NAAESLAHIPGISVDRQFGEGEKVSINGTDPALNRVLLNGQTIASGDWGGNPSDTSGRTF
NYTLLSPEIIGLMEVYKTPEARIDEGSIGGTVIVHTRKPLDLPKNTIRGSVGYNYNDRSK
EGNPRGSALWSWKNDDETFGALISATHDEQDLARAGIEYFGYTTGDKIPPTATITGDGSN
VATARVPAGISSAFFQQTRERNGLQGALQWKPDENNEFNLTGIYIKGKYNNYSQARYVCP
ACNDDLKKVTRANVENGVVTSATVSDNTQGGVNDQPYAQMDTNYRESTVTTKSLNLRHDW
SGEKWVFTTQIGDTEATGGKNPEYLMKYLMQDGGYNYAFDGRNTAVNYDNGGAANWALPG
NPAGLAPGQQGKIPGTSTDIMQAGGIYYQKSKDQEKYFQWDASRDLALGPFNKLQFGYKY
INHDNGVDARGNRINTTDPVSLTQFNPGTTQSSLYDGLGASGDLTTWPTANLGAVRRYLL
AQPQGPYNTDFDGSFDVKEITQNVYTQLNFESGQWRGNVGVRLLDTTDKSEYWQSADNRD
SYSRVAETHEYRKALPSFNVAYDVTDDAVLRFSVAKVMARPRYADLAGSFTVNSGNGNLT
ASGGNPDLKPYESTNYDLAAEWYFAPSSMLSGEVFYRDISSYIVSTTVSQQLNPAPPALP
GIYQITTPTNASDAKVKGASINYQQTFGLGFGLQANYTYAKADASTGLNLPYLSRDTYNV
IPYWEHGDWTVRVNYSYRSKYFTQIGRLGSEDFADSYKQLDLTASYQITDYMGLTFGATN
LLDSTYKLYSGTRDTPTAFYKNGRGYQAQLNFKF*
>P9d locus=Scaffold4:245404:246498:-
VSTLDGIKVVGYQASLGKALNVKRNADAIIDAISAEDIGKFPDTNVAESLSRLSGITVDR
QFGEGEKVSILGTDPALNRVLLNGQAIASTSWGGDPNDPDSSSFNYSTLAPEVVGLMEVY
KTPEARIDEGSIGGTVIVNTRKPLDLERNTFTGTVTYGYNDRPDEGKPNASVLYSWKNQD
ETLGVLTSVMHSQRLLRRDGVEIFGYDNVAGAAFPPAVVGNNTGVFPTSINTALFQQTRK
RDGCSAALQWKPDSEFELNLTGPYVKESFDNYNQSRYGYWGSNPGDAQALGFENGVATSG
TFGDQSNTYLDGYLRNSEVTTGSIHLRTDWHGDGWNASSQVGYTSSQGGAERIYGIQIPQ
SGWL*
>P10 locus=Scaffold2:197299:198528:-
LLIGGVGVGGAMALPASEQQPPVPAPSQPQSAPASVQEPAPASPWVSTLDGIKVVGYQAS
LGKALNVKRNADAIIDAISAEDIGKFPDTNVAESLSRLSGITVDRQFGEGEKVSILGTDP

ALNRVLLNGQAIASTSWGGDPNDPDSSSFNYSTLAPEVVGLMEVYKTPEARIDEGSIGGT
VIVNTRKPLDLERNTFTGTVTYGYNDRPDEGKPNASVLYSWKNQDETLGVLTSVMHSQRL
LRRDGVEIFGYDNVAGAAFPPAVVGNNTGVFPTSINTALFQQTRKRDGCSAALQWKPDSE
FELNLTGPYVKESFDNYNQSRYGYWGSNPGDAQALGFENGVATSGTFGDQSNTYLDGYLR
NSEVTTGSIHLRTDWHGDGWNASSQVGYTSSQGGAERIYGIQIPQSGWL*
>GIV locus=Scaffold10:9854:10948:-
VSTLDGIKVVGYQASLGKALNVKRNADAIIDAISAEDIGKFPDTNVAESLSRLSGITVDR
QFGEGEKVSILGTDPALNRVLLNGQAIASTSWGGDPNDPDSSSFNYSTLAPEVVGLMEVY
KTPEARIDEGSIGGTVIVNTRKPLDLERNTFTGTVTYGYNDRPDEGKPNASVLYSWKNQD
ETLGVLTSVMHSQRLLRRDGVEIFGYDNVAGAAFPPAVVGNNTGVFPTSINTALFQQTRK
RDGCSAALQWKPDSEFELNLTGPYVKESFDNYNQSRYGYWGSNPGDAQALGFENGVATSG
TFGDQSNTYLDGYLRNSEVTTGSIHLRTDWHGDGWNASSQVGYTSSQGGAERIYGIQIPQ
SGWL*
>GV locus=Scaffold6:77733:80357:-
MAAGQEQQAPASTPSTTTQLDTITVTGYRASLEKSQSVKRAANSIVDAISAEDIGKFPDT
NAAESLAHIPGISVDRQFGEGEKVSINGTDPALNRVLLNGQTIASGDWGGNPSDTSGRTF
NYTLLSPEIIGLMEVYKTPEARIDEGSIGGTVIVHTRKPLDLPKNTIRGSVGYNYNDRSK
EGNPRGSALWSWKNDDETFGALISATHDEQDLARAGIEYFGYTTGDKIPPTATITGDGSN
VATARVPAGISSAFFQQTRERNGLQGALQWKPDENNEFNLTGIYIKGKYNNYSQARYVCP
ACNDDLKKVTRANVENGVVTSATVSDNTQGGVNDQPYAQMDTNYRESTVTTKSLNLRHDW
SGEKWVFTTQIGDTEATGGKNPEYLMKYLMQDGGYNYAFDGRNTAVNYDNGGAANWALPG
NPAGLAPGQQGKIPGTSTDIMQAGGIYYQKSKDQEKYFQWDASRDLALGPFNKLQFGYKY
INHDNGVDARGNRINTTDPVSLTQFNPGTTQSSLYDGLGASGDLTTWPTANLGAVRRYLL
AQPQGPYNTDFDGSFDVKEITQNVYTQLNFESGQWRGNVGVRLLDTTDKSEYWQSADNRD
SYSRVAETHEYRKALPSFNVAYDVTDDAVLRFSVAKVMARPRYADLAGSFTVNSGNGNLT
ASGGNPDLKPYESTNYDLAAEWYFAPSSMLSGEVFYRDISSYIVSTTVSQQLNPAPPALP
GIYQITTPTNASDAKVKGASINYQQTFGLGFGLQANYTYAKADASTGLNLPYLSRDTYNV
IPYWEHGDWTVRVNYSYRSKYFTQIGRLGSEDFADSYKQLDLTASYQITDYMGLTFGATN
LLDSTYKLYSGTRDTPTAFYKNGRGYQAQLNFKF*

Tree file for PXO_01644 (Supports Supplemental Figure S3A)

(P9a:0.00000001,(C6:0.00000001,C5:0.00000002)311:0.00000001,((GIV:0.00000001,((C3:0.00000028,C1:0.00000002)24:0.00000014,(C7:0.00000001,(C4:0.00000015,(P1:0.00000001,(P9b:0.00000027,P10:0.00000016)10:0.00000014)12:0.00000026)5:0.00000030)2:0.00000022)10:0.00000001)14:0.00000021,(C2:0.00000014,(P9d:0.00000001,((GV:0.00000001,(P3c:0.00000001,P9c:0.00000001)264:0.00000001)718:0.00363270,(P7:0.01248022,((P8:0.00000001,(P3b:0.00000001,P5:0.00000001)311:0.00000001)887:0.01154861,(P4:0.00831693,(P6d:0.00000001,(P6:0.00000001,PXO99A:0.00000001)314:0.00000001)951:0.00788680)843:0.01222070)787:0.02014222)689:0.00360241)965:1.04776562)44:0.00000001)12:0.00000014)1000:8.17496144);
