# Supplementary material for: Reciprocal adaptation of rice and Xanthomonas oryzae pv. oryzae: cross-species 2D GWAS reveals the underlying genetics
Source: Plant Cell. 2021 Jun 2;33(8):2538–61. doi: 10.1093/plcell/koab146 (PMC8408478; doi:10.1093/plcell/koab146)
Supplement: koab146_Supplementary_Data [file koab146_supplementary_data.zip › tpc.01058.2020-s08.pdf]

Reciprocal adaptation of rice and *Xanthomonas oryzae* pv. *oryzae*: cross-species two-dimensional GWAS reveals the underlying genetics

Fan Zhang, Zhiqiang Hu, Zhichao Wu, Jialing Lu, Yingyao Shi, Jianlong Xu, Xiyin Wang, Jinpeng Wang, Fan Zhang, Mingming Wang, Xiaorong Shi, Yanru Cui, Casiana Vera Cruz, Dalong Zhuo, Dandan Hu, Min Li, Wensheng Wang, Xiuqin Zhao, Tianqing Zheng, Binying Fu, Jauhar Ali, Yongli Zhou, Zhikang Li

Corresponding authors: Zhikang Li, lizhikang@caas.cn, and Yongli Zhou, zhouyongli@caas.cn

Review timeline:

TPC2019-RA-00500	Submission received:<br>1st Decision:	July 16, 2019<br>Aug. 23, 2019 *manuscript declined*
TPC2020-LSB-00368	Submission received:<br>1st Decision:	May 11, 2020<br>June 11, 2020 *manuscript declined*
**TPC2020-LSB-01058**	Submission received:<br>1st Decision:	Dec. 17, 2020<br>Feb. 15, 2021*revision requested*
**TPC2020-LSB-01058R1**	1st Revision received:<br>2nd Decision:<br>Final acceptance:	April 6, 2021<br>April 26, 2021 *acceptance pending, sent to science editor*<br>May 15, 2021

REPORT: (The report shows the major requests for revision and author responses. Minor comments for revision and miscellaneous correspondence are not included. The original format may not be reflected in this compilation, but the reviewer comments and author responses are not edited, except to correct minor typographical or spelling errors that could be a source of ambiguity.)

TPC2019-RA-00500 1st Editorial decision – *declined* **Aug. 23, 2019**

Thank you for choosing to send your manuscript entitled "The complex genetic interaction systems leading to stabilizing selection underlying the coevolution of rice and *Xanthomonas oryzae* pv. *oryzae* revealed by two-dimensional GWAS" for consideration at The Plant Cell. Your submission has been evaluated by members of the editorial board as well as expert reviewers in your field, and we regret to inform you that we are not able to recommend publication of this manuscript. We have not made this decision lightly. We have had input from multiple scientists, and have solicited post-review comments as well. Our present policy is to offer streamlined decisions and to not advise on the direction of the work by requesting extensive modifications or substantial additional experiments.

During the post-review consultation session, we also agreed that if you could address the major points raised by the reviewers by new experiments, we would welcome a resubmission. This would be treated as a new submission, but we would attempt to use at least some of the same reviewers. Nevertheless, reviewers will be asked to assess as a new manuscript (i.e. are the claims fully supported by the data; do the results presented move the field forward), and not only whether previous reviewer comments have been addressed.

This decision is based on two key factors. First, the pathogen GWAS utilizes a limited set of isolates and it is not clear about the power of the GWAS to identify associations. There are also significant concerns about the FDR analysis in the XOO x Rice interaction test both with regard to the appropriate threshold and an absence of a null distribution of p-values. The reviewers give numerous suggestions for how to strengthen these sections.

The reviewers also noted that the introduction did not delve into the existing literature on the genomics of interactions between host and pathogens within the plant literature. The reviewers provided guidance literature areas that are directly linked to the manuscript and should be discussed/included in the introduction and discussion.

It will be important to convince the editors and reviewers that the study adds significant new understanding of mechanisms or processes or presents an exceptional resource for the community (i.e. as a large-scale biology article) and that the major claims made are fully justified by the data presented. This includes careful consideration and explanation of the various controls used in experiments, the extent and manner of replication, and the statistical analyses used. Sampling methods and nature of "biological replicates" should be described precisely (i.e. different plants, parts of plants, pooled tissue, independent pools of tissue, sampled at

different times, etc.), along with a clear description of and rationale for any statistical analyses conducted. The reader should know exactly what was sampled; what forms the basis of the calculation of any means and other statistical variables and parameters reported. This is also necessary to ensure that proper statistical analysis was conducted.

-- Reviewer comments:

[Reviewer comments shown below along with author responses]

TPC2020-LSB-00368 Submission received **May 11, 2020**

Reviewer comments on previously declined manuscript and **author responses:**

Reviewer #1:

The authors present a genetic association study of rice lesions caused by *Xanthomonas oryzae* pv. *Oryzae* (*Xoo*), identifying associated variants in both rice and *Xoo* and genetic interactions between species. I was asked to evaluate the GWAS methods, so did not consider the rest of the paper.

Overall, the experimental design is good: as I understand, for the *Xoo* association analysis, all strains were inoculated simultaneously on the same rice plants, with this design replicated over 73 different rice accessions. Only 23 strains were included which is very small for association, but understandable given the constraints of an already large experiment. For the rice association, 4 *Xoo* strains were inoculated on each of the 701 rice accessions, with adequate replication of each accession in both experiments. For the *Xoo* association, the association analysis searched for any SNP that was commonly associated with legion size across multiple rice accessions. In rice, the association analysis searched for any SNP associated with legion size from any of the 4 target *Xoo* strains, in any sub-population of rice. The interaction association test looked at a priori loci, or those identified above, and tested for pairwise interactions between the *Xoo* SNPs and rice SNPs.

Major revisions:

The combined association score appears to be an attempt to prioritize *Xoo* SNPs that have effects in multiple rice lines, while discounting associations from particular lines if too many SNPs were association in that line. While pretty ad-hoc, this seems reasonable. But it is hard to tell from what is presented if it really works. There are two key thresholds: $p < 0.001$, and CAS > 0.03. The key statement: "This threshold was determined by considering the ranks of the known virulence-related genes" needs to be elaborated. How were the ranks considered? Were both thresholds chosen to maximize the enrichment of the known genes at the top of the list? I also think that permutations of the genotype data (see Abney 2015 or Chen and Palmer 2013) would be useful to demonstrate that the number of false positives at these thresholds isn't too great.

A: Thank you for the insightful suggestion. We have revised the description of parameter selection in the Method section: "This threshold was determined by considering the ranks of the known and candidate virulence-related genes. Specifically, we first utilized three different *p*-value cutoffs (1e-3, 1e-4 and 1e-5) to calculate the CAS scores; next, by manually exploring literatures in PubMed database (to see if a gene is reported as a pathogen gene in other bacteria), we manually annotated 113 genes with CAS over 0.1 at any *p*-value cutoff; then, we assessed the performances of combinations of *p*-value and CAS score cutoffs by assuming that all the candidate pathogen genes are true positives, the combination of *p*-value cut-off at 0.001 and CAS cutoff at 0.3 gives the highest accuracy (23 candidate disease gene detected with a specificity of 0.49), defined as the average value of sensitivity and specificity at the genic level."

We carried out genotype permutation analysis to estimate the false discovery rate. Specifically, we shuffled the genotypes (exchange "whole" genotypes among the 23 bacteria) and performed the CAS-based GWAS. We did 10,000 permutations and for each permutation, 15.0±8.0 SNPs could be flagged as significant (as described previously in the method section: CAS (calculated at 0.001) >0.3, detected times ≥2 and minimum SNP <0.0001), which suggests an SNP-level false discovery rate of 15/86 (the significant SNP number detected in this paper) = 0.17. Moreover, the permutation also suggested that the probability of observing ≥86 significant SNPs is less than 0.0001. We revised the manuscript accordingly to integrate the permutation results.

I have two concerns with the genomic-interaction analysis.

1. Power. The two-way ANOVA appears to be missing terms for rice line (72df) and Xoo strain (22df). These terms should be included (random effects would be preferred, but including as fixed effects is OK for the interaction test). This should actually increase power for the interaction test. The FDR method should be specified, and a FDR threshold of <0.0001 is extremely conservative. With FDR, it is reasonable to use thresholds of 0.1 or even greater in a study like this (allowing 10% false-positives seems reasonable if it means finding many more true interactions).

A: Thank you for the insightful suggestion. We have added additional covariates in the linear model as suggested. This is described in detail in the Method part. The whole interaction result section is rewritten based on the new analysis. As this is the first time we can "see" the global cross-species interaction, we prefer not to exaggerate the interactions, and determine to use the Bonferroni-adjusted *p*-value (by all possible tests (16607 *Xoo* coding variants with MAF>0.05 *5379674 rice variants with MAF>0.05) instead of the number of tests we conducted here) <0.05 as the threshold.

2. Interpretation of interactions. There is no indication of any data transformation applied. But if the variance in lesion size increases with the mean, it is likely that there are multiplicative interactions among the Rice SNPs and *Xoo* SNPs. Figure SF11 does seem to show increased variance for strains causing larger lesions (as would be expected based on a growth-model of lesions through time). Multiplicative interactions are interactions (at least on the measured scale), but are perhaps less interesting that other types of statistical interaction (because they can be thought of as simply additive effects on growth rates). This may be why so many interactions are found. Does the number of interactions greatly change after log or sqrt transformation of the data? A figure showing some key interactions would be helpful.

A: We understand that data of disease incidences measured as lesion lengths do not have measurement errors that follow the normal distribution required for GWAS and interaction analyses because the variance and mean for LL are correlated. In our cases, the mean lesion lengths from more than 30 inoculated leaves of each accession, instead of LL from a single leaf, were used as the input data in GWAS and interaction analyses. According to the Central Limit Theorem of the mathematical statistics, the mean LLs should seriously violate the assumption for normality (independence of mean from variance of LL) for GWAS. Indeed, as suggested by the reviewer, we have conducted another interaction association analysis based on log-transformed phenotypes (LL). This new analysis resulted in 992,171 significant interactions, including all previously detected significant interactions by non-transformed data, which is about four times of the number without log transformation. This is described in the discussion session. To better illustrate the results, we generated a heatmap figure to show key gene-for-gene interactions detected (Figure 7B).

Figure S9 shows that almost all p-value distributions for the rice GWAS are inflated (many more SNPs strongly associated that seems reasonable). Therefore, I would recommend doing genotype permutations (holding phenotype and Kinship constant) or MVNpermute-based thresholds for these GWASs, rather than the Bonferroni threshold used.

A: Thanks for the suggestion. We conducted the permutation tests using the newly generated multivariate normal samples by the MVNpermute package in R for the rice GWAS. The results showed that the thresholds based on permutations at FDR0.05 were less stringent than those Bonferroni thresholds (please see the following Table). Thus, the current thresholds used in this study ensured that the false-discovery-rates were no more than 0.0179 for each of the four *Xoo* strains. Meanwhile, we have used the effective number of SNPs rather than the total number of SNPs in the Bonferroni-based threshold to reduce the false negatives.

At present, many rice GWAS papers used the Bonferroni method to determine the threshold. We hope the reviewer will agree with the Bonferroni threshold used in this study.

Minor issues:

193-200: The rice SNPs identified by GWAS are annotated for functional effects using snpeff. This is not really justified because GWAS hits can't be considered the causal loci themselves unless all variants are included in the analysis. That is clearly not true here because low-frequency SNPs and all structural variants are excluded.

A: We have rewritten the relative description of the Results section in the resubmitted manuscript.

172: I don't think Figure 1B is the right reference here.

A: We have deleted the mentioned reference in the resubmitted manuscript.

337-340: This sentence isn't clear and may not be complete.

A: This sentence was rewritten in the resubmitted manuscript.

Reviewer #2:

The manuscript by Zhang et al. examines the genetic basis of rice - *Xanthomonas oryzae* pv. *oryzae* (Xoo) interactions using a two-way association mapping to cover both host and pathogen loci. The mapping populations included 701 rice accessions and 23 Xoo strains, respectively. Xoo strains were of diverse geographic origins and showed evidence for population substructure and likely gene flow.

Major comments:

The introduction does not do justice to the literature context and is confusing in some sections. E.g. L52-56 seems to make little sense to me. Citing only literature dating from the 70s seems inappropriate here. I also don't understand what "genome-to-genome interaction" (L56) should mean exactly. Clearly, genomes don't interact physically so a reader should be properly introduced to the concept. If the authors refer to the action of co-evolution, the interaction is mediated by reciprocal selection pressure and the interaction is at the phenotypic level. L57 is also wrong in the sense that there's a wealth of plant and pathogen loci with well-understood roles in resistance/disease.

A: Thanks for the comment. We were aware of our inappropriate introduction. We have also rewritten the introduction and cited the recent and high impact references to update the development of gene-for-gene theory for plant and pathogen interactions in the resubmit manuscript.

The introduction also fails to properly introduce the concept underlying association mapping (in particular on both host and pathogen). There is no information why the chosen rice cultivars and *Xoo* strains are appropriate to address major unanswered questions. There are numerous GWAS studies on host and pathogens (see Botrytis, Zymoseptoria, Parastagnospora, Magnaporthe systems etc.). The rationale for using GWAS and its limitations should also be clearly introduced and then later discussed.

A: Thanks for the suggestion. We have added the relevant information and rewritten the whole Introduction section in the resubmitted manuscript.

The concept of "co-evolution" is not properly introduced and applied in contexts where there is little evidence for co-evolution. First of all, the most important references in the literature on co-evolution should be cited. Second, co-evolution is defined as reciprocal selection pressure in species interactions. Clearly, *Xoo* was selected to become more virulent on rice. But because of breeding, the gene pool of rice does not respond directly to selection pressure of the pathogen. There may be breeding for resistance but this is clearly not fitting the broadly accepted notion of co-evolution. Hence, the use of "co- evolution" throughout the manuscript should be carefully revised.

A: Thanks for the comment. We have replaced the “co-evolution” with “arms-races” in the resubmitted manuscript.

Association mapping performed with only 23 *Xoo* strains is extremely low. At this population size, chance associations largely overwhelm true signals of phenotype-genotype associations. The low sample size is compounded by genetic substructure among the 23 strains and likely clonal reproduction. Unless the authors can provide a detailed rationale why it would be appropriate to perform association mapping with this population, this section of the manuscript may have to be omitted. Important factors to investigate and justify include levels of recombination, patterns of LD, the choice of proper regression models, etc. Please see some excellent reviews by J. Bergelson, D. Weigel or M. Nordborg on these issues and revise accordingly.

A: Thanks for your comment. We agree that the sample size of 23 is small for a GWAS. Because of the small sample size, it would be much more difficult to detect significant loci under a given threshold. Thus, false negatives, instead of false positives, would be a more serious problem in this study. However, the multiple phenotypes of different rice accessions we had here may improve the possibility of identifying true signals. The number of events here is 23*73=1679, which is even higher than most plant single- phenotype GWAS (N plants * 1 = N events). Most importantly, our new permutation analysis suggested that the probability of observing ≥86 (the number we detected here) significant SNPs in a random CAS-GWAS is less than 0.0001. The false discovery rate was estimated to be 0.17, which means 71 out of the 86 are true signals. We think the false discovery rate is reasonable here, considering our purpose here is to identify potential candidate *Xoo* gene for the subsequent interaction analysis. Moreover, in theinteraction analysis, we also integrated additional SNPs (both SNPs in known virulence gene and undetected SNPs in a detected gene with any SNP detected here).

The authors propose a "summary score" to deal with low sample size (and probably a lack of truly significant associations). From the details I see in the methods about the "combined association score" (or CAS), I cannot follow how this approach avoids the significant problem of false positives due to population structure, low sample size or both. The authors would either need to cite a study analyzing these issues, generate some simulations that would allow a reader to understand how CAS addresses the problem or choose different summary statistics.

A: Thanks for the insightful comments. This is also pointed out by reviewer #1. We have carried out additional permutation analysis, which suggests the CAS method does capture truly significant associations (*p*<0.0001). Please refer to the answer to the reviewer's Q1 for a detailed explanation.

All rice loci mapped in this study were sometimes called "*R*-genes". *R* genes are typically defined as having specific properties in terms of how these confer resistance to pathogens. *R* genes also often encode convergent protein architectures (NLR, etc.). However, there are many loci that may segregate variation in resistance to pathogens that have no relationship to *R*-genes.

A: Thanks for your suggestion. We have clearly defined the genes identified by GWAS in our paper into two types: resistance (*R*) genes and quantitative resistance (*QR*) genes. In the resubmitted manuscript, *R* genes are noted as those detected known *R* genes or genes with receptor-like kinase domains or with NBS-ARC and LRR domains based on the MSU 7.0 version annotation of Nipponbare reference genome IRGSP 1.0, while the other detected genes were designated as the QR genes.

The authors refer multiple times to stabilizing selection but stabilizing selection does not equal balancing selection. Only the latter would maintain polymorphism. Stabilizing selection occurs when a phenotypic trait is selected to remain at an intermediary optimum. A population under stabilizing selection may well be monomorphic. Balancing selection maintains polymorphism by favoring e.g. heterozygotes or two distinct alleles (i.e. the classic host- pathogen trench warfare).

A: Thanks for the comment. We have replaced the "stabilizing selection" with "balancing selection" in the resubmitted manuscript.

Tajima's D tests among *Xoo* strains is not properly justified. The recent demographic history and levels of admixture influence Tajima's D. How were these demographic factors controlled for in the subpopulation comparisons?

A: Thank you. In order to highlight our approach for the identification of virulence genes of *Xoo*, we have deleted the relevant results about Tajima's *D* tests in the resubmitted manuscript.

dN/dS analyses were originally designed to test for differences in substitution rates among non-recombining lineages. There is significant debate how appropriate such tests are for within-species comparisons. Please devise proper arguments why in this system dN/dS is not leading to false positives or the analog pN/pS.

A: Thank you. We have deleted the relevant results in the resubmitted manuscript.

Testing for SNP interactions between rice and *Xoo* populations: How was the threshold of $p < 0.0001$ justified? The multiple comparison correction must take into account the actual number of tests (all tested rice SNPs vs. all tested *Xoo* SNPs). There are ~10 million interactions tested (1909 x 5432 SNPs), so the threshold should be much lower (0.05/1e7) for a conservative threshold. Judging from Figure 7, there might be only a handful of interactions that pass such a threshold.

A: Thank you. We have revised the interaction detection strategy. In the resubmitted manuscript, we are using a Bonferroni-adjusted *p*-value (by all possible tests (16607 *Xoo* coding variants with MAF>0.05 *5379674 rice variants with MAF>0.05) instead of the number of tests we conducted here) <0.05 as the threshold. This is also pointed out by reviewer #1. Please refer to the answer to the reviewer's Q2 for a detailed explanation.

The discussion could be much more streamlined by focusing on the major and most robust outcomes. The study should also be placed into a broader literature context. Given the fact that "co-evolution" is mentioned multiple times, a critical view on these processes in agricultural systems would be warranted. Also, how does our new understanding of loci controlling the rice- *Xoo* shape our bigger understanding of plant-pathogen interactions?

A: Thanks for the comment. We have streamlined our discussion focusing on two major and most robust outcomes on the observed patterns and mechanistic of the coevolution between rice and *Xoo* at the population genomic level: (1) the correspondences between rice and *Xoo* in number and diversity of detected many rice R-genes/virulence genes of *Xoo*, as a result of their coevolutionary mechanisms; and

(2) The genome-wide interactions between rice *R*-genes and virulence gene of *Xoo* and the inferred underlying mechanisms (interpretations), which was rewritten and summarized in the 'Discussion' of the resubmitted MS.

Minor comments:

l42: genomes of hosts and pathogens don't interact.

A: Thanks a lot. We have corrected the description in the resubmitted manuscript.

l60-61: multiple references missing.

A: Thanks for the message. We now added the references in the resubmitted manuscript.

l121: I had difficulties finding an explanation in the methods how the significance was exactly tested. I might obviously have missed something.

A: We applied hypergeometric distribution to test the enrichment significance of SNP density. Now the hypergeometric test method was provided in the "Statistical analysis" of the Methods section in the resubmitted manuscript.

l201: What are "resistant SNPs"? First, there's not a necessary causal link. Also "resistance" could only be mediated by an allele and not the locus (SNP).

A: As suggested, we revised the description in the resubmitted manuscript.

<u>Reviewer #3:</u>

This manuscript describes a large set of sequencing data and represents a significant amount of work.

Strengths of the paper:

The interaction between rice and *Xanthomonas oryzae* pv. *oryzae* (*Xoo*) is a model system for understanding the coevolution between crop plants and their bacterial pathogens. The mechanisms with which bacterial pathogens evade detection by host R genes remains an interesting question. New mechanisms will almost certainly be identified. This is a large and impressive dataset: The authors have examined 701 rice accessions inoculated by 23 diverse *Xoo* strains. They identified 47 virulence-related genes and 318 rice *R*-genes Identified pairwise interactions between 125 rice candidate *R*-genes and 37 *Xoo* virulence genes.

This version of the manuscript is improved from an earlier version reviewed by this reviewer.

It is an interesting observation that most *R*-genes detected in *Xian* varieties are different from those in *Geng* varieties. This result supports the hypothesis that *Xian* and Geng subspecies became differentiated long before domestication and suggests a long history of coevolution of rice and *Xoo*.

Thanks for the positive comment.

Weaknesses:

Main conclusion is vague: "The observed complex genetic interaction systems between rice and *Xoo* are expected to exist in many other relationships between host plants and their pathogens." "Comprehensive analyses of this big data led us to discover complex genome-genome interactions between rice *R*- genes and virulence genes of *Xoo*, which shed important light on the coevolution of plants and their bacterial pathogen". It has previously been well established that different plant species share similar coevolutionary patterns and mechanistic relationships with their pathogens. It is hard to sort out the major advance of this paper.

A: We agree with the reviewer that different plant species are expected to share similar coevolutionary patterns and mechanistic relationships with their pathogens, which were obtained from cumulated knowledge from many previous studies. However, direct evidence and detailed descriptions of these coevolutionary patterns and mechanistic relationships between plants and their pathogens at the population genomic level remain lacking. In this regard, our study was the first one to show a comprehensive picture of corresponding high level diversity at large numbers of rice *R*-genes and virulence genes of *Xoo*, and their interactions at the population genomic level. This was the key contribution of this study to the current knowledge of the coevolution between plants and their pathogens. In addition, identification of large numbers of virulence related genes, including many new types such as T6SS effectors and TBDRs, etc., was also a major contribution to our current knowledge on the virulence related genes in pathogens.

Discussion of *R* genes is hard to follow. It seems that "candidate" *R* genes and known *R* genes are lumped together in the analysis. As far as I can tell, the authors did not discuss the structure of "*R* genes" until line 376 " large- effect *R*-genes for BB resistance are receptor-like kinases and those with NBS-ARC and LRR domains". I suggest they explain more clearly how they are defining and annotating *R* genes in this paper

A: Thanks for the comment. We have clearly defined the genes identified by GWAS in our paper into two types: resistance (*R*) genes and quantitative resistance (*QR*) genes. In the resubmit manuscript, *R* genes are noted as those detected known *R* genes or genes with receptor-like kinase domains or with NBS-ARC and LRR domains based on the MSU 7.0 version annotation of Nipponbare reference genome IRGSP 1.0, while the other detected genes were designated as *QR* genes.

They note that the *R* genes are clustered primarily in the ~3.1 Mb region of R30-R37 on rice chromosome 11. I suggest they discuss this result in light of what is already known about this region. For example, the *Xa21* locus and paralogs, map to chromosome 11.

A: Thanks for the suggestion. Combined with previous studies, the possible genetic mechanism of *R*-gene cluster was discussed in the resubmitted manuscript.

Were the authors able to expand on the observation that strains that evade *Xa21* have a mutation in *RaxX*? This would be a good "control" as the Xa21/RaxX system is well characterized.

A: Unfortunately, SNPs around *Xa21* were not identified as significant association signals with resistance to any of the four *Xoo* strains. This was most likely due to the fact that the resistant allele of *Xa21* originated from *Oryza longistaminata*, which would be either absent or very rare in our GWAS panel. The further genome-wide pairwise gene-gene interactions between rice and *Xoo* were carried out by the significant SNPs detected in rice GWAS panel. Thus, we cannot use the clearly revealed Xa21/RaxX system as the control. Nevertheless, the *Xa21/RaxX* system was briefly introduced and discussed in the resubmitted manuscript.

Is this accurate: "Fortunately, the fast evolution rate (one generation in ~20 minutes) of *Xoo*"? Please cite reference

A: Thank you. We have checked and corrected the *Xoo* cell cycle in the resubmitted manuscript.

The authors identify "47 virulence related genes" but none of the proposed phenotypes are validated through knockout of these genes in *Xoo* with subsequent infection analyses on rice

A: Thanks for the comment. According to permutation results, some of the detected 86 virulence genes are well-known virulence genes. So, we think that at least part of them must be true. We are validating some new genes, but we cannot show them in this paper. Here we want to focus on new methods for identification of virulence genes using a sample *Xoo* population.

The scoring system for "complete resistance" vs "partial resistance" appear to be subjective. The authors note that "lesion lengths were measured on 3 inoculated leaves per plant of 5 central plants of each rice accession plot in each replication 3 weeks after inoculation when lesions became stable (what does this mean?). The mean lesion length of the 30 plants of each rice accession from two experimental replications was used as the phenotyping data in further GWAS. " This sentence is hard to follow. They note they did two replicates for each inoculation, however the data is not shown. For a GWAS analysis to be accurate, 3 replicates would be preferable. Growth curve data for these analyses are needed for robust conclusions. Phenotype/inoculation data is missing.

A: As suggested, we have rewritten the part of "artificial inoculation" in the Methods section to make the experiment process more precise. In this study, a total of 20 leaves from ten biological replicates per rice accession of two sets of rice accessions were measured for each *Xoo* strain in two experimental replications.

We agree that the bacterial growth curve *in planta* can give a robust observation of whether the lesions became stable. However, the enormous amounts of collection of the bacterial growth curve data in planta for the two large-scale sets of rice accessions require an unimaginable amount of human work. In general, bacterial densities *in planta* can reach the growth platform stage 14 d after *Xoo* inoculation in rice plants (Pruitt et al., 2015; Luu et al., 2019). Here, we focus on the final level of bacterial blight resistance in rice germplasms rather than the exact time-point when the lesions became stable for each rice accession because the former has a more significant impact on yield loss in rice.

We provide the phenotyping data of the first set of 73 rice accessions in Supplemental Data Set 1 and phenotyping data of the second set of 701 rice accessions in Supplemental Data Set 4, respectively. According to the high heritability (74%-84%) of bacterial blight resistance in rice (Wang et al., 1994; Govintharaj et al., 2016) and the large scale of the artificial inoculation, we think two experimental

replications are sound for GWAS in this study. The sharp peak and strong association for bacterial blight resistance in rice based on the GWAS results (please see Figure 3) also support our view.

Rory N. Pruitt, Benjamin Schwessinger, Anna Joe, Nicholas Thomas, Furong Liu, Markus Albert, Michelle R. Robinson, Leanne Jade G. Chan, Dee Dee Luu, Huamin Chen, Ofir Bahar, Arsalan Daudi,David De Vleesschauwer, Daniel Caddell, Weiguo Zhang, Xiuxiang Zhao, Xiang Li, Joshua L. Heazlewood, Deling Ruan, Dipali Majumder, Mawsheng Chern, Hubert Kalbacher, Samriti Midha, Prabhu B. Patil, Ramesh V. Sonti, Christopher J. Petzold, Chang C. Liu, Jennifer S. Brodbelt, Georg Felix, Pamela C. Ronald. The rice immune receptor XA21 recognizes a tyrosine-sulfated protein from a Gram-negative bacterium. Sci. Adv., 2015, 1: e1500245.

Dee Dee Luua,b,1, Anna Joea,b,c,1, Yan Chend, Katarzyna Paryse, Ofir Bahara,b,2, Rory Pruitta,b,3, Leanne Jade G. Chand, Christopher J. Petzoldd, Kelsey Longa,b, Clifford Adamchaka,b, Valley Stewartf, Youssef Belkhadire, and Pamela C. Ronald. PNAS, 2019, 116(17): 8525-8534.

Wang Runhua, Lu Yonggen, Zhou Yanling, Cai Jianxi. Component analysis of genetic on resistance to bacterial blight in rice. Journal of South China Agricultural University, 1994, 15(1): 40-45.

Ponnaiah Govintharaj, Shalini Tannidi, Swaminathan Manonmani, Sabariappan Robin. Genetic parameters studies on bacterial blight resistance genes introgressed segregating population in rice. World Scientific News, 2016, 59: 85-96.

The authors note that *Xa40* is a dominant *R* gene that maps to an 80kb region. They note " Furthermore, evidence from partial correlations suggests that LOC_Os11g46890 is more likely to be the true *Xa40* than LOC_Os11g46900". A KO or complementation analysis is needed here to validate the hypothesis.

A: We performed the KO analysis, as suggested. Analysis of T1 plants containing homozygous mutations within *LOC_Os11g46890* indicates that resistance of the mutants to C5 is significantly lower compared to the wild type (Figures 4E and 4F), suggesting that *LOC_Os11g46890* is associated with BB resistance. We added these contents in the resubmitted manuscript.

Acronyms are unnecessary. CR= complete resistance.

A: As suggested, we corrected this. Thanks.

Titles of figure legends and grammar in legends are awkward and/or incorrect. Eg Supp Fig 12- "Determination of functional R gene for Xa40". What does this mean? There are other examples of challenging sentences scattered throughout the text.

A: Thanks a lot. We corrected the figure legends in the resubmitted manuscript.

It has previously been shown that recombination can easily generate 'new' *R*- genes. Previous research should be discussed. What is the specific advance of this paper? Please clarify

A: We highly appreciate this suggestion. We agree that recombination is a major driving factor for the generation of new *R*-genes, such as previous research on the evolution of *R*-genes such as *Xa21* gene family by Song et al. The possible genetic mechanism of generation of new *R*-genes in an important region for BB resistance in rice genome was discussed in the resubmitted manuscript.

Song WY, Pi LY, Wang GL, Gardner J, Holsten T, Ronald PC. Evolution of the rice Xa21 disease resistance gene family. Plant Cell, 1997, 9(8): 1279-1287.

TPC2020-LSB-00368 2nd Editorial decision – *declined* June 11, 2020

Thank you for choosing to send your manuscript entitled "The Complex Genetic Interaction Systems Leading to Balancing Selection Underlying the Arms-race of Rice and *Xanthomonas oryzae* pv. *oryzae* Revealed by Cross-species Two-dimensional GWAS" for consideration at The Plant Cell. Your submission has been evaluated by members of the editorial board as well as expert reviewers in your field, and we regret to inform you that we are not able to recommend publication of this manuscript. We have not made this decision lightly. We have had input from multiple scientists, and we have solicited post-review comments as well. Our present policy is to offer streamlined decisions and to not advise on the direction of the work by requesting extensive modifications or substantial additional experiments.

During the post-review consultation session, we also agreed that if you could address the major points raised by the reviewers by new experiments, we would welcome a resubmission. This would be treated as a new submission, but we would attempt to use at least some of the same reviewers. Nevertheless, reviewers will be

asked to assess as a new manuscript (i.e. are the claims fully supported by the data and do the results presented move the field forward?), and not only whether previous reviewer comments have been addressed.

What I envision is maybe another decline with possible revisions encouraged with a focus on the following.

While the editors and reviewers acknowledged that some of the concerns from the previous round had been addressed there were still significant unaddressed concerns from the previous round that we felt must be addressed prior to moving forward. The key points are listed below but please take all reviewers comments to heart and address them fully.

1) Describing how the 23 *XOO* isolates genomic variation is structured with regards to parameters central to GWAS like clonality, etc.

2) Describing and or visualizing how local LD structure in rice may be affecting the epistasis work.

3) Better description of the rice validation efforts.

4) Remove the unsupported evolutionary arguments.

5) Report heritability of all assays so as to provide evidence on reproducibility.

It will be important to convince the editors and reviewers that the study adds significant new understanding(s) of mechanisms or processes, or otherwise represents a significant advance in the field, and that the major claims made are fully justified by the data presented. This includes careful consideration and explanation of the various controls used in experiments, the extent and manner of replication, and the statistical analyses used. Sampling methods and the nature of "biological replicates" should be described precisely (i.e. different plants, parts of plants, pooled tissue, independent pools of tissue, sampled at different times, etc.), along with a clear description of and rationale for any statistical analyses conducted. The reader should know exactly what was sampled; what forms the basis of the calculation of any means and other statistical variables and parameters reported. This is also necessary to ensure that proper statistical analysis was conducted.

--- Reviewer comments:

[Reviewer comments shown below along with author responses]

TPC2020-LSB-01058 Submission received Dec. 17, 2020

Reviewer comments on previously declined manuscript and **author responses:**

Reviewer #1:

1) Describing how the 23 *XOO* isolates genomic variation is structured with regards to parameters central to GWAS like clonality, etc.

Response: Thank you for the comment. Following the Editor's suggestion, we have added more corresponding results and discussions on the *Xoo* population structure in the resubmitted manuscript (see lines 141-166, lines 494-502, and Supplementary Figure 1). We understand that with limited recombination within the bacterial genomes, the LD decay in the clonal population structure is expected to be slow, forming large haplotype blocks, making the causal SNPs indistinguishable from linked SNPs. Nevertheless, the phylogenetic tree based on SNPs allowed the detailed identification of genetic relationships among the *Xoo* strains, not only at the level of population clusters, but also at the resolution of subpopulations and individual relationships. In the previous version, we showed the phylogenetic tree based on 33,006 SNPs stratified *Xoo* strains into clear groups with strong geographic origins (Figure 1B). Furthermore, by running the software HREfinder (Wang et al. 2013) with the information of SNP sites as input, we were able to characterize the recombination events during the evolution and divergence of the *Xoo* strains. Here, we observed a ~2.5 kb genome-wide LD decay (dropped to half of its maximum value) distance in the *Xoo* genomes with a single recombination peak on the *Xoo* genome among different *Xoo* strains (Supplemental Figure 1E and 1F). This level of LD decay was similar to that of some phytopathogenic fungi (i.e., *Fusarium graminearum* with mean LD ∼1 kb and *Parastagonospora nodorum* with mean LD∼5–10 kb) (Gao et al., 2016; Talas et al., 2016). The rapid LD decay in the *Xoo* population indicated a high recombination rate and diversity in the sampled *Xoo* population, and therefore a reasonably overall high resolution in mapping pathogenic determinants in this study.

Pan-genome analysis of the 23 *Xoo* genomes revealed significant levels of gene presence/absence variation (Supplemental Figure 1A) with a similar population structure to that inferred from SNPs (Supplemental

Figures 1B and 1C). Moreover, the 23 *Xoo* strains were classified into 4 virulence groups (Figure 1A) based on the lesion lengths of 73 diverse rice accessions (Supplemental Data Sets 1 and 2). In this study, we used both linear model and linear mixed model (LMM), taking advantage of multiple phenotypes (lesion length caused by different *Xoo* strains in 73 rice accessions) to identify the virulence-related loci of *Xoo*. GWAS based on LMM were carried out with the first two principal components used as covariates to control the problem from limiting population stratification. Our previous genotype permutation analysis and specificity analysis with literature search suggested that the probability of observing ≥86 (the number we detected here) significant SNPs in a random CAS-GWAS is less than 0.0001. The false discovery rate is estimated to be 0.17, which means 71 out of the 86 are true signals. We think the false discovery rate is reasonable here and our GWAS approach can control close relatedness within samples by capturing the fine structure of populations faithfully.

2) Describing and or visualizing how local LD structure in rice may be affecting the epistasis work.

Response: Thank you for the comment. According to reviewer #1's suggestion, we have now adopted the method of using an LD threshold to limit the number of SNP pairs in the same rice LD blocks in the rice-*Xoo* interaction analysis. Specifically, we constructed local LD structures around 5,432 significant SNPs in rice GWAS panel and only those SNPs with the most significant hits and/or with the largest R^2 (the percent phenotypic variation explained) within each LD block were selected for further interaction analysis. As a result, a total of 172 index SNPs within the 75 rice LD blocks were selected for determining significant rice-*Xoo* interactions. Finally, the significant interactions between all pairs of SNPs were binned into gene-to-block interactions between *Xoo* and rice. For example, when multiple significantly interacted SNPs were located in the same *Xoo* gene and the same rice LD block, respectively, only the strongest SNP-SNP interaction (with minimum *p*-value) was retained as the gene-to-block interaction. We rewrote the methods and results of the rice-*Xoo* interaction analysis in the resubmitted manuscript (see lines 371-423, lines 894-916, Figure 7 and Supplemental Data Sets 6-8).

3) Provide a better description of the rice validation efforts.

Response: We have added a more detailed description of the functional validation for *LOC_Os11g46890* to Methods ("Vector construction and rice transformation", see lines 852-874) and Results (see lines 305-317). Thank you for the felicitous comment.

4) Remove the unsupported evolutionary arguments.

Response: The unsupported evolutionary arguments have now been removed in the resubmitted manuscript. Thank you.

5) Report heritability of all assays so as to provide evidence on reproducibility.

Response: We have supplemented the heritability estimation of lesion length caused by each *Xoo* strain (see lines 136-138), and provided the raw phenotypic data in Supplemental Data Sets 1 and 4 of the resubmitted manuscript.

Reviewer #1:

I appreciate the effort the authors made in addressing my previous concerns and those of the other reviewers.

I think the elaborations of the Xoo GWAS methods was helpful to better understand the method, and the use of genotype permutations and specificity analysis with literature search shows that it probably is successfully prioritizing important variants. But is the following sentence correct: "23 candidate disease gene detected with a specificity of 0.49), defined as the average value of sensitivity and specificity at the genic level"? I don't think the average of sensitivity and specificity is also called "specificity"

Response: We thank the reviewer for pointing out this misleading statement and rewrote the sentence as "we then assessed the performance of combinations of the *p*-value and CAS score cutoffs by assuming that all the candidate pathogen genes are true positives, the combination of the *p*-value cut-off at 0.001 and CAS cutoff at 0.3 gives the highest overall accuracy (the average value of sensitivity and specificity at the genic level)." (see lines 799-800).

For the Rice GWAS, it's somewhat "ad-hoc" to combine Bonferroni thresholds and permutations. The point of the permutations is that you can use the data to derive a more powerful threshold to use with a false discovery rate (control over the number of false positives) than you can with a Bonferroni threshold which controls the family wise error rate (control over the chance of a single false positive). Usually a study will use one or the other, justified based on the balance between false negatives and false positives. If you're comfortable with a

false-discovery-rate control (which is very reasonable), then I'd recommend just using the permutation-based FDR=0.05 threshold itself. This is an optional change - your methods are clear and others can make their own conclusions. But I think you're throwing away real results.

Response: Thank you very much for the kind suggestion, which much improved our understanding of the threshold selected in GWAS. In our previous response, we mentioned that the Bonferroni thresholds used in this study were less than or equal to 0.0179 of FDR based on permutations. Thus, the results with these two thresholds were very similar. We agree that some weak association signals (hits) were dropped by a Bonferroni threshold compared with using a permutation-based threshold (FDR=0.05) in the rice GWAS, which would lead to some false negative signals. However, we think that most of the false positives should locate in the rice genomic regions/LD blocks detected in our previous manuscript and would not change the main message and results of the study. In the resubmitted manuscript, we removed the descriptions about permutations and retained the slightly more stringent Bonferroni threshold. Moreover, to solve the problem of false negative caused by using the more stringent Bonferroni threshold, we use the effective number of independent SNPs (N) estimated by GEC software (Li et al., 2012b) to calculate significant thresholds (0.05/N) in each rice GWAS panel rather than using all SNPs (see lines 836-838). We sincerely hope that the reviewer would agree with us.

For the interaction analysis, I am still uncomfortable with the thresholding method applied. You're choosing an extremely conservative p-value threshold of ~5e-13 to declare something significant. The authors defend this choice as by the number of potential interactions between all pairs of SNPs. But you didn't (and weren't going to) test all pairs of SNPs. It seems the reason to use this very low threshold is to limit the number of hits (to already a very large number). The problem with this is that with such a stringent threshold, you'll miss a lot of important interactions, and once a p-value is low enough, lower and lower values mean "more evidence that the effect is real", but not more evidence that the effect is "important". The most significant snp-pairs may be those with small effect sizes, while slightly less significant ones may have larger effect sizes. A better way to limit the number of hits would be to i) use a second threshold on the magnitude of the interactions (e.x. Li et al Genetics 2020), or ii) use an LD threshold to limit the number of SNP pairs on the same haplotype. I think that the second option might be particularly successful. Inspecting Figure 7, it appears that a very large proportion of the highly significant interactions involve a region on rice chromosome 11. I inspected the SNP data in the "49rice-sub-geno.csv" supplemental data file, and it appears that SNPs across this whole region (including a large number of genes) are essentially in perfect LD. One way to see this would be to arrange the genes in Figure 7B according to genomic location rather than correlation (say connecting the genes to the chromosome maps above in 7A) to show that all genes in this block have the same interaction patterns with *Xoo* genes. Unfortunately, this would mean that it is not really possible to identify which genes within this LD block cause the inter-species interaction at this locus. But filtering the hit-list for only a single SNP on this haplotype would greatly reduce the number of significant hits, allowing you to use less stringent overall threshold and perhaps detect more interactions genome-wide.

Response: We completely agree with your constructive comments. In the resubmitted MS, we adopted the method of limiting the number of hits by an LD threshold to reduce the number of SNP pairs on the same rice block, as suggested by the reviewer. Local LD structures around all significant SNPs detected in rice GWAS panel were constructed and only the SNPs with the most significant hits and/or with the largest R^2 (the percent phenotypic variation explained) within each LD block was retained for further interaction analysis. Specifically, a local LD based approach was used to identify independent significant association peaks in the rice GWAS panels using PLINK' command line: 'plink --clump --clump-p1 6.81e-8 --clump-p2 1e-5 --clump-r2 0.5 --clump-kb 300'. We further selected the peaks containing ≥1 significant SNP at the significant level of 6.81×10^{-8} and ≥1 extra SNP at the significant level of 1.0×10^{-5} and in pairwise LD (r^2>0.5) with the significant SNP in the same LD block. Then, to select the most likely independent representative SNPs (referred to as index SNPs), we estimated the effects of SNPs by R^2 using 'lm' function in R and selected the SNPs with the most significant *p*-values for each *Xoo* strain and/or with the largest R^2 across the peaks within each LD block. As a result, a total of 172 index SNPs within the 75 rice LD blocks were identified for the rice-*Xoo* interaction analysis. Then, using the Bonferroni correction for multiple testing with the number of tests we conducted here, the *p*-value threshold for significant interaction was 1.52×10^{-7} [0.05/(1,909 *Xoo* variants in virulence-related genes × 172 rice index SNPs)]. Finally, the significant interactions between all pairs of SNPs were binned into gene-to-block interactions between *Xoo* and rice. For example, when multiple significantly interacted SNPs were located in the same *Xoo* gene and the same rice LD block, respectively, only the strongest SNP-SNP interaction (with minimum *p*-value) was retained as the gene-to-block interaction. Here, we identified 51 *Xoo* genes that significantly interacted with 35 rice LD blocks distributed on 12 rice chromosomes. The results indicate that more genome-wide interactions could be identified by limiting the number of SNP pairs on the same LD block than previous results. We rewrote the methods and results of the

rice-*Xoo* interaction analysis in the resubmitted manuscript (see lines 368-423, lines 894-916, Figure 7 and Supplemental Data Sets 6-8). Thank you.

Minor issues: 842 - raw p-values were "multiplied" by the # tests, not divided

Response: Thank you for pointing out this mistake, and we have corrected it in the resubmitted manuscript (see Supplemental Data Set 7).

843 - these are multiple testing corrected p-values. q-values have a specific meaning relative to positive false discovery rates

Response: Thank you for pointing out this mistake, and we used raw *p*-values in the resubmitted manuscript and set the *p*-value threshold for significant interaction at 1.52×10^{-7} based on Bonferroni correction [0.05/(1,909 *Xoo* variants in virulence-related genes × 172 rice index SNPs)].

Reviewer #2:

1.) Following my criticism of using the term "co-evolution", the authors have replaced this with "arms race" although the term remains inexplicably in the abstract. The problem is that the concept of arms race applies only to co-evolutionary interactions between species and is therefore inappropriate as well. Please use simply the term "(host) adaptation" to describe the process of *Xoo* adapting to rice as a host. It may well be that ancestors of rice were in arms races with ancestors of *Xoo* but this needs to be demonstrated first.

Response: Thank you for your suggestions. We completely agree with your comments. We did all the related changes in the entire manuscript as the reviewer advised.

2.) Citing the literature of about plant pathogens and GWAS applications has been achieved currently by citing a review from 2017 in Frontiers saying that there has been hardly any GWAS. Simple literature searches of the following pathogen species Botrytis, Zymoseptoria, Parastagonospora, Magnaporthe and Pseudomonas with the keyword GWAS will identify the relevant literature. The manuscript still treats GWAS on pathogens as an innovation. My request remains that key advances or limitations of applying GWAS in pathogens is briefly cited and discussed. Problems as encountered here with genetic structure have shown up in the literature already and apply clearly to this study here as well.

Response: Thanks very much for your constructive suggestions and comments. We updated the key advances of GWAS on pathogens in the Introduction section "Application of GWAS to identify genomic regions associated with pathogenicity have been reported in bacterial and fungal pathogens (Bartoli and Roux, 2017; Sánchez-Vallet et al., 2018), including *Pseudomonas syringae* (Monteil et al., 2016), *Parastagonospora nodorum* (Gao et al., 2016), *Fusarium graminearum* (Talas et al., 2016) and *Zymoseptoria tritici* (Hartmann et al., 2017)." (see lines 68-72)

We agree that there are specific challenges when identifying genomewide associations in bacteria primarily comprising highly structured populations due to clonality. The clonal population structure with limited recombination is expected to form very large haplotype blocks, making the causal SNPs indistinguishable from other linked SNPs (Falush and Bowden, 2006). To overcome this problem, we carefully selected the *Xoo* population consisted of 23 strains representing 18 different *Xoo* races in the tropics and 5 virulence-differentiated groups of the Chinese *Xoo* population based on previous results of race grouping (Vera Cruz et al., 2000; Zhou et al., 2011; Quibod et al., 2016). Thus, causal variations related to virulence differentiation in the *Xoo* populations from the Philippines (tropics) and China are more likely to be detected. Also, we observed a recombination hot spot and a rapid LD decay (r^2 dropped to half of its maximum value at ∼2.5 kb) in the sampled *Xoo* population. Higher recombination rates are expected to cause faster LD decay and ultimately result in higher mapping resolution. Similar results were reported in some phytopathogenic fungi (i.e., *Fusarium graminearum* with LD decay of ∼1 kb and *Parastagonospora nodorum* with LD decay of ∼5–10 kb) (Gao et al., 2016; Talas et al., 2016). To better control the problem of population stratification and small sample size on the power of detecting causal mutations related to virulence of *Xoo*, we took advantage of multiple phenotypes (mean lesion length caused by each *Xoo* strain in 73 rice accessions) in our GWAS and using LMM with the first two principal components used as covariates, which gave a reasonable false discovery rate (0.17) of our GWAS based on permutation analysis. In other words, our GWAS analyses were based on 23x73=1,769 phenotypic observations (mean lesion lengths) for each SNP in detecting single virulence loci in the *Xoo* genome or on 23x49=1,127 for each rice-*Xoo* interacting SNP pair in detecting interacting R-gene block/virulence gene pairs. Because the LD decay is ~300 kb in the rice GWAS panel, we adopted the LD haplotype blocks each containing multiple significant SNPs as the input rice genotypic data to control the number of significant interactions detected in the resubmitted MS (as suggested by Reviewer

#1). Indeed, our results that most of the detected associations were well-known virulence genes suggested that the power and mapping resolution of GWAS in *Xoo* of this study were not obviously limited by intrinsic clonal population structure. A brief discussion on this point has been added to the Discussion section (see lines 492-502, lines 538-549 and lines 590-594).

3.) Previous comment: "Association mapping performed with only 23 *Xoo* strains is extremely low. At this population size, chance associations largely overwhelm true signals of phenotype-genotype associations. The low sample size is compounded by genetic substructure among the 23 strains and likely clonal reproduction. Unless the authors can provide a detailed rationale why it would be appropriate to perform association mapping with this population, this section of the manuscript may have to be omitted. Important factors to investigate and justify include levels of recombination, patterns of LD, the choice of proper regression models, etc. Please see some excellent reviews by J. Bergelson, D. Weigel or M. Nordborg on these issues and revise accordingly."

The authors respond with mentioning the permutation approach, which is surely a step in the right direction. However, there is a troubling assessment stating that "Thus, false negatives, instead of false positives, would be a more serious problem in this study". I am not sure to follow what the argument here is. Reporting false positives seems a more egregious problem as it introduces false associations into the literature. Failure to report an association (false negative) due to the lack of power in a study using 23 pathogen strains seems a more benign issue.

Second, my request to properly characterize the pathogen population in terms of LD decay, recombination rates and clonality have been ignored. This is essential for a reader to judge the validity of the pathogen GWAS panel (especially if n = 23).

Response: Thanks for your comment and we apologize for the unclear explanation on the reviewer's concern regarding the possible high false positive rate of the detected associations from the small population size of *Xoo* without appropriate characterization of the effect of clonality and LD decay in the *Xoo* population. In this study, the *Xoo* population consisted of 23 strains representing the 18 different *Xoo* races in the tropics and 5 virulence-differentiated *Xoo* groups in China based on extensive race grouping in the past (Vera Cruz et al., 2000; Zhou et al., 2011; Quibod et al., 2016). To estimate clonality of the *Xoo* population, multi-locus genotypes (MLGs) were assigned by the 'poppr' package in R. Consequently, a relatively high number of MLGs were found in the sampled *Xoo* population (22 MLGs out of 23 strains), suggesting that the clonality of the *Xoo* population was low (see lines 143-145). According to the reviewer's suggestion and using the software HREfinder (Wang et al. 2013) and the information of SNP sites as input data, we were able to characterize the recombination events during the evolution and divergence of the *Xoo* strains. We found recombination occurred in a non-uniform manner among the *Xoo* strains, with a peak recombination hotspot in the region of 1.57 Mb - 1.86 Mb in the *Xoo* genome where genes related to chemotaxis and two-component system are highly enriched (see lines 154-161 and Supplementary Figure 1E). Notably, recombination occurred more frequently (~900 recombination events) among SV or MV strains, P6 ($PXO99^A$), P6d, P5, P7, P8 and P3b from the tropics. In contrast, recombination occurred less frequently in the genomes of WV Chinese strains, C3, C4, C2, C1, C7 and C6. These facts suggested that elevated recombination may have directly contributed to enhanced virulence of *Xoo* during the rice-*Xoo* coadaptation in the tropics. Because higher recombination rates tend to cause faster linkage disequilibrium (LD) decay which ultimately results in higher mapping resolution. Here, the genome-wide LD decay (r^2 dropped to half of its maximum value) distance in the *Xoo* population was estimated to be ~2.5 kb (covering ~2-3 genes). Thus, this level of LD plus the presence of 33,006 SNPs among the *Xoo* genomes indicated the high recombination and diversity in the *Xoo* population suitable for GWAS. We have supplemented these results in the resubmitted manuscript (see lines 143-145, lines 154-166, and Supplemental Figure 1E and 1F).

Given the low effects of clonality and LD decay in the *Xoo* population, we believe that the false positive rate (the reviewer's concern) in the detected associations in the *Xoo* population should be very low based on the following three arguments. First, the traits of primary interest, such as virulence and host-association, have evolved recently and under strong positive selection on the bacterial populations. When these bacterial traits are controlled by mutations of large-effect, causal mutations should be detectable even with relatively small samples of bacterial (Farhat et al. 2014). Statistically, Wang and Xu (2019) demonstrated that the significance test of GWAS is the product of the sample size and the magnitudes of QTL effects. Thus, the sample size has already been taken into account in the GWAS analysis when the test statistic is calculated. Thus, if the test statistic of a SNP was highly significant in our GWAS analyses, the QTL effect must be very large to compensate for the small sample size to be detectable (Wang and Xu, 2019). Secondly, we agree with the reviewer that false positives are a more egregious problem in the literature. As mentioned in our response to reviewer #1, to overcome the small sample size and take advantage of multiple phenotypes (mean lesion

length caused by each *Xoo* strain in 73 rice accessions), we utilized a combined association score (CAS) that combined independent association results to improve the possibility of identifying true signals (see lines 771-807 of Methods for details). The number of events here is 23*73=1,679, which is even higher than most single-phenotype GWAS experiments of plants (N plants * 1 = N events). In other words, the actual population size for detecting virulence loci in our GWAS analyses was based on 23 x 73 = 1,679 phenotypic observations (mean lesion lengths) for each SNP in detecting single virulence loci in the *Xoo* genome. Our previous genotype permutation analysis suggested that the probability of observing ≥86 (the number we detected here) significant SNPs in a random CAS-GWAS is less than 0.0001. The false discovery rate was estimated to be 0.17 by permutation analysis, which means at least 71 out of the 86 are true signals. This is supported by our result that most of the detected associations were indeed well-known virulence-related genes, suggesting that the power and mapping resolution of our GWAS were not obviously limited by intrinsic clonal population structure and by the population size. Thirdly, as reviewer #1 indicated, large numbers of the detected interactions could result from the redundancy of linked SNPs because of the low LD decay in the host population and small sample size of the pathogen population. We reorganized the detected significant SNPs into LD blocks based on local LD structure in the host population and integrated additional *Xoo* SNPs (both SNPs in known virulence genes and those in newly detected genes) in our interaction analyses, The results have significantly reduced the number of significant SNP-SNP interactions into more meaningful gene-for-gene interactions, though primarily in a multiple-for-multiple manner. Based on the same argument, we believe that the false negative rate of being able to detect important causal mutations related to virulence in this study should be low. We have added the relative discussion on this issue to the resubmitted manuscript (see lines 490-502, lines 538-549 and lines 590-594).

References:

Wang Meiyue and Xu Shizhong. Statistical power in genome-wide association studies and quantitative trait locus mapping. Heredity, 2019, 123(3): 287–306.

Farhat MR, Shapiro BJ, Sheppard SK, Colijn C, Murray M: A phylogeny-based sampling strategy and power calculator informs genome-wide associations study design for microbial pathogens. Genome Med 2014, 6:101.

4.) The term balancing selection is used in the title but there's no test in the manuscript showing that any locus is indeed under balancing selection. I find one instance where high diversity at a locus is interpreted as being due to balancing selection. This may well be true but there is no statistical assessment. The term balancing selection should therefore be removed from the title.

Response: We thank the reviewer for highlighting the evolutionary arguments about balancing selection in our previous manuscript and apologize that we could not fully support our point at this stage. The term balancing selection has now been removed from the Title and Discussion sections of the resubmitted manuscript.

<u>Reviewer #3:</u>

This reviewer appreciates the huge amount of data collection. In this revised version, the authors have addressed the reviewers' comments, but unfortunately, not always as clearly as one would hope.

Response: Thank you very much for your positive and kind comments on our manuscript. We hope that with the resubmitted manuscript we could address the following concerns and satisfy the requests of reviewer #3.

For example, some of the sentences remain hard to follow. Eg. "our study was the first one to show a comprehensive picture of corresponding high level diversity at large numbers of rice *R*-genes and virulence genes of *Xoo*, and their interactions at the population genomic level. ".

It is unclear what the authors mean by "high level diversity". Instead of "large numbers of rice *R*-genes and virulence genes", can the authors provide precise numbers?

Response: We are sorry that we didn't describe "high level diversity" clear and provide precise numbers of the relevant genes before. For *Xoo*, "high level diversity" means a much higher SNP density of a virulence-related *Xoo* gene compared to the background genes. For rice, it means the high nucleotide diversity and numbers of SNPs, CNVs, and gene presence/absence variations of the genomic regions harboring detected R genes. Specifically, we discovered 23 (~49%) of 47 virulence-related genes with significantly higher SNP densities than other genomewide background genes in *Xoo* (see Supplemental Table 2). For rice, we found 261 (~82%) of 318 genes associated with resistance to *Xoo* (55% of the significant SNPs and 44% of non-transposon genes) located in the ~3.3 Mb region of R30-R37 on chromosome 11 in rice. Compared with the landraces, the modern varieties displayed increased diversity in regions of R30-R37 (Figures 6C and 6D).

Using the RPAN database (Sun et al., 2017), we found that 67.5% of the genes detected in the R30-R37 regions were distributed/dispensable. These identified genes showed 2.2-fold enrichment in comparison to the genome background with a distributed gene rate of 0.30 (the hypergeometric test, *p* =1.7e-20). In particular, genes in R30-R37 tended to have significantly higher gene copy variations when the *Xian* Minghui63/Zhenshan97 genomes (Zhang et al., 2016) were compared with the *Geng* Nippobare genome (Supplemental Figure 11D). An additional peak of large deletions (>100bp) was detected in R30-R37 among the 701 genomes (Figure 5C), suggesting high frequencies of homologous recombination in this region. All these results indicate that R-genes in this region evolved rapidly, resulting primarily from unique nature of this region harboring high numbers of SNPs, CNVs, and gene presence/absence variations. As suggested by the reviewer, we have now added these results and have more clearly described what was the meaning of "high level diversity" in the resubmitted manuscript (see lines 200-201, lines 345-366, lines 464-472 and lines 502-504).

We have provided precise numbers instead of "large numbers" and rewrote the relevant sentence in the resubmitted manuscript, which now reads as follows: "At the genomic level, we observed a high level of diversity at 3,735 (68.8%) of 5,432 significant SNPs across the rice genome at which significant frequency shifts for resistance alleles occurred during modern breeding, which was accompanied by a very high level of diversity at many virulence-loci (23 of 47 virulence-related genes with significantly higher SNP densities than other genomewide background genes) in the *Xoo* populations." (see lines 345-347 and lines 444-448).

"This was the key contribution of this study to the current knowledge of the coevolution between plants and their pathogens. In addition, identification of large numbers of virulence related genes, including many new types such as T6SS effectors and TBDRs, etc., was also a major contribution to our current knowledge on the virulence related genes in pathogens."

As the authors acknowledge, they have not been able to confirm if these "new types" of genes serve as virulence factors and do not enumerate how many have been confirmed. They indicate that they identify "47 virulence related genes" but none of the proposed phenotypes are validated. In their response the authors note that "some of the detected 86 virulence genes are well-known virulence genes. So, we think that at least part of them must be true. We are validating some new genes, but we cannot show them in this paper." This is a confusing statement. I suggest that if they wish to only focus on new methods for identification of virulence genes then they make this clear. Also the methods need to be validated experimentally for at least some subset of these "new types of virulence" genes.

Response: Thanks to the Reviewer's good comments. We fully agree with the reviewer and include the statement "we discovered three new groups of virulence-related genes", including T6SS effectors, TBDRs and three novel genes involved in energy metabolomics (see lines 507-532), according to the reviewer's suggestion. We also removed the confusing statement, as validation of the new virulence genes takes much a long time. As indicated in the resubmitted manuscript, the primary discovery of this study was the demonstration to apply the one-/two- dimensional GWAS for high efficient discovery of large numbers of rice R-genes and *Xoo* virulence genes, and complex interactions between rice R-genes and *Xoo* virulence genes using large-scale genome sequencing and phenotyping data. These discoveries were based on strong genetic and statistical evidence. Thus, our results will greatly facilitate future efforts to experimentally validate the new/novel R-genes/virulence genes, their interactions at the molecular level, even though these validation experiments remain to be done in future.

It is still somewhat difficult to determine the quality and reproducibility of the inoculation data. For example, Supplemental Data Set 1 shows only a single data point for each inoculation. Supplemental Data Set 4 shows only 2 data points for each strain. Yet in the methods the authors say "In this study, a total of 20 leaves from ten biological replicates per rice accession of two sets of rice accessions were measured for each Xoo strain in two experimental replications." Please include the entire dataset (or if it was shown in the paper and this reviewer overlooked it, please make the location clear so the reader can find the data). Please also write a more detailed legend for the supplementary files. I agree that the enormous amounts of collection of the bacterial growth curve data in planta for the two large-scale sets of rice accessions require too much work to be feasible.

Response: Thank you for your suggestion and understanding of our large-scale phenotypic evaluation and sorry for the inconvenience. The single point data for each inoculation in previous Supplemental Data Set 1 was the input data for our *Xoo* GWAS and two-dimensional GWAS analyses, which was the average lesion length calculated from 2x5x2=20 measurements (2 leaves on each of the 5 plants in each of the 2 replications for each rice accession inoculated by a single *Xoo* strain). Similarly, the single point data for each inoculation in previous Supplemental Data Set 4 was the input data for our rice GWAS analyses, which was the average lesion length calculated from 3x3x2=18 measurements (3 leaves on each of the 3 plants in each of the 2

replications for each rice accession inoculated by a single *Xoo* strain). Now in the resubmitted manuscript, we attached all raw lesion measurement data from which the input data sets for one- and two-dimensional GWAS were performed (see Supplemental Data Sets 1 and 4). According to the editor's comment, we reported the heritability of all assays as the evidence on reproducibility in Supplemental Data Set 1 and Result section in the resubmitted manuscript (see lines 136-138). Of the 23 *Xoo* strains, the estimated broad-sense heritability of lesion lengths caused by different strains were all very high, ranging from 0.870 for P6 to 0.979 for P9a. Following the reviewer's suggestion, we have added more detailed descriptions to the legends of these supplementary files.

The authors note that *Xa40* is a dominant *R* gene that maps to a 80kb region. To determine if *Xa40* (LOC_Os11g46890 ?) is the causative *R* gene, the authors made three CRISPR lines: Two at "target site 1" and 1 at "target site 2". They analyzed T1 plants containing homozygous mutations within LOC_Os11g46890. They found that the three mutants are more compared with the wild type parent (Figures 4E and 4F), suggesting that LOC_Os11g46890 is associated with BB resistance.

This data suggests that LOC_Os11g46890 is a resistance locus and is quite interesting. However, additional details (the authors likely already have this data) are needed so that readers can fully understand this study. What is the effect of these mutation on the predicted ORF? Did they observe other mutations in these lines? How did they confirm homozygosity of the mutations in the inoculated lines? How were the CRISPR plants made? Where are the guide RNA constructs described and what methods were used to create the plants? More details are needed so the readers can understand how this study was done and to follow the logic of the authors.

Response: Thank you very much for this valuable comment and sorry for the lack of detailed description in the "Vector construction and rice transformation" of the Method section. We have included more experimental details and results mentioned by the reviewer in the Method and Result sections of the resubmitted manuscript (see lines 305-317 and lines 850-874).

As shown in Figure 4E&F, three independent homozygous T_1 mutants with different types of mutation in the coding region were obtained through the CRISPR/Cas9 multiplex gene editing method. Among them, line JK143 (1 bp insertion on target site 1) caused a stop codon and produced a truncated protein, while the other two lines caused frame shift and encoded putative new proteins (Supplemental Figure 8). Apart from the three mutants shown in Figure 4F, we didn't observe other mutations in transgenic lines.

To confirm mutations, we designed primer LOC_Os11g46890-TF and LOC_Os11g46890-TR to amplify the fragment spanning the two target sites in T0 transgenic lines. Through Sanger sequencing with gene-specific sequencing primer LOC_Os11g46890-SP, homozygosity mutants in target sites were identified.

CRISPR plants were made by these four major steps: 1) to design appropriate target sites according to CRISPR vector; 2) to construct CRISPR vector; 3) to transfer the CRISPR vector into rice callus through agrobacterium-mediated transformation method; 4) to detect the transgenic lines and discover mutants.

The guide RNA constructs were a part of CRISPR vector construction as the previously described multiplex editing method in the reference (Ma et al., 2015). In this study, two guide RNAs were designed to target the exon of LOC_Os11g46890 using the web-based software CRISPR-GE (http://skl.scau.edu.cn/). To construct guide RNA constructs for target site 1, the first PCR was carried out in two separated reactions with U-F/U6a-LOC_Os11g46890 and gRT1-LOC_Os11g46890/gR-R using pYLsgRNA-OsU6a as the template. Then, the second PCR was performed to generate a guide RNA construct containing target site 1 by overlapping PCR with the first PCR product. Likewise, to construct the guide RNA constructs for target site 2, the first PCR was carried out in two separated reactions with U-F/U6b-LOC_Os11g46890 and gRT2-LOC_Os11g46890/gR-R using pYLsgRNA-OsU6b as the template. And then the second PCR was performed to generate a guide RNA construct containing target site 2 by overlapping PCR with the first PCR products. Finally, these two guide RNA constructs were inserted into the pYLCRISPR/Cas9Pubi-H vector through a restriction-ligation reaction. The transgenic plants were created by the agrobacterium-mediated transformation method (Hiei et al., 1994). All the above-mentioned primers were provided in Supplemental Table 6.

This paper includes a lot of interesting data but unfortunately it is quite difficult to find the "gems" (most interesting results), as it is now written.

Response: We agree with the reviewer that the key discoveries from this study were poorly summarized. After carefully answer the reviewers' comments, we have completely rewritten Discussion (see lines 488-504, lines 587-598 and lines 618-647) and abstract, which better summarized the key discoveries and "gems" from this study.

TPC2020-LSB-01058 1st Editorial decision – *revision requested* Feb. 15, 2021

We have received reviews of your manuscript entitled "The Complex Genetic Interaction Systems Leading to Reciprocal Adaptation of Rice and *Xanthomonas oryzae* pv. *oryzae* Revealed by Cross-species Two-dimensional GWAS." Thank you for submitting your best work to The Plant Cell. The editorial board agrees that the work you describe is substantive, falls within the scope of the journal, and may become acceptable for publication, pending revision and potential re-review. We ask you to pay attention to the following points in preparing your revision:

In addition to the reviewers more specific requests, it is essential to focus on the list of following requests from the reviewers.

Report LD Decay with and without the region of high recombination to allow the reader to fully interpret the results. This will help to understand how the GWA may be influenced by the clustering of recombination.

Given that epistasis is not explicitly supported in the manuscript, it is necessary to alter the discussion on epistasis given the lack of statistical or numerical support. At the present it is a possibility and needs to be presented as simply a possibility. The current text makes epistasis appear to be a supported fact which is not presently the case.

To help place the results in context, it was requested to discuss the limitations of two replicates and how G x E can influence the results.

Please, clarify N in all datasets.

It was noted that the TE statements do not seem supported by data in the manuscript and should be removed.

Finally, please work to connect to the existing literature on race structure and causality better to incorporate this work into the existing data.

--- Reviewer comments:

[Provided below along with author responses]

TPC2020-LSB-01058R1 1st Revision received April 6, 2021

Reviewer comments on previous submission and **author responses**:

<u>Comments from the Editor:</u>

We ask you to pay attention to the following points in preparing your revision:

In addition to the reviewers more specific requests, it is essential to focus on the list of following requests from the reviewers.

Report LD Decay with and without the region of high recombination to allow the reader to fully interpret the results. This will help to understand how the GWA may be influenced by the clustering of recombination.

> **Response: Thanks for the kind comment and we agree with reviewer #2 that the level of recombination has been an influencing factor of GWA. According to the editor's comment, we performed a comparison between the LD decay curves across the *Xoo* genomes with and without the recombination hotspot (Supplemental Figure 1B). As shown in Supplemental Figure 1B, similar LD decay curves were observed, but with the recombination hotspot (1.57-1.86 Mb), the observed average LD block (r^2 decays to 0.25) was ~2.1 kb, slightly smaller than the average LD (~2.4 kb) without the recombination hotspot. This small difference in the average LD block sizes within and outside the recombination hotspot had little impact on the number and resolution of the detected virulence-related genes in both cases (Fisher's exact test, *P* = 0.119 [the recombination hotspot vs. the rest of the genome]), though we indeed observed significantly more virulence-related SNPs (i.e. a higher power of association detection) in the recombination hotspot (Fisher's exact test, *P* value = 0.009 [the recombination hotspot vs. the rest of the genome]). We have included the relevant description in the revised manuscript (see lines 166-171 and 186-191).**

Given that epistasis is not explicitly supported in the manuscript, it is necessary to alter the discussion on epistasis given the lack of statistical or numerical support. At the present it is a possibility and needs to be presented as simply a possibility. The current text makes epistasis appear to be a supported fact which is not presently the case.

Response: We agree with the editor and reviewer #4 that the evidence for epistasis among different R-genes was very weak and we have removed the relevant statements in the revised manuscript (see lines 371-373 and 539-543 in Results and Discussion of the revised MS).

To help place the results in context, it was requested to discuss the limitations of two replicates and how G x E can influence the results.

Response: Thank you for the comment. We agree that multi-year or multi-location experiments are important to evaluate the G x E effects of GWAS results for most complex traits of moderate or low heritability. However, for bacterial blight resistance, we believe one-year data is sufficient because of the following four reasons: (1) Rice responses to *Xoo* measured by lesion lengths using the leaf-clipping artificial inoculation method are known to be a highly heritable trait which are not affected by locations and years (Nayak et al., 1987; Li et al., 2001). This was also evidenced by the fact that all major R(*Xa*)-genes such as *Xa21*, *Xa23*, *Xa4* and *Xa26* were able to show high-level of resistance in different genetic backgrounds and environments across different geographic regions (Zhou et al., 2011; Luo et al., 2012). (2) As shown in the Manhattan plots (Figure 3), strong peak signals in different genomic regions (particularly on chromosome 11) harboring many reported/cloned R(*Xa*)-genes were consistently detected in different panel populations and in different studies (Zhang et al., 2017; Dilla-Ermita et al., 2017; Xie et al., 2015), demonstrating that our results are reliable. (3) In the PNAS paper by Wang et al. (2018), they also used one year data including 3 biological replicates with one plant as one replicate, while 5 (for the first set population) and 3 (for the second set populations) plants in each of two replications were used in our study. We are sorry that this was not clearly described in the method, which is now clarified in the revised manuscript (see lines 716-717, and 725-731). In fact, when compared with Wang et al. (PNAS, 2018), we had more replicates/plants in our study (10 replicates for the first set population and 6 for the second set population). The datasets of our study had many more plant x microbe genotype combinations (73 rice accessions × 23 *Xoo* strains × 5 plants × 2 replications = 16,790 in the first set population and 701 rice accessions × 4 *Xoo* strains × 3 plants × 2 replications = 16,824 in the second set population). This was much bigger than Wang et al.'s population, which consisted of 130 *A. thaliana* lines × 22 *X. arboricola* strains × 3 plants = 8,580. (4) One year of data with two replications are commonly used for GWA studies with large populations (see the Nature Genetics paper by Chen et al. 2014 and the PNAS paper by Xie et al. 2015).

To evaluate the effects of replication of our phenotypic data, we have included the ANOVA results of the two sets of materials (see lines 130-136 and lines 236-242 in the revised MS), which indicated that difference between the two replications was insignificant (P = 0.855 for the first set rice materials; P = 0.062 for the second set rice materials) in both sets of populations. The Pearson's correlation coefficient between two replications were also highly positive (correlation coefficient r ranged from 0.87 [P < 0.001] for P3b to 0.98 [P < 0.001] for P7 in the first set rice materials; r = 0.86, P < 0.001 for P1; r = 0.82, P < 0.001 for C3; r = 0.80, P < 0.001 for C5; r = 0.88, P < 0.001 for P9a in the second set rice materials), and the heritability estimates for lesion lengths for all *Xoo* strains were all high (>0.79). To better address the reviewer#4's concern, the best linear unbiased predictions (BLUPs) for the lesion lengths of the two replications were calculated with the lmer function of the R package lme4, using rice accessions as the random effect and replication as the fix effect (to accounted for the effects of replication). The BLUPs were perfectly and positively correlated with the mean lesion lengths in both sets of populations (Pearson's correlation coefficient r = 1.00, P < 0.001) (Supplemental Data Set 4). Furthermore, we re-conducted the GWA using the lesion lengths of each replication and BLUPs of two replications as input data in the second set population, and found the results based on BLUPs and means were very consistent (see the following Figure shown in the response to the reviewer#4's relevant concern). The GWA results of two replications were also consistent, particularly for those statistically significant peak signals, suggesting that 'replication' had no obvious effect on the GWAS results. We have included this point in Method section of the revised manuscript (see lines 878-884).

These results indicated that two replications for measuring the lesion lengths by the leaf-clipping artificial inoculation were sufficient for evaluating the quantitative responses of rice accessions to specific *Xoo* strains. We also added a brief discussion (lines 546-549) and relevant references in the revised manuscript, as the editor suggested.

References:

Nayak, P., Suriya Rao, A.V., and Chakrabarti, N.K. (1987) Components of resistance to bacterial blight disease of rice. J. Phytopathology 119, 312-318.

Li, Z.K., Sanchez, A., Angeles, E., Singh, S., Domingo, J., Huang, N., and Khush, G.S. (2001). Are the dominant and recessive plant disease resistance genes similar? A case study of rice R genes and *Xanthomonas oryzae* pv. *oryzae* races. Genetics 159, 757-765.

Zhou, Y.L., Uzokwe, V.N.E., Zhang, C., Cheng, L., Wang, L., Chen, K., Gao, X., Sun, Y., Chen, J., Zhu, L.,

Zhang, Q., Ali, J., Xu, J., and Li, Z. (2011). Improvement of bacterial blight resistance of hybrid rice in China using the *Xa23* gene derived from wild rice (*Oryza rufipogon*). Crop Protect. 30, 637-644.

Luo, Y., Sangha, J.S., Wang, S., Li, Z., Yang, J., and Yin, Z. (2012). Marker-assisted breeding of *Xa4*, *Xa21* and *Xa27* in the restorer lines of hybrid rice for broad-spectrum and enhanced disease resistance to bacterial blight. Molecular Breeding 30, 1601-1610.

Zhang, F., Wu, Z., Wang, M., Zhang, F., Dingkuhn, M., Xu, J., Zhou, Y., and Li, Z. (2017). Genome-wide association analysis identifies resistance loci for bacterial blight in a diverse collection of *indica* rice germplasm. PLoS ONE 12(3): e0174598.

Dilla-Ermita, C.J., Tandayu, E., Juanillas, V.M., et al. (2017). Genome-wide association analysis tracks bacterial leaf blight resistance loci in rice diverse germplasm. Rice 10: 8.

Chen, W., Gao, Y., Xie, W., Gong, L., et al. (2014). Genome-wide association analyses provide genetic and biochemical insights into natural variation in rice metabolism. Nature Genetics 46, 714-721.

Xie, W., Wang, G., Yuan, M., Yao, W., et al. (2015). Breeding signatures of rice improvement revealed by a genomic variation map from a large germplasm collection. PNAS 112, E5411-E5419.

Please, clarify N in all datasets.

Response: Thanks for your comment and we apologize for the unclear description in the previous manuscript. We clarified the sample size (*N*) in all relevant datasets and figures according to the editor's comment (see Figures 2-6, Supplemental Figures 7, 9, 11, Supplemental Table 4, and Supplemental Data Sets 1, 4 and 9).

It was noted that the TE statements do not seem supported by data in the manuscript and should be removed.

Response: Thanks for the kind comment and we agree with the reviewer#4 that the observed number of TEs would be compared against the expected number by random chance. Based on the MSU v7 annotation, there are 16,941 TEs in the whole Nipponbare reference genome, accounting for 30.26% (16,941/55,986) of all genes. In this study, we found 15.2% (827/5432) SNPs in 95 (29.9%) TEs out of 318 detected genes. Fisher's exact test shows that the probabilities of TEs associated with resistance to *Xoo* showed no significant difference from the genomic background (*P* = 0.9534). We also agree that GWAS is not precise enough to identify causative QTN. Thus, we removed the relevant statements in the revised Discussion section.

Finally, please work to connect to the existing literature on race structure and causality better to incorporate this work into the existing data.

Response: Thank you for the comment. As the editor suggested, we have compared the *Xoo* race structure (Figure 1B and 1C) of the Philippine races depicted by the SNP data with the previously reported one (Quibod et al., 2016) and found the results were very consistent for most Philippine races. We also noted the major difference was that six weak virulence Chinese races (C1, C2, C3, C4, C6 and C7) plus two Philippine ones (P1 and P9d) formed a separate cluster plus three admixtures (P10, GIV and P4). In our study, the *Xoo* race structure depicted by the SNP data (Figures 1B and 1C) was similar to the grouping by the phenotypic data (Figure 1A), suggesting that the selection pressure from the host was the driving force shaping the *Xoo* race structure. This causality is in agreement with the previous study on Philippine races (Quibod et al., 2016) that diversification of effector repertoires during host adaptation should be an important drive for *Xoo* race structure of the Philippine archipelago. We have discussed the results on the race structures in connection to the current literature in the first paragraph of the revised Discussion (see lines 484-499).

We also noted an inconsistency in the naming systems used in our study and literature, in which the strain codes (the PXO number) were used in some cases, while the race names were used in others (including this study). We actually used the race names because they were obtained based directly based on their differential responses to two sets (one from IRRI and another from China) of isogenic lines and contained information regarding the timing they emerged (were collected) such that small number (or alphabetical letters) always came earlier for the tropical *Xoo* strains. To avoid confusion and connect our work with the existing literature, we replaced 'strain' with 'race' in the relevant text of the revised manuscript, and included both the race names and strain codes (PXO#) of *Xoo* in all relevant Figures (Figures 1-4).

Reviewer #1:

This manuscript is a re-submission of one that I have reviewed before, documenting an analysis of the genetic interactions between rice and the bacterial pathogen *Xanthomonas oryzae* (*Xoo*). The manuscript describes an experiment of impressive scale that discovers a number of genes in both species that appear to be

important for variation in pathogenicity, and a number of genetic interactions that may be useful going forward for identifying novel mechanisms. I have focused on the GWAS analyses for this review, leaving the pop-gen analysis for the other reviewers.

We have discussed at length in previous versions the limitations of the study, particularly the limited size of the *Xoo* panel for association analyses. This remains an issue, but doing more would be prohibitively difficult, and I think the authors have done a good job with their experimental design and analysis of accounting for the limited size as well as they can. The association analyses are documented clearly with the decisions made at each step explained and justified. I remain concerned that some of the identified *Xoo* genes are false positives due to population structure, and would be curious to know how many of the associations were detected by the LMM component of the CAS method (which should be more robust to structure) vs only with the LM component (which is probably less robust, but the effect should be tempered by the 1/K_j term). My understanding of the method is that a SNP could reach significance only based on LM results ignoring structure. However, I agree that there are limitations of both methods, so I am comfortable with the justifications provided by the authors here.

I think that the new analysis of the pairwise interactions is much more clear and more useful than the original analysis, and am generally satisfied by the methods here, though I have a couple of clarification questions on this that I list below.

> **Response: Thank you for the comments. We had the same concern when we first used the CAS method. Our first-generation CAS analysis was built purely on the LMM detected associations and was then extended to the current CAS model combining both LM and LMM, which resulted in identification of the 86 significant SNPs (CAS_{LM+LMM} >0.3). When only the LM or LMM method (CAS_{LMM}>0.15) was used, 67 or 57 of the 86 SNPs were detected with 38 SNPs are overlapped between the two methods. Thus, LMM and LM generally produce consistent results, but each of them does produce some unique significant variants. This was the primary reason to combine both the methods in our final CAS model to reduce the false negative rate. In addition, false discovery rate (0.17) calculated from the permutation analysis suggested previously by the reviewer provided an unbias evaluation of the accuracy.**

Comments and points for clarification:

902: I'm not 100% clear here how you're getting more than 1 rice SNP per LD block. Do the multiple SNPs per block have LD >0.5 or <0.5?

> **Response: Sorry for the unclear description. The primary reason why more than 1 rice SNP per LD block obtained in this study was that different lead SNPs (i.e. with lowest *P*-values in the original GWAS) were observed for the four *Xoo* races at several LD blocks. We agree with the reviewer's previous comment that if a *P*-value is low enough, lower *P*-values mean "more evidence that the effect is real", but not more evidence that the effect is "important". Therefore, according to the reviewer's previous suggestion to determine rice tag SNPs within LD blocks for further cross-species interaction analysis, we selected two representative SNPs for each *Xoo* race that one with the lowest *P*-values in one-dimensional GWAS (i.e. the SNP identified by the clump function in PLINK) and one with the largest contribution to the phenotypic variance estimated using ANOVA with the statistical model described by Zhao et al. (2011) within each LD block. The lead SNP (with the lowest *P*-value) was selected as the tag SNP when the two SNPs were in high LD (r^2 > 0.5). Otherwise, both the two SNPs were considered as independent tag SNPs for this LD block to conduct further cross-species interaction analysis. Thus, the multiple tag SNPs per rice LD block had LD <0.5 (r^2). Furthermore, there were cases where the significantly strongest association was different for the four *Xoo* races within a rice LD block. That was the reason why we retained more than 1 rice SNP per LD block in some cases (see Supplemental Data Set 6). We rewrote the relevant Method description to make it clearer in the revised manuscript (see lines 937-952). We sincerely appreciate the reviewer's previous suggestion of using LD blocks, instead of using large numbers of significant SNPs, in our 2-dimensional GWAS, which allowed us to detect more important pairwise interactions described in the revised manuscript.**

904: Is this the same ANOVA as described above in the 2-way GWAS? Or in the original GWAS? How is this different from the clump function in plink used above?

> **Response: Thanks for your comment and we apologize for this unclear description in the previous manuscript. The statistical model of ANOVA used for estimating the contribution to the total phenotypic variance by the SNP was the one described by Zhao et al. (2011), which was different from the two-way GWAS and the original GWAS. And the clump function in PLINK was used for determining the independent LD blocks and lead SNPs (i.e. with lowest *P*-values in the original GWAS). Specifically, we identified the lead SNP based on the clump function in PLINK and the LD between the lead SNP and the SNP with largest**

contribution to the phenotypic variance within an LD block for further cross-species interaction analysis. We did this according to the reviewer's previous comment so that the significant SNPs with the largest effect but not the lowest *P*-value would not be missed. The lead SNP (with the lowest *P*-value) was selected as the tag SNP when the two SNPs were in high LD (r^2 > 0.5). Otherwise, both the two SNPs were considered as independent tag SNPs for this LD block to conduct further cross-species interaction analysis. In majority of the GWA blocks (51.3%), the lead SNP and the SNP of largest contribution to the phenotypic variance were same. We rewrote the relevant Method description to make it clearer in the revised manuscript (see lines 937-952).

Reference:

Zhao, K., Tung, C., Eizenga, G.C., Wright, M.H., Ali, M.L., Price, A.H., Norton, G.J., Islam, M.R., Reynolds, A., Mezey, J., McClung, A.M., Bustamante, C.D., and McCouch, S.R. (2011). Genome-wide association mapping reveals a rich genetic architecture of complex traits in *Oryza sativa*. Nat. Commun. 2, 467.

696: should be lme4.

Response: Thank you for pointing out this typing mistake. We have corrected it in the revised manuscript (see line 752).

258: For the rice SNPs detected only in the Xian or Geng subpopulations, were these SNPs at high enough frequency in the other population to be detectable? If you do an interaction test of SNP x sub-population, do you have evidence that the effect is actually different between these subpopulations?

Response: Thanks to the Reviewer's professional suggestion. Our answer to the first question is 'No' for 41.1% of the SNPs detected only in the *Xian* subpopulation, and 48.2% of the SNPs detected only in the *Geng* subpopulation. Specifically, of the 3,411 SNPs detected only in *Xian*, 1,402 SNPs (41.1%) were rare alleles (minor allele frequency < 0.05) in *Geng*. Similarly, of the 245 SNPs detected only in *Geng*, 118 (48.2%) SNPs were rare alleles of low frequency (< 0.05) in *Xian*. According to the reviewer's suggestion, we performed the interaction test of SNP x sub-population by two-way ANOVA. 414 (11.3%) of these subpopulation-specific SNPs were fixed in one of the subpopulations, so that we could not estimate the SNP x sub-population interaction effect. For the remaining 3,242 SNPs, 2,482 (76.6%) SNPs were detected with significant interaction effects between SNP genotype and subpopulation, indicating that most of these subpopulation-specific SNPs have different effects between the two subpopulations. This result answers your question that most significant SNPs had differentiated effects in different subpopulations (we have included this result in the revised manuscript, see lines 291-296, and updated Supplemental Data Set 5), consistent with our GWAS results using *Xian* and *Geng* populations separately, which led to the conclusion that rice QR-genes for resistance to *Xoo* was largely subspecies specific.

Did you try associations with the pan-genome (i.e. presence / absence of each gene as a marker)? Otherwise, how was this analysis used?

Response: Thanks for pointing this out. The pan-genome results were integrated here to answer a previous question raised by another reviewer regarding the *Xoo* population structure. We showed that the consistent population structures were obtained based on both SNPs (revised Figure 1B) and gene presence/absence (gene PAV) data. We decided not to perform GWA using the gene PAV data based on the following two reasons. First, the current gene PAV datasets of both rice and *Xoo* were obtained from the NGS sequencing data and remain to be validated based on our recent analyses of 100 high-quality rice reference genomes from the third-generation sequencing (TGS) technology. This was because a significant portion of annotated genes from the NGS data could not be validated by the TGS dataset of the same set of materials (data not shown). Thus, misleading results may come from the inaccurate gene annotations and the sequencing gaps (incomplete genome coverages) of the current rice gene PAV data. This was particularly true for the *Xoo* gene PAV data given its small sample size, which make it more difficult to apply the customized CAS strategy. Secondly, given the large numbers of SNPs used in our one-dimensional GWAS, we do not expect the gene PAV datasets will result in many novel discoveries because most, if not all, associations detected by gene PAV datasets would have been detected by their closely linked SNPs if they are important ones. Thus, we have removed the relevant results of the gene PAV in the revised manuscript.

843: Were the results of SnpEff used? Since you're clumping SNPs into regions, there's really no sense in also reporting the specific predicted effect of the tag SNP itself.

Response: Thank you for your reminder. This is a question we missed in the previous revision after clumping SNPs into regions, and the results of SnpEff were not used. We agree with the reviewer that reporting the specific predicted effect of the tag SNP itself is no sense. Follow the reviewer's suggestion, we have removed the relevant description about SnpEff in the Methods section of the revised manuscript.

182-183: what is the difference between the 48 "known virulence-related genes" and the 18 "other virulence-related genes"?

Response: Sorry for the unclear description in the previous manuscript. The "known virulence-related genes" were those cloned genes previously reported in *Xoo*, whereas "other virulence-related genes" were defined as those newly detected in this study and with evidence to be highly likely virulence-associated based on the literature on other related bacterial species. To avoid confusion, we changed "other" to "highly likely" and added more description to make it clear in the revised manuscript (see lines 194-198). And we also provided the classification of these genes in Supplemental Table 3. Thank you.

Reviewer #2:

The authors have performed substantial revisions compared to previous versions of this manuscript. The reworded manuscript also largely avoids conflating different concepts. The detailed analyses of LD and recombination rates in *Xoo* genomes cannot fully alleviate concerns, in particular in regards to the high degree of LD outside of the recombination hotspot at 1.57-1.86 Mb. However, the new analyses and representations provide a transparent view on potential issues.

Remaining concern:

The recombination hotspot(s) identified between 1.57 Mb-1.86 Mb in the *Xoo* genomes make(s) this region particularly suited for association mapping despite the low sample size. However, the rest of the genome is largely untouched by recombination. This also means that arguing that the average recombination rate is comparable to e.g. Fusarium graminearum is not very meaningful. For the manuscript, the authors should clearly state:

(1) that the mapping power is only really high near this recombination hotspot and (2) clearly state that ability to detect reliable associations outside of this recombination hotspot is clearly reduced. A brief classification of the associations near or far from the hotspot(s) should be added to the manuscript. This can be done by adding some sentences at the appropriate place.

Response: Thanks for the kind comment and we agree with the reviewer that the level of recombination has been an influencing factor of GWAS. We found that the virulence-related SNPs were significantly enriched (Fisher's exact test, *P* = 0.009 [the recombination hotspot vs. the rest of the genome]) in this region of high recombination, suggesting that the ability to detect reliable associations is higher near this recombination hotspot than outside of this recombination hotspot. Furthermore, we have supplemented the LD decay without the ~290 Kb region of high recombination (1.57-1.86 Mb) in the *Xoo* genomes (see Supplemental Figure 1B) according to the editor's comment. We observed similar LD decay and expected smaller average LD blocks (~2.1 kb) for SNPs with the recombination hotspot as compared to the SNPs without the recombination hotspot (~2.4 kb). This small difference in the average LD block sizes within and outside the recombination hotspot had little impact on the number and resolution of the detected virulence-related genes in both cases (Fisher's exact test, *P* = 0.119 [the recombination hotspot vs. the rest of the genome]), though we indeed observed a higher power (significantly more virulence-related SNPs in the recombination hotspot (Fisher's exact test, *P* = 0.009 [the recombination hotspot vs. the rest of the genome]). We have marked the recombination hotspot on the *Xoo* genome in Figure 7A and Supplemental Table 2 so that readers could clearly see the virulence-related genes within and outside the recombination hotspot. We also included the relevant descriptions in the revised manuscript (see lines 166-171 and lines 186-191). According to the reviewer's comment, we have removed the reference to *Fusarium graminearum* in the revised manuscript. Please also see our response to the question #1 of Editor.

Reviewer #4:

In this manuscript, the authors conduct a GWAS in rice for *Xoo* resistance and conduct a two species GWAS. They identify 41 genomic regions associated with resistance and 47 virulence related genes in *Xoo*. They identify possible regions involved in the interactions between the plant and pathogen.

Major comments

1. The phenotypic data for the large-panel GWAS consists of one year of data with two replications and four isolates. Two replications in one year seems insufficient, as it is important to see consistent genetic signal across environments. The number of environments/replications is not acceptable for the two species GWAS

either. Wang et al. 2018 PNAS used three replications grown in a controlled environment setting and had a higher number of plant * microbe genotype combinations. It is not clear from the methods whether the phenotypic data was merely averaged or whether the effects of replication were accounted for in the means. An ANOVA is mentioned in relation to calculating heritabilities, but from lines 674-676 it sounds like ratings were only averaged. Was replication significant? More information should be included about the experimental design and variation of the field trials.

Response: Thank you for the comment. We agree that multi-year or multi-location experiments are important to evaluate the G by E effects of GWAS results for most complex traits of moderate or low heritability. However, for bacterial blight resistance, we believe one year data is sufficient because of the following four reasons: (1) Rice responses to *Xoo* measured by lesion lengths using artificial inoculation of the leaf clipping method are known to be a highly heritable trait which are not affected by locations and years (Nayak et al., 1987; Li et al., 2001). This was also evidenced by the fact that all major R(*Xa*)-genes such as *Xa21*, *Xa23*, *Xa4* and *Xa26* were able to show high-level of resistance in different genetic backgrounds and diverse environments across different geographic regions and years (Zhou et al., 2011; Luo et al., 2012). (2) As shown in the Manhattan plots (Figure 3), strong peak signals in different genomic regions (particularly on chromosome 11) harboring many reported/cloned R(*Xa*)-genes were consistently detected in different panel populations and in different studies (Zhang et al., 2017; Dilla-Ermita et al., 2017; Xie et al., 2015), demonstrating that our results are reliable. (3) In the PNAS paper by Wang et al. (2018), they also used one year data including 3 biological replicates with one plant as one replicate, while 5 (for the first set population) and 3 (for the second set population) plants in each of two replications were used in our study. We are sorry that this was not clearly described in the method, which is now clarified in the revised manuscript (see lines 716-717 and lines 725-731). In fact, when compared with Wang et al. (PNAS, 2018), we had more replicates/plants in our study (10 replicates for the first set population and 6 for the second set population). The datasets of our study had many more plant x microbe genotype combinations (73 rice accessions × 23 *Xoo* strains × 5 plants × 2 replications = 16,790 in the first set population and 701 rice accessions × 4 *Xoo* strains × 3 plants × 2 replications = 16,824 in the second set population). This was much bigger than Wang et al.'s population, which consisted of 130 *A.* thaliana lines × 22 *X.* arboricola strains × 3 plants = 8,580. (4) One year of data with two replications are commonly used for GWA studies with large populations (see the Nature Genetics paper by Chen et al. 2014 and the PNAS paper by Xie et al. 2015).

In our analysis, we did not use the disease rating but the mean lesion length (in cm) of the two replications for GWAS. The primary reasons why we used the mean lesion lengths of the two replications for each accession as the input phenotypic data in our GWAS were explained above. Also, mean values across replications of phenotypes were often used in previous rice GWAS reports (such as Zhao et al., 2011; Guo et al., 2020). According to the reviewer's suggestion, we have included the ANOVA results of the two sets of materials in the revised manuscript (see lines 130-136 and lines 236-242) to evaluate the effects of replication of our phenotypic data. The ANOVA results (see the following Tables Sa and Sb) indicated that difference between the two replications was insignificant (P = 0.855 and 0.236; R^2 = 0% and 0.003%) in both sets of populations, and the Pearson's correlation coefficient between two replications are also highly positive (correlation coefficient r ranged from 0.87 [P < 0.001] for P3b to 0.98 [P < 0.001] for P7 in the first set population; r = 0.86, P < 0.001 for P1; r = 0.82, P < 0.001 for C3; r = 0.80, P <0.001 for C5; r = 0.88, P < 0.001 for P9a in the second set population), while the heritability estimates for lesion lengths for all *Xoo* races were high (>0.79). Furthermore, the best linear unbiased predictions (BLUPs) for the lesion lengths of the two replications were calculated with the lmer function of the R package lme4, using rice accessions as the random effect and replication as the fix effect (to accounted for the effects of replication). The BLUPs were perfectly and positively correlated with the mean lesion lengths in both sets of populations (Pearson's correlation coefficient r = 1.00, P < 0.001) (Supplemental Data Set 4). We re-conducted the GWA using the lesion lengths of each replication and BLUPs of two replications as input data in the second set population, an found the results based on BLUPs and means were very consistent (see the following Figure). The GWA results of two replications were also consistent, particularly for those statistically significant peak signals, suggesting that 'replication' had no obvious effect on the GWAS results. We have included this point in Method section of the revised manuscript (see lines 878-884).

These results indicated that two replications for measuring the lesion lengths by the leaf-clipping artificial inoculation were sufficient for evaluating the quantitative responses of rice accessions to specific *Xoo* strains. We sincerely hope the reviewer could accept our explanation.

Table Sa. The analysis of variance of the data of the first set rice materials

Source of variation	df	SS	MS	F	*P*-value	R^2 (%)

Replications	1	0	0.1	0.034	0.855	0.000
Rice	72	114438	1589	552.9	<2e-16	52.2
Xoo	22	39826	1810	629.7	<2e-16	18.1
Rice × *Xoo*	1560	60418	38.7	13.5	<2e-16	27.5
Residuals	1654	4755	2.9			

48 observations deleted due to missingness.

Table Sb. The analysis of variance of the data of second set rice materials

Source of variation	df	SS	MS	F	*P*-value	R^2 (%)
Replications	1	9	9	1.4	0.236	0.003
Rice	700	128333	183	27.6	<2e-16	46.1
Xoo	3	48380	16127	2427.7	<2e-16	17.4
Rice × *Xoo*	1995	83971	42	6.3	<2e-16	30.2
Residuals	2663	17690	7			

245 observations deleted due to missingness.

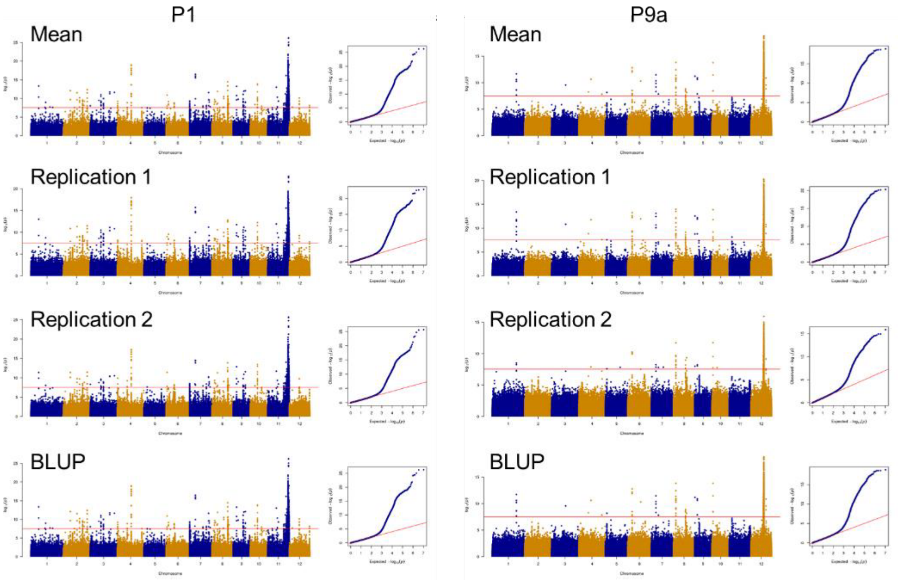

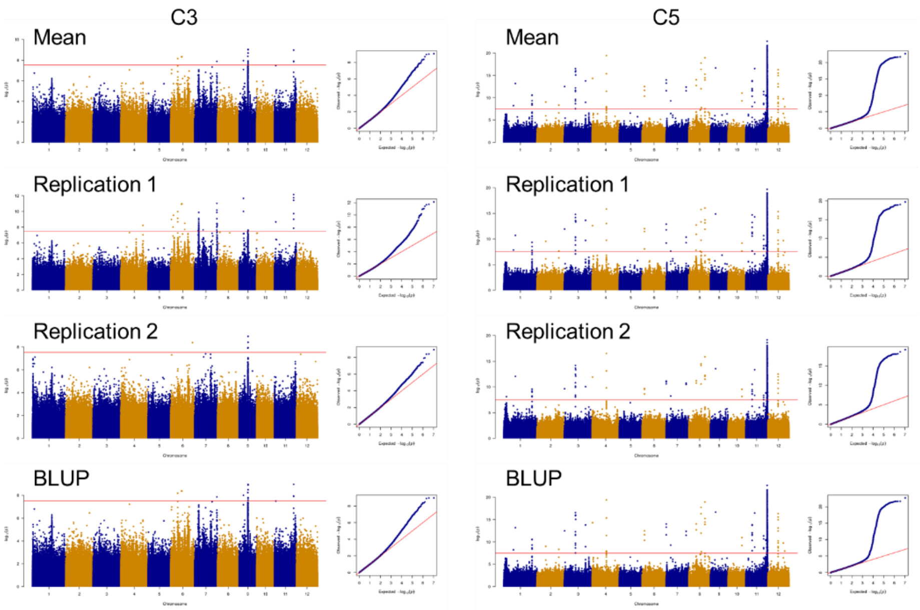

Figure. The Manhattan plots of genome-wide associations with bacterial blight resistance to four *Xoo* races (P1, P9a, C3, and C5) in whole panel using the lesion lengths of replication 1, replication 2, their mean value, and BLUP, respectively.

References:

Nayak, P., Suriya Rao, A.V., and Chakrabarti, N.K. (1987) Components of resistance to bacterial blight disease of rice. J. Phytopathology 119, 312-318.

Li, Z.K., Sanchez, A., Angeles, E., Singh, S., Domingo, J., Huang, N., and Khush, G.S. (2001). Are the dominant and recessive plant disease resistance genes similar? A case study of rice R genes and *Xanthomonas oryzae* pv. *oryzae* races. Genetics 159, 757-765.

Zhou, Y.L., Uzokwe, V.N.E., Zhang, C., Cheng, L., Wang, L., Chen, K., Gao, X., Sun, Y., Chen, J., Zhu, L., Zhang, Q., Ali, J., Xu, J., and Li, Z. (2011). Improvement of bacterial blight resistance of hybrid rice in China using the *Xa23* gene derived from wild rice (*Oryza rufipogon*). Crop Protect. 30, 637-644.

Luo, Y., Sangha, J.S., Wang, S., Li, Z., Yang, J., and Yin, Z. (2012). Marker-assisted breeding of *Xa4*, *Xa21* and *Xa27* in the restorer lines of hybrid rice for broad-spectrum and enhanced disease resistance to bacterial blight. Molecular Breeding 30, 1601-1610.

Zhang, F., Wu, Z., Wang, M., Zhang, F., Dingkuhn, M., Xu, J., Zhou, Y., and Li, Z. (2017). Genome-wide association analysis identifies resistance loci for bacterial blight in a diverse collection of *indica* rice germplasm. PLoS ONE 12(3): e0174598.

Dilla-Ermita, C.J., Tandayu, E., Juanillas, V.M., et al. (2017). Genome-wide association analysis tracks bacterial leaf blight resistance loci in rice diverse germplasm. Rice 10: 8.

Chen, W., Gao, Y., Xie, W., Gong, L., et al. (2014). Genome-wide association analyses provide genetic and biochemical insights into natural variation in rice metabolism. Nature Genetics 46, 714-721.

Xie, W., Wang, G., Yuan, M., Yao, W., et al. (2015). Breeding signatures of rice improvement revealed by a genomic variation map from a large germplasm collection. PNAS 112, E5411-E5419.

Zhao, K., Tung, C.W, Eizenga, G.C., Wright, M.H. et al. (2011). Genome-wide association mapping reveals a rich genetic architecture of complex traits in Oryza sativa. Nature Communications 2, 467.

Guo, H., Zeng, Y., Li, J., Ma, X., et al. (2020). Differentiation, evolution and utilization of natural alleles for cold adaptability at the reproductive stage in rice. Plant Biotechnology Journal 18, 2491-2503.

2. I am not clear on whether there were enough accessions included in the subpopulation GWAS analyses to lead to robust conclusions. It is not clear from the methods how many individuals were in each of the subpopulations in Figure 3. Perhaps this information is somewhere in the supporting data, but I was not able to locate it. To simplify, the number of samples should be included in Figure 3 for each the GWAS plots. Were some lines included in multiple subpopulation analyses? If each accession was only used once, presumably some of the Chinese or overseas accessions might group better with the *indica* or *japonica* subpopulations, rather than being placed in an arbitrary group. Furthermore, with 701 accessions there are too few accessions to adequacy divide the population into 7 subpopulations and have adequate numbers of individuals in each set of lines tested. The lack of signal in the Chinese landraces is curious. Based on Figure 2, there is phenotypic variation in the population. How many individuals were included in this analysis? I would expect since these are landraces, that there should be allelic diversity and there was phenotypic diversity. The authors give some reason for a lack of signal, but perhaps there is a technological reason for the lack of signal.

Response: Thanks for the comment. We have included the number of accessions for each of the GWAS plots in revised Figure 3 and added more description to make the sample size of each subpopulation clear in the Results and Methods section (see lines 246-254 and lines 738-741). Yes, some accessions were included in multiple populations (see the following Figure i). The grouping of the subpopulations was not arbitrary. In fact, the primary reason we included a large sample of Chinese modern varieties (absent in the 3KRGP) in this study was to provide sufficiently large sample sizes for two comparisons between Chinese rice accessions (*n*=451) and overseas ones (*n*=241), and between Chinese landraces (CHN-LAN) (*n*=135) and Chinese modern varieties (CHN-MV) (*n*=316) to answer the related questions regarding sources of QR-genes and the breeding impact on the co-adaptation between rice and *Xoo*. Our result that few QR-genes (only two loci for resistance to *Xoo* race C3) were detectable in the CHN-LAN population could be attributed to at least three reasons. The first was its relatively small population size of 135 accessions, which would have a reduced power in detecting QR-loci by GWAS. The second one was its smallest portion of resistant accessions (19.3% for C3, 2.2% for P1, 1.5% for C5 and 9.6% for P9a) (see Figure 2B, 135 CHN-LAN individuals included in this analysis), as compared to other panel populations. Actually, we identified two association signals (Chr1_5757426 and Chr2_18983864) in the CHN-LAN only for C3 (Figure 3 and Supplemental Data Set 5) but no signals for the other three *Xoo* races, which was consistent with the phenotypic variation in the CHN-LAN population. The third one was that resistance of different accessions to a single *Xoo* race is possibly controlled by different QR-genes. Thus, the CHN-LAN population had a low power in detecting QR-genes. In fact, the reasons 2 and 3 appeared to be more important.

To test if the relatively small population size of 135 accessions was the primary reason for the reduced power in GWAS, we performed a computer simulation by randomly sampling the same number (*n*=135) of accessions from the CHN-MV population 100 times and performed GWAS separately. The results indicated that unlike the CHN-LAN population, large numbers of significant associations were detected in virtually all samples of the CHN-MV with the same population size for C3, P9a and P1 except for C5 (see the following Table). Even for *Xoo* strain C5, no significant association was detected in only 27% of the 100 samples. This result clearly indicated that the primary reason for the lack of signal of QR-genes in the CHN-LAN was due to its small portion of resistant accessions. This was not surprising since the CHN-LAN are known to lack of resistance to *Xoo* (Ou and Jennings 1969; Zhang, 2009). According to the extensive national efforts of China in screening 63,046 rice accessions for BB resistance during 1960s-1990s, only 2,399 (3.8%) showed high-level (LL < 3.0 cm) resistance to one or more Chinese *Xoo* races, most of which were from foreign accessions imported from IRRI (Zhang, 2007).

Additional argument came from the fact that strong associations in the 3.3 Mb region of R30 – R37 on rice chromosomes 11 and region of chromosome 12 where important R(*Xa*)-genes are known to reside, were consistently detected in all 100 times' random sampling (sample size *n* = 135) from the CHN-MV (see the following Figure ii for once random sampling and updated Figure 3). This indicates that the panel populations were suitable for detecting QR-genes by GWAS and strong signals could not be generated by random sampling. Also, some GWA studies using roughly equivalent population sizes have been reported in Nature (Liu et al., 2021), in which GWAS using 110 accessions resulted in identification of OsTCP19 for response to nitrogen use efficiency and using 176 accessions leading to discovery of four new genes associated with agronomic traits (Yano et al. 2016 Nature Genetics). Taking together, the lack of association signals in the CHN-LAN population was not due to a

technological reason or small sample size, but to the very low frequencies of QR-genes in the CHN-LAN population because most rice landraces in tropic Asia and China were known to lack of resistance to *Xoo* (Ou and Jennings 1969; Zhang, 2009). We have included this point in Discussion of the revised manuscript (see lines 481-483 and lines 506-515).

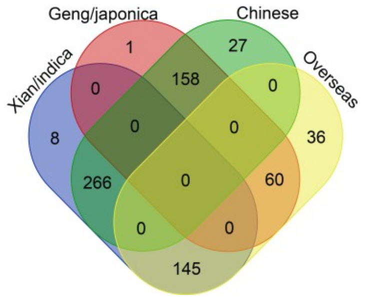

Figure i. The venn diagram showing the overlap among the four subpopulations according to population structure and origins. The number indicates the number of overlapping rice accessions among different subpopulations.

Table Summary of GWAS simulation results from 100 times' random sampling (sample size n = 135) in the Chinese modern variety population

Xoo race	Mean number of the detected significant SNPs	Standard deviation	Median number of the detected significant SNPs	Times of random sampling with no significant association	Probability of obtaining the observed GWAS results in CHN-LAN
C5	84	116.5	10	27	0.27
P9a	99.4	157.1	36	1	0.01
P1	57.2	78.7	18	5	0.05
C3	163	331.3	61.5	0	0

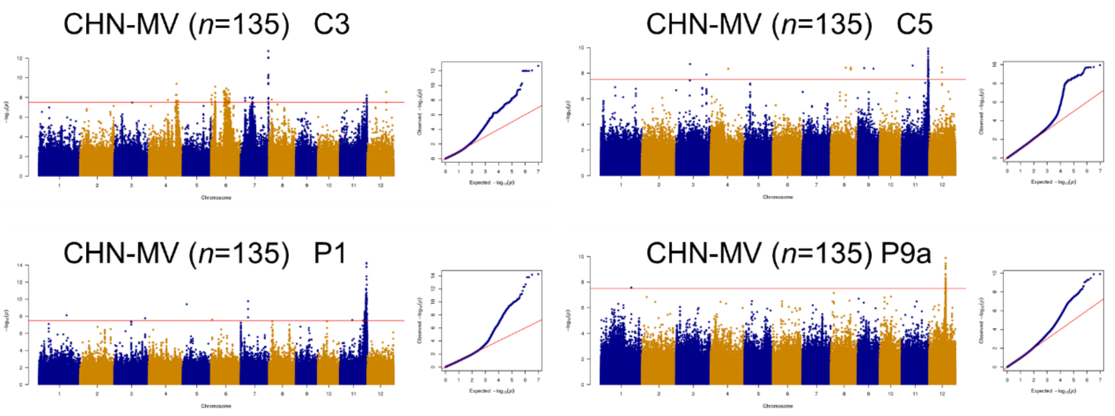

Figure ii. The GWAS results of one of the 100 times' random sampling (sample size n = 135) from the Chinese modern variety population. The Manhattan and QQ plots of genome-wide associations with bacterial blight

resistance to four *Xoo* races (C3, C5, P1, and P9a).

References:

Ou, S.H., and Jennings, P.R. (1969). Progress in the development of disease-resistant rice. Annu. Rev. Phytopathol. 7, 383-410.

Zhang, Q. (2009). Genetics and improvement of bacterial blight resistance of hybrid rice in China. Rice Sci. 16, 83-92.

Zhang Q. (2007). Genetics and Improvement of Resistance to Bacterial Blight in Rice.

Beijing: Science Press, China. (in Chinese)

Yano, K., Yamamoto, E., Aya, K., Takeuchi, H., Lo, P., Hu, L., Yamasaki, M., Yoshida, S., Kitano, H., Hirano, K., and Matsuoka M. (2016). Genome-wide association study using whole-genome sequencing rapidly identifies new genes influencing agronomic traits in rice. Nature Genetics 48, 927-934.

Liu, Y., Wang, H., Jiang, Z., Wang, W., Xu, R., Wang, Q., Zhang, Z., Li, A., Liang, Y., Ou,

S., Liu, X., Cao, S., Tong, H., Wang, Y., Zhou, F., Liao, H., Hu, B., and Chu, C. (2021). Genomic basis of geographical adaptation to soil nitrogen in rice. Nature 590, 600-605.

The authors state that there is epistasis among different types of *R* genes for novel resistance to *Xoo* (line 472-473). Their evidence is that most R-genes (more accurate to refer to as quantitative resistance genes?) were of small effect and that resistance could not be attributed to any large-effect *R* genes. However, they never show that the effects are not additive. It is common for resistance to be the sum of many small-effect QTL that result in a highly resistant line. There is no evaluation of whether the resistance is greater than what would be expected from additivity. To substantiate the epistasis claim, there needs to be statistical tests for epistasis. See Shang et al. 2011 BMC Bioinformatics for a summary of programs available to test for epistatic interactions.

Response: We fully agree with the reviewer that most detected *R*-genes by GWAS were probably quantitative in nature. However, many of them are indeed major *R*-genes of large effect detected by strong association signals (see the Manhattan plots of Figure 3). We also agree with the reviewer that high-level of resistance could be due to the sum of many small-effect QTLs. However, we did not provide the estimated additive effects of the detected *R*-genes or QTL because of three main reasons. First, for most detected QTL, there are many candidate genes for each detected QTL, and each of which contains a large number of functional alleles in the panel populations, given the current resolution of ~300 kb of the LD decay in the rice genome. Second, it is difficult to obtain accurate estimates of the additive effect(s) of any single detected *R*-genes (or QTL) without adequate control of genetic backgrounds, given the large numbers of loci involved and possible presence of epistasis between or among different R-genes. Thirdly, possible epistasis between or among different QTL cannot be adequately quantified in GWAS, even we tried the five methods proposed by Shang et al. (2011), as suggested by the reviewer, which did not produce consistent results. Furthermore, we found the methods for epistasis detection they suggested suffered the following problems: 1) inability to detect high-order (*n*>2, *n*=the number of loci involved in epistasis) epistasis; 2) inability to resolve the interactions among multiple alleles; 3) inadequate description of biological (functional) relationships between alleles in the epistasis (genetics) model (or difficulty to interpret); 4) lack of criteria for evaluation; and most importantly 5) difficulty to validate experimentally. Thus, we agree with the reviewer (and the editor) that our evidence of epistasis, suggested by the nonrandom association of the resistance alleles among 8 unlinked loci for high-level resistance of 19 accessions to SV race (C5), was weak. Thus, we have revised our interpretation on the observed association (see lines 371-373 and lines 539-543 in the revised MS).

3. The authors conclude that transposition of TEs are a mechanism for generating novel resistance based on the percentage of significant SNPs that are in TEs (lines 482-483). This isn't substantiated. What percentage of the genome is TEs? What percentage of the SNPs in the dataset were in TEs? Is 15.5% a deviation from what would be expected by random chance? Furthermore, GWAS is not precise enough, as the LD blocks can be large, to precisely locate causative polymorphisms.

Response: Thanks for the kind comment and we agree with the reviewer that the observed deviation of TEs should be expected by random chance. Based on the MSU v7 annotation, there are 16,941 TEs in the whole Nipponbare reference genome, accounting for 30.26% (16,941/55,986) of all genes. In this study, we found 15.2% (827/5432)

SNPs in 95 (29.9%) TEs out of 318 detected genes. Fisher's exact test shows that the probabilities of TEs associated with resistance to *Xoo* are equal to the other regions of rice genome (*P* = 0.9534). We also agree that GWAS is not precise enough to identify causative QTN. Thus, we removed the relevant description in the revised Discussion section.

4. Presumably the race structure of the isolates is driving some of the grouping the authors present for *Xoo*. The race/pathotype for the four strains of *Xoo* used should be mentioned. This would help contextualize the GWAS results. There are cloned *Xa* genes and it would be possible to check the haplotypes present in different groups of germplasm to confirm whether the cloned R genes are segregating or how the frequency differs across groups.

Response: Thank you for the comment. We have added the pathotypes of the four *Xoo* races before presenting the GWAS results (see lines 138-142, lines 233-235 and lines 254-262) according to the reviewer's suggestion. We already showed the haplotype analyses of 4 major *Xa* genes of large effect, including two cloned *Xa* genes, *xa25* and *Xa26*, plus two fine-mapped ones (*Xa40* and *Xa22* candidates) and their frequencies were provided in Figure 4 and Supplemental Figures 7 and 9. According to the reviewer's suggestion, we included the results of the haplotype analyses of the four major *Xa* genes and showed their frequency changes across different subpopulations (see lines 304-308, lines 316-320, lines 331-341, lines 357-365, and lines 402-421) in the revised manuscript. It should be pointed out that the most important genes of large-effect were detected in the ~3.3 Mb region of R30-R37 on rice chromosome 11 where many major R(*Xa*)-genes reside. This region has been the primary target of selection during modern breeding. We did not detect association signals with other cloned R(*Xa*)-genes, and thus could not perform relevant haplotype analyses.

5. It would be useful to present a principal component analysis of the bacterial strains in Figure 1. Race structure might be driving some of the grouping in Figure 1A and having a PCA in this figure would be helpful for accessing that. Because of major gene interactions, the phenotypes presented in Figure 1A might not be reflective of the genetic structure. This is somewhat suggested by Figure 1B. I would have liked to learn more about how this matches the race structure of the pathogen strains used. Have certain *Xa* genes been deployed more extensively in some regions compared to other regions that has driven race structure in the pattern that is observed in Figure 1?

Response: Thank you for the comment. According to the reviewer's suggestion, we performed the principal component analysis via EIGENSOFT using pruned SNPs computed with PLINK, which resolved the 23 *Xoo* strains into 4 major clusters (updated Figure 1B and 1C), similar to the grouping based on the reaction type (disease rating) (Figure 1A), except that the three tropical *Xoo* race groups each contains both several highly related moderate-virulence (II) and strong-virulence (III) races. This result suggests that the selection pressure from the host was the driving force shaping the *Xoo* race structure, which is in agreement with the previous study on Philippine races (Quibod et al., 2016). We have included the relevant result and description (lines 148-156 and lines 484-499) in the revised manuscript.

According to the literature (Mew et al., 1992; Zhang, 2009), the only *Xa* gene that was deployed extensively in both Philippines and China was *Xa4* which also locates in the ~3.3 Mb region of R30-R37, closely linked to *Xa26. Xa4* was cloned only recently and absent in the Nipponbare Reference genome which we used for calling SNPs in our genomic analyses. There is no corresponding gene ID for *Xa4* in any of the public databases. Thus, we could not perform the haplotype analysis for *Xa4*. Because *Xa4* locates in the peak region on chromosome 11 where 55% of the significant SNPs and 44% of non-transposon genes showing significant frequency shifts towards resistance alleles, including 7 fine-mapped (*Xa22*, *Xa32*, *Xa35*, *Xa36* and *Xa40*) /cloned R-genes (*Xa4* and *Xa26*) reportedly reside. Thus, we were able to reach the conclusion that artificial selection of modern breeding acting on the major *Xa* genes locate in the ~3.3 Mb region of R30-R37 were the primary driving force shaping the observed *Xoo* race structure (Figure 1). These results have been included in lines 382-386 and lines 402-421 of the revised manuscript.

Updated Figure 1. The origin of 23 diverse *Xanthomonas oryzae* pv. *oryzae* (*Xoo*) strains and their virulence. (A) Reaction type of 73 rice accessions caused by 23 *Xoo* strains from China and the Philippines and classification of the 23 *Xoo* strains into four major groups based on their virulence levels (mean lesion length [LL]): resistant (LL < 3cm), moderately resistant (3cm ≤ LL< 5cm), moderately susceptible (5cm ≤ LL < 10cm), susceptible (10cm ≤ LL < 15cm) and highly susceptible (LL ≥ 15cm). (B) Principal component analysis plots for the first two principal components of the 23 *Xoo* genomes. (C) The population structure and geographic distribution of the 23 *Xoo* strains. The neighbor-joining tree was constructed from LD pruned SNPs. Fraction ancestry was calculated with STRUCTURE software using an ancestry number of 3, which is shown in different colors.

Minor comments:

1. The predominant nomenclature for rice subpopulations should be used. *Indica* and *japonica* should be used instead of *Xian* and *Geng*.

   Response: We agree with the reviewer that the predominant nomenclature for rice has been *indica* and *japonica*. However, most of our materials come from the 3,000 Rice Genomes Project (3KRGP), in which (the Nature paper of Wang et al. 2018), *Xian* (*indica*) or *XI* and *Geng* (*japonica*) or *GJ* were used, and so were in this manuscript. Since then, the 3KRGP materials have been widely used by many research groups worldwide, including more than 60 groups of researchers in China. All publications came from the uses of the 3KRGP materials used *Xian* or *XI* and *Geng* or *GJ* (see the papers of Guo et al., 2020; Zhang et al., 2021; Liu et al., 2021). The primary reason why to do so is that several large databases have been established to include all the sequence data of the 3KRGP materials and accumulative phenotypic data of a wide range of traits in the public domain to facilitate future global efforts in rice functional genomics research and breeding by design. Thus, we prefer to stick on the nomenclature system of the 3KRGP in these databases to avoid the confusion of future users. Also, the original nomenclature was misleading because there is solid evidence that *japonica* was not originated from Japan, nor were all *indica* accessions originated from India.

   References:

   Wang, W., Mauleon, R., Hu, Z., Chebotarov, D., Tai, S., et al. (2018). Genomic variation in 3,010 diverse accessions of Asian cultivated rice. Nature 557, 43-49.

   Guo, H., Zeng, Y., Li, J., Ma, X., et al. (2020). Differentiation, evolution and utilization of natural alleles for cold adaptability at the reproductive stage in rice. Plant Biotechnology Journal 18, 2491-2503.

   Zhang, F., Wang, C., Li, M., Cui, Y., et al. (2021). The landscape of gene-CDS-haplotype diversity in rice: properties, population organization, footprints of domestication and breeding, and implications for genetic improvement. Molecular Plant. DOI: https://doi.org/10.1016/j.molp.2021.02.003.

   Liu, Q., Lan, G., Zhu, Y., Chen, K., et al. (2021). Genome-wide association study on resistance to rice black-streaked dwarf disease caused by *Rice black-streaked dwarf virus*. Plant Disease. Plant Disease 105, 607-615.

2. Line 36- some of these could be quantitative disease resistance genes. Please consider the differentiation between R genes and quantitative resistance genes throughout the manuscript.

   Response: Yes, we agree the reviewer that the nature of rice resistance to *Xoo* should be quantitative rather than qualitative, even though *R*-genes differ greatly in their effect magnitude against a single *Xoo* race, and even more so when against multiple *Xoo* races. Thus, it is really difficult to classify which ones should be considered as R-genes and which ones should be treated as quantitative resistance loci. According to the standard rice evaluation system developed at IRRI (the most widely used), the responses of rice plants to *Xoo* are classified into five categories: resistant (LL < 3.0 cm), moderately resistant (3.0 cm ≤ LL < 5.0 cm), moderately susceptible (5.0 cm ≤ LL < 10.0 cm), susceptible (10.0 cm ≤ LL < 15.0 cm), and highly susceptible (LL ≥ 15.0 cm). Obviously, this classification system is arbitrary. Thus, we have changed all R-genes into QR-genes (quantitative resistance genes) except for the previously reported *Xa* genes (including the cloned and fine-mapped ones) throughout the revised manuscript, according to the reviewer's suggestion.

3. Line 696- what is the lem4 package? The citation is for the lme4 package.

   Response: Thank you for pointing out this typing mistake. We have corrected it as lme4 in the revised manuscript (see line 752).

TPC2020-LSB-01058R1 2nd Editorial decision – *acceptance pending* April 26, 2021

We are pleased to inform you that your paper entitled "The Complex Genetic Interaction Systems Leading to Reciprocal Adaptation of Rice and *Xanthomonas oryzae* pv. *oryzae* Revealed by Cross-species Two-dimensional GWAS" has been accepted for publication in The Plant Cell, pending a final minor editorial review by journal staff.

Final acceptance from Science Editor May 15, 2021
